# Statistical Power of Model Selection Strategies for Genome-Wide Association Studies

**DOI:** 10.1371/journal.pgen.1000582

**Published:** 2009-07-31

**Authors:** Zheyang Wu, Hongyu Zhao

**Affiliations:** 1 Department of Epidemiology and Public Health, Yale University School of Medicine, New Haven, Connecticut, United States of America; 2 Department of Genetics, Yale University School of Medicine, New Haven, Connecticut, United States of America; University of Arizona, United States of America

## Abstract

Genome-wide association studies (GWAS) aim to identify genetic variants related to diseases by examining the associations between phenotypes and hundreds of thousands of genotyped markers. Because many genes are potentially involved in common diseases and a large number of markers are analyzed, it is crucial to devise an effective strategy to identify truly associated variants that have individual and/or interactive effects, while controlling false positives at the desired level. Although a number of model selection methods have been proposed in the literature, including marginal search, exhaustive search, and forward search, their relative performance has only been evaluated through limited simulations due to the lack of an analytical approach to calculating the power of these methods. This article develops a novel statistical approach for power calculation, derives accurate formulas for the power of different model selection strategies, and then uses the formulas to evaluate and compare these strategies in genetic model spaces. In contrast to previous studies, our theoretical framework allows for random genotypes, correlations among test statistics, and a false-positive control based on GWAS practice. After the accuracy of our analytical results is validated through simulations, they are utilized to systematically evaluate and compare the performance of these strategies in a wide class of genetic models. For a specific genetic model, our results clearly reveal how different factors, such as effect size, allele frequency, and interaction, jointly affect the statistical power of each strategy. An example is provided for the application of our approach to empirical research. The statistical approach used in our derivations is general and can be employed to address the model selection problems in other random predictor settings. We have developed an R package *markerSearchPower* to implement our formulas, which can be downloaded from the Comprehensive R Archive Network (CRAN) or http://bioinformatics.med.yale.edu/group/.

## Author Summary

Almost all published genome-wide association studies are based on single-marker analysis. Intuitively, joint consideration of multiple markers should be more informative when multiple genes and their interactions are involved in disease etiology. For example, an exhaustive search among models involving multiple markers and their interactions can identify certain gene–gene interactions that will be missed by single-marker analysis. However, an exhaustive search is difficult, or even impossible, to perform because of the computational requirements. Moreover, searching more models does not necessarily increase statistical power, because there may be an increased chance of finding false positive results when more models are explored. For power comparisons of different model selection methods, the published studies have relied on limited simulations due to the highly computationally intensive nature of such simulation studies. To enable researchers to compare different model search strategies without resorting to extensive simulations, we develop a novel analytical approach to evaluating the statistical power of these methods. Our results offer insights into how different parameters in a genetic model affect the statistical power of a given model selection strategy. We developed an R package to implement our results. This package can be used by researchers to compare and select an effective approach to detecting SNPs.

## Introduction

In genome-wide association studies (GWAS), hundreds of thousands of markers are genotyped to identify genetic variations associated with complex phenotypes of interest. The detection of truly associated markers can be framed as a model selection problem: a group of statistical models are considered to assess how well each model predicts the phenotype, and the selected models are expected to include all or some of the truly associated genetic markers and few, if any, markers not associated with the phenotype. In the literature, three model-selecting procedures have been advocated: marginal search, exhaustive search, and forward search.

Marginal search analyzes markers individually and is the simplest and computationally least expensive among these three search methods. Under certain assumptions, such as no interactions among covariates (or markers in the GWAS context), Fan and Lv [1] proved that the truly associated covariates will be among those having the highest marginal correlations. However, Fan and Lv acknowledged that marginal search may suffer when an important covariate is jointly associated as a group but marginally unassociated as individuals with the response (phenotype). In GWAS, the phenotypes are likely associated with multiple genes, their gene-gene interactions (i.e. epistases), and gene-environment interactions. Therefore, marginal search may not be optimal for the analysis of GWAS data.

In contrast to marginal search, exhaustive search and forward search simultaneously consider multiple markers in the model. Exhaustive search examines all possible models within a given model dimension, and forward search identifies markers in a stepwise fashion. As they consider interactions, they may gain statistical power compared to marginal search [2]–[5]. In practice, exhaustive search bears a much larger computational burden because the number of models that need to be explored is an exponential function of the number of markers jointly considered. For example, if 500,000 markers are genotyped, an exhaustive search of all marker pairs would study around 10^11^ candidate models. This requires significant computational resources, especially when permutations are needed to establish overall significance levels, e.g. for the purpose of appropriately accounting for dependencies among markers. Because of this computational burden, it is difficult or even impossible to assess the power of exhaustive search through simulation studies.

Based on limited simulations and real data analysis, conflicting results exist in the literature on the relative merit of exhaustive search and forward search. Because exhaustive search considers many more models, it may increase the probability that the truly associated markers do not rise to the top as more models involving unrelated markers may outperform the true models simply due to chance. Forward search explores a smaller model space, allowing a less stringent threshold for significance. However, forward search may miss the markers that have a strong interaction effect but weak marginal effect. Through limited simulation studies, Marchini and colleagues [4],[5] concluded that exhaustive search is more powerful in finding truly associated markers in the presence of epistasis. On the contrary, based on the analysis of a real data set for yeast, Storey and colleagues [2],[3] recommended sequential forward search. They reported that exhaustive search suffers from lower power because a substantial increase in the number of models. By analytically demonstrating the conditions under which exhaustive search is better than forward search, and the reverse, our research systematically explains these contradictory results.

It is clear that the optimal model selection strategy depends on the underlying genetic model, which is unknown to researchers. In the most extreme case, if the underlying genetic model has no marginal association, an exhaustive search is the only way to find influential genes. On the other hand, for a model with purely additive genetic effects, marginal or forward search will be the most effective. For the cases between these two extremes, the optimal model selection strategy should achieve a delicate balance between computational efficiency, statistical power, and a low false positive rate. Without the knowledge of underlying models, it is necessary to evaluate the different methods by thoroughly comparing them across a large genetic model space, in which both computationally intensive simulations and limited real data analysis are difficult to fully explore.

In this article, we derive the analytical results for statistical power of marginal search, exhaustive search, and forward search. These formulas can significantly reduce the computational burden in power estimation. To implement the formulas, we developed an R package *markerSearchPower*. We demonstrate through simulations that our results are accurate. Through our results, we can systematically assess different SNP search methods across a large model space and efficiently identify the optimal one. Our derivation approaches are general and can be applied to the model selection procedures in other random predictor settings.

The rest of this article is organized as follows: in the Results section, we present the model set-up, the validation of our analytical results through simulations, and the comparisons among three model selection strategies; in the Discussion section, we summarize the power comparison results and discuss our methodological contributions; and in the Methods section, we outline the derivations of asymptotic distributions and power calculations. The Text S1 available online gives statistical details of proofs and derivations, extended power comparisons, and relevant formulas for the estimates of distribution parameters.

## Results

### Model Setup

A genetic model relates phenotype to genotypes, and this relationship can be rather complex. In general, statistical power depends on the effects of risk alleles, allele frequencies in the population, epistasis, as well as environmental risk factors and their interactions with genetic factors. We focus on a model commonly used in the literature, which offers valuable insights into the relative performance of model selection methods.

Assume that genotype data are available from *p* independent single nucleotide polymorphisms (SNPs). Our results can be generalized to other types of markers. We use *X_i_*_1_, …, *X_ip_*, *i* = 1, …, *n*, to denote the genotypes for the *i*th sampled individual, for SNPs 1, …, *p*, respectively. Let the alleles at the *j*th SNP be *M_j_* and *m_j_* with frequencies *p_j_* and *q_j_* = 1−*p_j_*, respectively. Under the assumption of Hardy-Weinberg equilibrium and additive allelic effects, we use the following coding for this SNP:
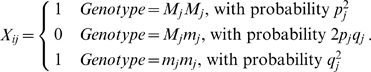
(1)


We focus on the scenario that two of these SNPs, indexed by 1 and 2, are truly associated with a quantitative outcome *Y* through the following genetic model
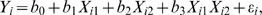
(2)where *ε_i_*∼*N*(0, *σ* ^2^) is independent of the genotypes. The interaction term represents the epistatic effect, and its coefficient *b*_3_ measures the direction and magnitude of this effect.

Based on the observed data, we fit the following models using Ordinary Least Squares (OLS) involving one or two SNPs:
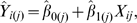
(3)

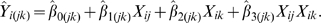
(4)


The subscripts in the above models index the SNP(s) included in these models. Based on models (3) and (4), three model selection methods seek candidate markers according to the corresponding test statistics. In marginal search, we fit simple linear model (3) and compare the *T*-statistics [6] *T_j_* for *j* = 1, …, *p*. A model, and thus its involved SNP, is selected if the corresponding *T*-statistic is among the largest from all tests. In two-dimensional exhaustive search, we fit regression model (4) for all SNP pairs and compare the *F*-statistics [6] *F_jk_* for all *j*<*k* where *j*, *k*∈{1, …, *p*}. The models with the highest values of the *F*-statistics are selected. In forward search, we first conduct a marginal model selection through model (3) and select the *j*th SNP if |*T_j_*| is the largest. With *X_j_*, we then add another SNP *X_k_* (*k*≠*j*) for different SNPs, and choose models in format (4) which generate the highest *F*-statistics.

Two criteria are adopted to decide if the chosen models are correct. On one hand, we could be rather stringent and call a model correct only if it matches the true underlying genetic model. This is consistent with the concept of “joint significance” in Storey et al. [2]. On the other hand, we could be more generous and call a model correct if it contains at least one of the truly associated markers. This is consistent with the null hypothesis used in some published simulation studies [4],[5]. Accordingly, we consider two definitions of power for a model selection procedure:

- the probability of identifying exactly the true model (in marginal search, it is the probability of detecting both true SNPs);
- the probability of detecting at least one of the true SNPs.

Under power definition (A), the null model is any model other than the true genetic model; under power definition (B), the null model is any model containing neither true SNP.

### Comparison between Analytical and Simulation Results

We evaluated the accuracy of the asymptotic results derived in the Methods section by comparing the analytical results with those from simulations. To estimate power through simulation studies, we generated 1,000 data sets with *n* subjects and *p* candidate SNPs assuming Hardy-Weinberg equilibrium, as indicated in (1). The quantitative trait values were generated through true model (2) involving two true SNPs. We then used marginal search, exhaustive search, and forward search to identify SNPs associated with the trait. Under power definition (A), the target model(s) were the true model (or models with one true SNP in marginal search), and the other models were considered null models. Under power definition (B), the target models were those containing at least one true SNP, and the rest were considered null models. The empirical power estimated from these simulations was the proportion of that datasets that we were able to successfully find the target model(s) through model selection procedures, under the control of a pre-specified number (*R*) of falsely discovered null models. Such control offers a fair comparison of power among the three model selection methods and is numerically equal to the detection probability (DP) control [7], which is the probability of including a “correct model” when selecting *R* (or *R*+1 in marginal search under power definition (A)) of the most significant models.

In the first set-up for model (2), we considered *n* = 100 subjects, *p* = 300 SNPs, genetic effects *b*_1_ = *b*_2_ = 0.1, *b*_3_ = 2.4, allele frequency of each SNP *q_j_* = 0.3, *j* = 1, …, *p*, and variance *σ* ^2^ = 3. Table 1 summarizes the calculated power and the simulated power under definitions (A) and (B). The second set-up is the same as the first except *b*_3_ = 1.4. For this set-up, Table 2 shows the results under definitions (A) and (B). The two values of *b*_3_ represent large and small interaction terms with which the simulation generated a broad spectrum of power values. In both set-ups, the analytical power is very close to the empirical power based on simulations.

**Table 1:** The probability of detecting the exact true model (or both true SNPs in marginal search) under power definition A, and the probability of detecting at least one of the true SNPs under power definition B, with the false discovery number *R* varying. *b*_1_ = *b*_2_ = 0.1, *b*_3_ = 2.4.

Category	Strategy	Source	*R* = 1	*R* = 5	*R* = 10	*R* = 15	*R* = 20	*R* = 30
**Definition A**	Marginal search	simulation	0.268	0.556	0.683	0.754	0.790	0.851
		calculation	0.279	0.552	0.673	0.738	0.781	0.836
	Exhaustive search	simulation	0.987	0.998	1.000	1.000	1.000	1.000
		calculation	0.978	0.995	0.998	0.998	0.998	1.000
	Forward search	simulation	0.780	0.788	0.789	0.789	0.789	0.789
		calculation	0.795	0.800	0.800	0.800	0.801	0.801
**Definition B**	Marginal search	simulation	0.790	0.950	0.980	0.985	0.993	0.995
		calculation	0.806	0.958	0.982	0.990	0.993	0.997
	Exhaustive search	simulation	0.993	0.999	1.000	1.000	1.000	1.000
		calculation	0.985	0.999	0.999	1.000	1.000	1.000
	Forward search	simulation	0.843	0.910	0.944	0.961	0.974	0.986
		calculation	0.828	0.906	0.938	0.952	0.966	0.983

**Table 2:** The probability of detecting the exact true model (or both true SNPs in marginal search) under power definition A, and the probability of detecting at least one of the true SNPs under power definition B, with the false discovery number *R* varying. *b*_1_ = *b*_2_ = 0.1, *b*_3_ = 1.4.

Category	Strategy	Source	*R* = 1	*R* = 5	*R* = 10	*R* = 15	*R* = 20	*R* = 30
**Definition A**	Marginal search	simulation	0.055	0.180	0.291	0.359	0.425	0.512
		calculation	0.053	0.179	0.289	0.355	0.424	0.515
	Exhaustive search	simulation	0.394	0.586	0.667	0.706	0.715	0.753
		calculation	0.399	0.567	0.638	0.681	0.707	0.728
	Forward search	simulation	0.242	0.308	0.331	0.340	0.343	0.348
		calculation	0.238	0.308	0.331	0.342	0.349	0.354
**Definition B**	Marginal search	simulation	0.394	0.695	0.802	0.869	0.899	0.935
		calculation	0.406	0.698	0.807	0.862	0.894	0.932
	Exhaustive search	simulation	0.533	0.757	0.823	0.850	0.880	0.910
		calculation	0.569	0.738	0.809	0.848	0.874	0.906
	Forward search	simulation	0.422	0.561	0.654	0.731	0.769	0.841
		calculation	0.433	0.554	0.647	0.711	0.758	0.821

We chose these two set-ups in which the power was reasonably large to approximate most practical settings. The chosen value of *p* is much smaller than that in GWAS (in the 100,000's) for the feasibility of simulation. As discussed in the Methods section, the asymptotic results are derived by assuming a large *p*. Therefore, we expect better approximations if *p* has a value similar to those in a real GWAS.

### Power Comparisons of Model Selection Methods

The simulation results shown in Table 1 and Table 2 demonstrate that our analytical results provide good approximations to the true power, which is the basis for comparing the performance of these model search methods in a practical GWAS. We now consider a more realistic setting with a sample size of 1000 individuals (*n*) and a total of 300,000 SNPs (*p*). We assumed a genetic model of form (2) with *σ* ^2^ = 3 and varied the values of *b*_1_ = *b*_2_ as well as that of *b*_3_ from −1 to 1 by a step size of 0.1. To simplify the discussion, we assumed all SNPs had the same allele frequency of *q_j_* = 0.3, *j* = 1, …, *p*. Note that this setting can be changed without affecting the qualitative nature of the comparison results.

**Figure 1:**
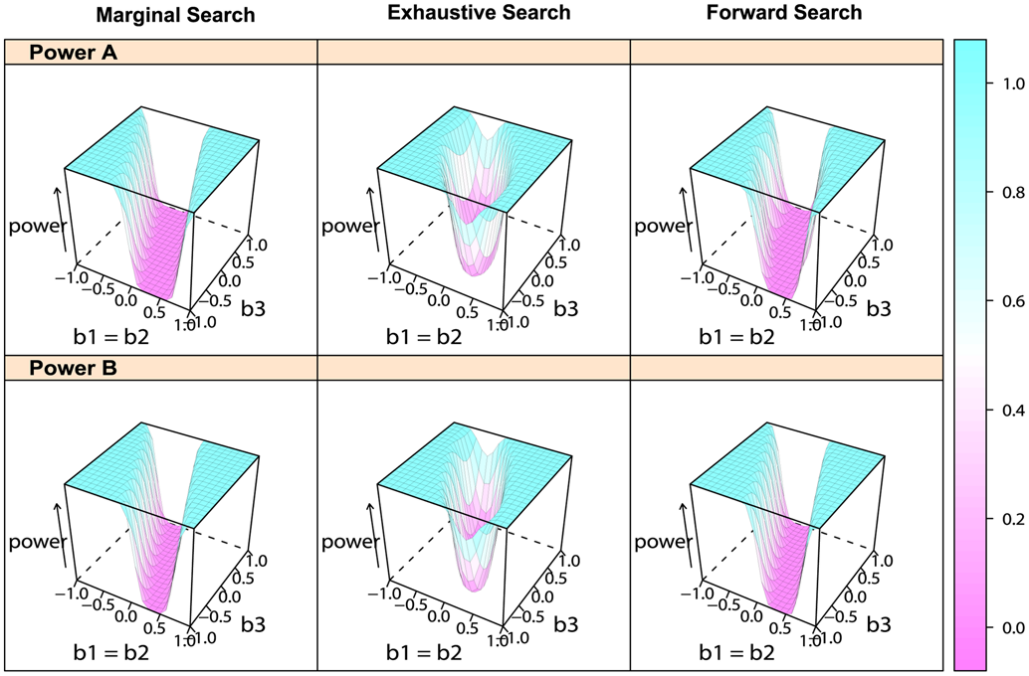
3D plots of statistical power over genetic model space. The results of power for the three model selection methods: marginal search in the left column, exhaustive search in the middle column and forward search in the right column. Two definitions of power (A) for detecting the true model or both true SNPs in marginal search in row 1, and (B) for detecting either true SNP in row 2 are considered. We consider genetic models with the main effects *b*_1_ = *b*_2_ varying from −1 to 1 and the epistatic effect *b*_3_ varying from −1 to 1. The allele frequency *q_j_* = 0.3, *j* = 1, …, *p*, and the false discovery number *R* is set to be 10.

Figure 1 gives the 3D plots of statistical power over the genetic model space for different model selection methods (in columns) under two power definitions (A) and (B) (in rows), when controlling the number of false discoveries to be *R* = 10. These figures illustrate that marginal search and forward search cannot detect the marginal association of the influential SNP 1 or 2 in a certain region of the model space, while exhaustive search can. This portion of the model space is represented by the region where the power of marginal search and that of forward search are very close to 0, no matter how large the genetic effect is. According to formulas (8) and (16) in the Methods section, the marginally non-detectable region for SNP 1, where *b*_1_+*b*_3_(*p*_2_−*q*_2_) = 0, depends on the additive genetic effect *b*_1_, epistatic effect *b*_3_, and the allele frequency *p*_2_ of SNP 2. The non-detectable region for SNP 2 is analogous by symmetry. In exhaustive search, such region does not exist, as indicated by formula (12). So, exhaustive search can better identify the signals when they are counterbalanced.

**Figure 2:**
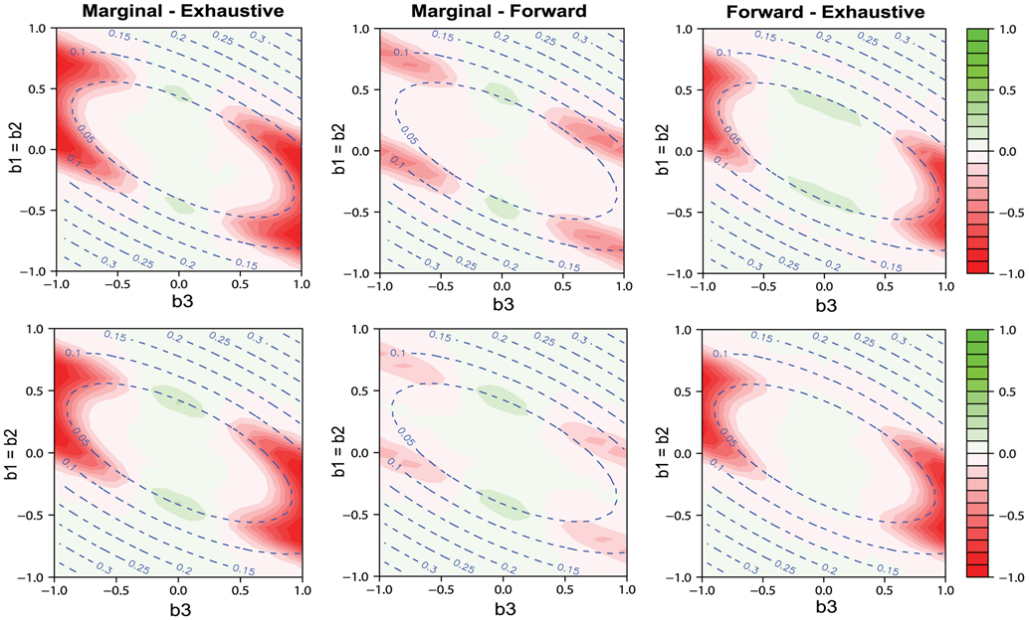
Comparisons among model selection power for detecting the true model or both true SNPs in marginal search over genetic model space. The power differences between marginal search and exhaustive search in the left column, between marginal search and forward search in the middle column, and between forward search and exhaustive search in the right column. Green areas indicate positive values of difference, and red areas indicate negative values of difference. We consider genetic models with the main effects *b*_1_ = *b*_2_ varying from −1 to 1 and the epistatic effect *b*_3_ varying from −1 to 1. The allele frequency *q_j_* = 0.3, *j* = 1, …, *p*, and the false discovery number *R* is set to be 1 in row 1 and 10 in row 2.

**Figure 3:**
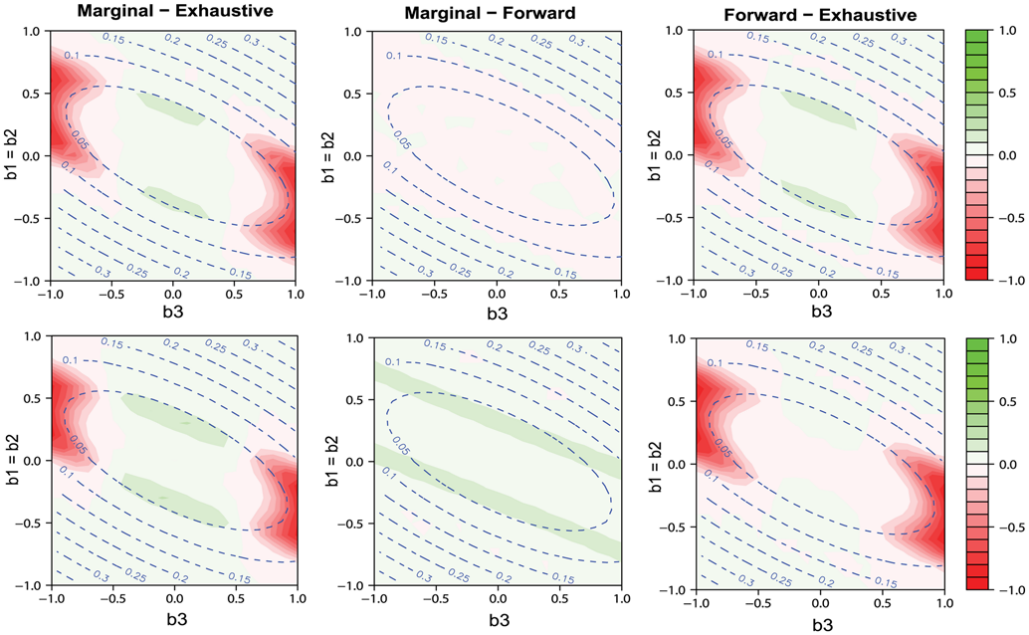
Comparisons among model selection power for detecting either true SNP over genetic model space. The power differences between marginal search and exhaustive search in the left column, between marginal search and forward search in the middle column, and between forward search and exhaustive search in the right column. Green areas indicate positive values of difference, and red areas indicate negative values of difference. We consider genetic models with the main effects *b*_1_ = *b*_2_ varying from −1 to 1 and the epistatic effect *b*_3_ varying from −1 to 1. The allele frequency *q_j_* = 0.3, *j* = 1, …, *p*, and the false discovery number *R* is set to be 1 in row 1 and 10 in row 2.

In order to better visualize the difference of model selection methods, we show the power differences between different methods. The left, middle, and right columns of Figure 2 and Figure 3 present the power difference between marginal search and exhaustive search, between marginal search and forward search, and between forward search and exhaustive search, respectively. For a specific comparison, the red areas represent negative values, indicating the former method has lower power, and the green areas represent positive values, indicating the former method has higher power. The dashed contours in these plots represent the heritability of the genetic model, i.e., the proportion of the total variation due to genetic effects, which is defined as
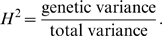
Under our model set-up,
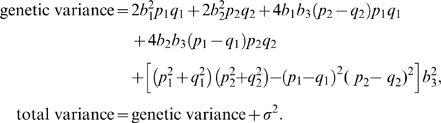
In each plot, there are two areas in which the difference of power is close to 0. First, in the central area where the signal is weak (small *H* ^2^), all model selection procedures have low power and tend to fail to pick up the true SNPs. Second, in the edge areas where the signals are strong, all model selection procedures have similarly good power. The light colored areas represent these two special situations in which there is little difference in power among model selection methods.

To compare marginal search and exhaustive search, the left columns of Figure 2 and Figure 3 exhibit the power difference under power definitions (A) and (B), respectively. Exhaustive search has significant advantage in the red areas where the interaction effect *b*_3_ is large or *b*_1_+*b*_3_(*p*_2_−*q*_2_) is small. Such advantage is more pronounced under power definition (A) than under power definition (B). Marginal search performs better in the green areas where *b*_3_ is small and *b*_1_ and *b*_2_ are both moderate. There are two reasons for the better performance of marginal search. First, with a small interaction term *b*_3_ in these green areas, marginal search well detects the signals when the two-marker genetic effects are projected onto a marginal space through the simple regression of form (3). At the same time, with moderate *b*_1_ and *b*_2_, the power for these two methods is not close to 0 or 1, so that they are distinguishable. Second, marginal search considers fewer models so that the desired models are more likely to be found from the models with the best fit.

Under different power definitions, the performance of forward search relative to that of marginal search can change. Capable of including interaction terms, forward search has an advantage over marginal search in finding the full correct model under power definition (A), as shown by the red areas in the middle column of Figure 2. Based on the analytical formulas in the Methods section, there is a positive correlation between the test statistics in the first and second steps of forward search. Therefore, if one of the associated SNPs can be picked up in the first step, the contribution of the epistatic term makes forward search more powerful to identify the second correct SNP. Under power definition (B), the middle column of Figure 3 shows that marginal search always has similar or slightly better power than forward search, because forward search is less likely than marginal search to pick up a true SNP if an incorrect SNP is chosen first. The power of forward search will not improve greatly even if the number of false discoveries *R* increases.

As shown in the right column of Figure 2, exhaustive search under power definition (A) always has a similar or higher power to detect the true model when compared to forward search. Although forward search can also detect the interaction terms through joint analysis, its ability to capture the interaction terms is restricted, especially when marginal effect is small in the deep red areas of *b*_1_+*b*_3_(*p*_2_−*q*_2_)≈0. Under power definition (B), forward search is more powerful than exhaustive search when *R*, the number of controlled false discoveries, is small, but is less powerful when *R* is large. With small *R* (e.g. *R* = 1), forward search benefits from considering fewer models and is better than exhaustive search in the green areas of Figure 3. This benefit is reduced for larger *R* and will eventually be dominated by the advantage of exhaustive search. Since the first step of forward search is essentially a marginal search, the advantage of exhaustive search over marginal search also applies to forward search. This is reflected in the right columns of Figure 2 and Figure 3, where the red areas are similar to those in the left columns.

As reflected by the change of red/green areas between the first and the second rows in both Figure 2 and Figure 3, if we raise the number of allowed false discoveries *R*, the power of marginal search will increase the most, followed by the power of exhaustive search, and then the power of forward search. With the same increase in *R*, marginal search includes a much higher proportion of the models with true SNPs than exhaustive search. For forward search, the increase of power is smaller because it is more difficult to identify a correct SNP in the second step when an incorrect SNP is more likely to be selected in the first step.

We also explored additional model set-ups in Text S1 Section 3 with *n* = 100, *p* = 1000, *R* = 1, 5, and 10, *q_j_* = 0.3 and 0.5, *j* = 1, …, *p*, and *σ* ^2^ = 3. The values of the genetic effects *b*_1_ = *b*_2_ and *b*_3_ varied from −2 to 2 by a step size of 0.2. When *q_j_* = 0.5, the graphs are symmetric about *b*_1_ = *b*_2_ = 0 and *b*_3_ = 0. In general, the patterns are similar to those shown in Figure 2 and Figure 3.

### An Example of Power Comparisons Motivated from Real GWAS

In the following we provide an example to show how to apply our approach to calculating and comparing the power of model selection methods in empirical analysis. Because there are no consistently replicated interaction effects from real studies, we constructed hypothetical interaction models based on real data so that the marginal associations between traits and markers were matched, while allowing the interaction term to vary. Specifically, we calculated power based on a set of genetic models derived from a genome-wide association study of adult height by Weedon et al. [8]. Based on the reported 20 loci that putatively influence adult height, we set up a two-marker genetic model composed of SNPs rs11107116 and rs10906982, each of which showed moderate marginal effect. According to the Supplementary Table 4 in the original publication, the estimated marginal effects of rs11107116 and rs10906982 are respectively 0.045s.d. and 0.046s.d. with a sample standard deviation (s.d.) of height of 6.82 cm. Assuming different levels of interaction between the two SNPs (quantified by *b*_3_), we estimated the parameters *b*_1_, *b*_2_, and *σ* ^2^ using model (2) so that the marginal effects matched the observed values. The Methods section gives the details of how these parameters were estimated. We used the set-up of Weedon's study: sample size *n* = 16,482, number of candidate SNPs *p* = 402,951, and the frequencies of the height-increasing allele for rs11107116 and rs10906982 *p*_1_ = 0.77 and *p*_2_ = 0.48, respectively.

**Figure 4:**
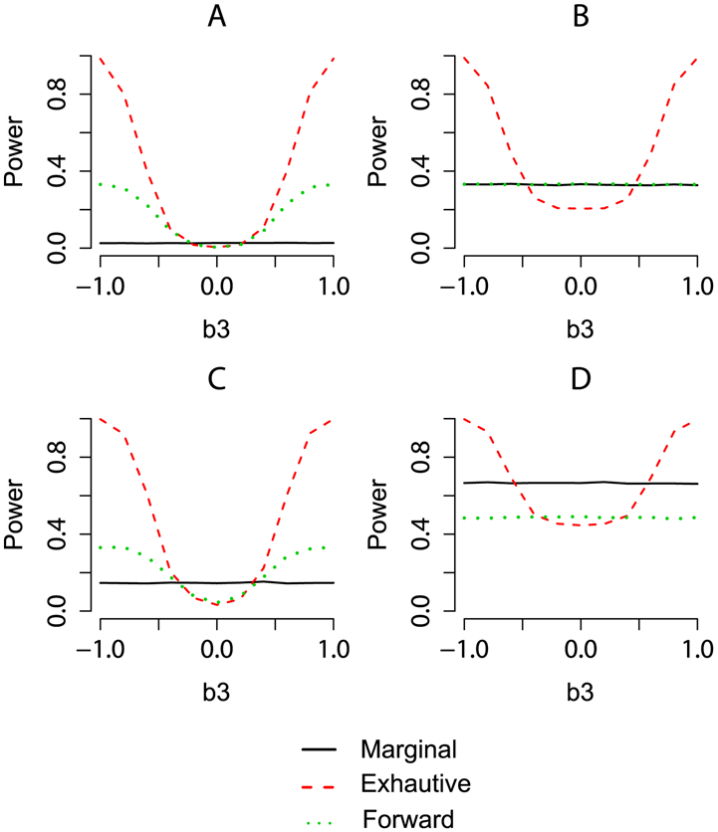
Plots of model selection power with given observed marginal effects. Power comparisons of three model selection procedures over a sequence of epistatic effect *b*_3_: marginal search by black solid curve, exhaustive search by red dashed curve, and forward search by green dotted curve. We assume the true SNPs to be rs11107116 and rs10906982, which influence adult height with their marginal effects set to be the same as those observed in Weedon et al. 2008. Graphs A with *R* = 1 and C with *R* = 20 indicate the power of finding both SNPs; graphs B with *R* = 1 and D with *R* = 20 indicate the power of finding at least one of the two SNPs.

Figure 4 shows the comparisons among the power of the three model selection methods over different values of *b*_3_. For the detection of both SNPs, graphs A (*R* = 1) and C (*R* = 20) indicate that if the magnitude of epistasis *b*_3_ is large, exhaustive search (red dashed curve) has significant advantage over forward search (green dotted curve), which is better than marginal search (black solid curve). If *b_3_* is small, marginal search has higher power than the other two. For the detection of at least one of the two SNPs, graphs B (*R* = 1) and D (*R* = 20) indicate that marginal search is similar or better than forward search; both methods are not affected by the variation of *b*_3_. The relative performance of exhaustive search strongly depends on the magnitude of epistasis. Comparing graphs B (*R* = 1) and D (*R* = 20), it is clear that marginal search is superior over a larger region when a larger false discovery number *R* is tolerated.

With *R* = 20, graphs C and D indicate that exhaustive search is better than marginal search to find both or at least one of the SNPs when the magnitude of *b*_3_>0.3 or 0.6, respectively. We studied the statistical significance of the interaction terms with the simulated data (1,000 runs) when *b*_3_ equals these two cutoffs. When *b*_3_ = 0.3, 11.4% of the simulations had the Bonferroni p-values (adjusted by the number of all possible pairs of the 20 found loci) that exceeded the significant threshold at 0.05. Therefore, a small epistatic effect, rarely showing significance from the observed data, can still make an exhaustive search more powerful than a marginal search under power definition (A). Under power definition (B), when *b*_3_ = 0.6, 87.3% of the Bonferroni adjusted p-values were significant. That is, to make exhaustive search more powerful than marginal search for finding either SNP, a true epistatic effect needs to be large enough to often identify a statistically significant interaction.

This example demonstrates that the value of the interaction term and the number of false discoveries affect the relative performance of model selection methods, which can be one of the reasons for the conflicting results about the power of model selection methods in the existing literature [2],[4]. Therefore, the suspected values of parameters such as epistatic effects can affect the researchers' choice of model selection methods.

## Discussion

In this article, we have derived rigorous analytical results for the statistical power of three common model selection methods, and applied these results to compare the methods' performance for GWAS data. These results not only make the computationally expensive simulations unnecessary, but also systematically reveal how different genetic model parameters affect the power.

The comparison results among the three model selection methods illustrate the trade-off between searching the full model space and a reduced space. In one extreme, exhaustive search explores the full 2-dimensional space covering all possible epistatic effects, but it may reduce the probability that the true model(s) ranks among the top models because many more models are considered. In the other extreme, marginal search casts the true 2-dimensional model onto a 1-dimensional space without considering epistasis at all. However, we have a better chance to find more true positives when the marginal association is retained in the 1-dimenisonal space, because fewer models are examined and the false positive control appears comparatively liberal. Between these two extremes, forward search first considers marginal projection, and then partially searches the 2-dimensional space via residual projection given the chosen predictor in the first step. Thus, forward search has the partial benefit of joint analysis which considers epistatic effects conditionally. The stringency of its false positive control exists between those of exhaustive search and of marginal search.

The relative performance of these model selection methods also depends on the definition of power. Based on definition (A), exhaustive search performs the best in finding the true underlying genetic model in most of the model space considered. Under power definition (B), marginal search is a good choice: it is not much worse than exhaustive search for a large proportion of the model space, and it is always better than the classic forward search through which only one SNP is picked up in the first step. For most geneticists, finding at least one of the truly associated SNPs under power definition (B) is a primary concern, especially in the first stage of GWAS. Because we do not have prior information about the true genetic model in the beginning, marginal search, which is easy to compute, is a good start in the first stage of GWAS to find one or some of the main genetic effects. In the later stage(s), if the promising SNP candidates are limited, exhaustive search can be applied with less demanding computation, especially when epistasis among loci is of interest. Our conclusions based on the analytical studies justify this multi-stage strategy in GWAS.

### Difference between Our Methods and Traditional Power Calculation and Simulations

Our power calculation for model selection strategies is different from a traditional power calculation for multiple regression models [9]. The traditional approach is to calculate the probability of accepting a specific multiple-regression model and rejecting the null hypothesis that the response and the covariates have no association, when controlling the type I error rate. This power calculation focuses on models instead of model selection methods, as it does not address any procedure of model selection. In contrast, our analytical approach is to calculate the probability that a model selection method can pick up the models that contain the true covariates (true SNPs in GWAS).

Our analytical approach leads to new insights into model selection methods than simulations and limited real data analysis. Furthermore, our approach addresses a critical limitation of prior studies [4],[5] that do not distinguish the models with all correct predictors from those with only a subset of the correct predictors. In those studies, the null distribution assumes the test statistic is from a model without any of the true predictors, and the alternative distribution assumes the statistic is from any model containing at least one true predictor (or, when considering the power for finding both true loci, the models with either true locus are ignored from the null distribution). This is a common problem of traditional multiple testing for model selection method, as pointed out by Storey et al. [2], who stated that “there is no statistically rigorous method to test for joint linkage, which exists only if both loci have nonzero terms in the full model.” To address this issue, all involved models (including true, partially true, and wrong models) are considered and ranked by how well they fit the observed data. Our power calculation distinguishes the case that model selection procedures find the true model based on power definition (A) from the case that the procedures find a partially true model based on definition (B). We have derived the null and alternative distributions for each case, and thus provide the basis for model performance comparisons.

To compare the power of model selection methods, our approach explicitly considers the correlation structures among the test statistics for the null and alternative hypotheses, which achieves more accurate assessment of model selection methods than Bonferroni-corrected type I error control that is commonly used in the literature [4],[5]. Bonferroni-based control is usually a conservative control when the test statistics are dependent on each other. As illustrated by both simulations (results not shown) and the theoretical derivations in the Methods section, the considered models and their test statistics usually exhibit complex correlation structures. Therefore Bonferroni-based control is not optimal as it only considers the number of models evaluated (that is, the number of hypothesis tests) and ignores correlation structures generated by different search strategies. The adequacy of our approach has been demonstrated through a good agreement between the analytical and the simulation results shown in Table 1 and Table 2. Furthermore, our study of correlation structures improves the understanding of the mechanism of different search strategies discovering genetic signals. For example, in forward search, the failure of the first stage is likely to cause the failure of the second stage even if there is a large epistatic effect, because the test statistics for the true predictors are positively correlated between the two stages.

### Control Related to Type I Error Rate and False Discovery Proportion

To obtain the significance threshold, we control the number of false discoveries at *R* depending on how the power is defined. This control is practically meaningful and equals to the detection probability (DP) control [7] as discussed in the Results section. Furthermore, controlling the number of false discoveries is related to controlling the type I error rate. Since the type I error rate is defined as the probability of rejecting a hypothesis given it is a true null, with the definition of null models corresponding to the power definition (A) or (B), the estimation of component-wise type I error rate could be considered as
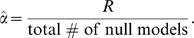



The model selection problem is also a large-scale simultaneous hypothesis testing problem. A widely applied significance control criterion in this scenario is the false discovery rate (FDR) [10]. The false discovery number control in our study is also related to the control of the false discovery proportion (FDP), which is an estimate of FDR. Under power definition (A)
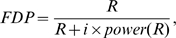
where *power*(*R*) denotes the power calculated based on the number of selected null models *R*, and *i* indicates the number of correct models: *i* = 2 for marginal search, and *i* = 1 for exhaustive search and forward search.

### On the Derivation of Asymptotic Distributions

Through the simulations in the Results section, our derivation of asymptotic distributions is shown to be accurate for moderately small genetic effects when the sample size *n* = 100. Since the asymptotic derivation assumes large sample size, the power calculation results should provide accurate approximations for reasonably smaller genetic effects in GWAS which have a much larger number of observations in general. The asymptotic derivation has several benefits. First, we can derive the results for the models with random predictors. Because genotypes are randomly observed in genetic studies, it is necessary to consider such models. Traditional methods for deriving the non-central *F* distributions for the test statistics are based on fixed predictors [7],[11],[12]. As functions of predictor variables, these non-central parameters are not statistically consistent when genotypes are random. Although one may integrate the power over all possible configurations of markers [13], it is very cumbersome unless *n* is small. Our method, based on asymptotic theorems, provides a satisfactory solution for models with random predictors. Our novel approach presented here can be applied to derive the distributions of such models' test statistics. Second, the derived asymptotic multivariate normal distributions for theoretical null and alternative hypotheses allow us to incorporate complex correlations among the test statistics into power calculation based on population parameters. For a given GWAS data set, the correlations presented in the data may also be addressed by empirical estimation of the null hypothesis [14],[15]. Third, the ideas behind the asymptotic derivation can be applied to study the distributions for hypothesis testing and power calculation in general as long as the statistics have certain functions of random variables.

### On Simplifying Assumptions

We have assumed that the markers are independent in this paper. There may be linkage disequilibrium (LD) among SNPs. However, LD in general is weak among tagging SNPs [16]–[18]. Furthermore, simulations based on real GWAS data (results not shown) indicate that even in the presence of LD, our analytical results are quite accurate when more false positives are acceptable, i.e. a large *R* value. In addition, the analytical power approximations are more accurate for power definition (B) than for definition (A). In general, when the dependency among true SNPs and the ensemble of unrelated SNPs is weak or moderate, our power calculation provides acceptable approximations.

In reality, the underlying true model could be more complicated than model (2) with more related SNPs and interactions. Our analytical results of power calculation can be extended through the approaches similar to the one we developed here. Although the genetic models studied are simple, our results provide insights into the relative performance of different model selection procedures.

## Methods

### Asymptotic Distribution Results

To calculate the power of model selection procedures shown in the Results section, we first derive general results on the asymptotic distributions. Let **Z** *_i_* = (*Z_i_*_1_, …, *Z_is_*), *i* = 1, …, *n*, be *n* independent and identically distributed (iid) random vectors of dimension *s*. Assume the mean vector is **θ** = *E*(**Z** *_i_*) = (*θ*_1_, …, *θ_s_*) with *θ_j_* = *E*(*Z_ij_*) and the variance-covariance matrix is Σ = *Cov*(**Z** *_i_*) with (Σ)*_jk_* = *Cov*(*Z_ij_*, *Z_ik_*), *j*, *k* = 1, …, *s*. Let 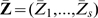, where 
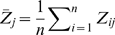
. Considering a real valued function 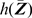 of 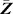, if 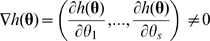, we have
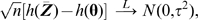
(5)where 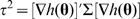 and 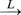 denotes the convergence in law [19].

We extend the above result in two ways to suit our needs of deriving the distribution of test statistics that are examined in model selection procedures (the proofs are given in Text S1 Section 1.1). First, we consider two real valued functions 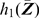 and 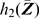 of the same sample mean 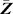. If 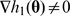 and 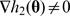, we have the convergence in probability that:
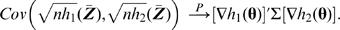
(6)


Secondly, if 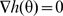, the asymptotic distribution of 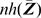 is:
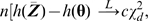
(7)where if 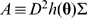, with 
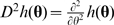
 being the Hessian matrix of *h*(**θ**), we have

1. *c* = 1/2, *d* = *rank*[*A*], if *A* is idempotent;
2. *c*≈*trace*(*A* ^2^)/2*trace*(*A*), *d*≈*trace*(*A*)^2^/*trace*(*A* ^2^), if *A* is not idempotent.

### Power Calculations

With the results above, we derive the relevant distributions of *T*- and *F*-statistics associated with three types of regression models, which will be used for calculating the power of model selection methods. Specifically, *F*_12_ is the *F* statistics for the correct model in which both SNPs are true. *T_i_* and *F_ij_*, *i* = 1, 2, *j* = 3, …, *p*, are test statistics for “half” correct models in which only one SNP is truly associated. *T_j_* and *F_lk_*, 3≤*l*<*k*≤*p*, are the statistics for incorrect models in which neither SNP in the models is associated with the phenotype. Complex correlations exist among the models even with the assumption of independence among SNPs. The correlations come from two sources. First, since the quantitative trait is associated with both SNPs 1 and 2, the fitted regression models containing either of these SNPs have correlated test statistics. Second, models sharing a common SNP (no matter it is true or wrong) also have correlated test statistics. To allow correlations, we therefore explore the marginal and the joint distributions of various test statistics for different models, and then derive how likely a “half” correct model would stand out from incorrect models, as well as how likely a correct model would outperform “half” correct models or incorrect models.

### Marginal Search

#### Statistics and asymptotic distributions

To calculate the power of marginal search, we need to obtain the distributions of the involved test statistics. We first derive the *T*-statistic for the two true SNPs in the marginal model. In the simple regression model involving the first true SNP (SNP 1), i.e. 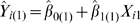, the corresponding *T*-statistic has the following asymptotic distribution (see Text S1 Section 2.1 for proof):
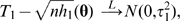
where
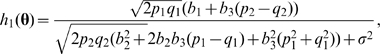
(8)and the formula of 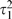 (a constant of *n*) is given in Text S1 Section 4.1. For the marginal model of the second SNP (SNP 2), the asymptotic distribution of *T*_2_ is gotten by symmetry between indices 1 and 2.

Based on the asymptotic mean of *T*_1_ derived above, we can quantify the influence of genetic parameters of SNP 2 and epistasis on the power of marginal search to pick up SNP 1. As for some genetically interesting observations, when there is no epistatic effect (i.e. *b*_3_ = 0), we have 
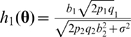
, which means the magnitude of marginal association of *X*_1_ and thus the power of marginal search to find *X*_1_ are decreasing functions of the main effect of *X*_2_, the minor allele frequency (MAF) of *X*_2_, and the random error variance *σ* ^2^, with the decreasing rate specifically given by 
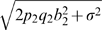
. When epistasis exists (i.e. *b*_3_≠0) but *b*_1_ = 0, *h*_1_(**θ**) reflects the marginally projected signal of epistasis, which is still a decreasing function of the MAF of *X*_2_. The influence of *b*_2_ depends on the allele frequencies *p*_1_ and *q*_1_. On the other hand, if *b*_1_≠0, it is possible that *b*_1_+*b*_3_(*p*_2_−*q*_2_) = 0 when the main effect *b*_1_ and interaction effects *b*_3_ have opposite directions (assuming *q*_2_ is the MAF). With such epistatic pattern, marginal detection surely fails to detect the true genetic variants no matter how strong the true genetic effects are.

Now we derive the joint distribution of *T*_1_ and *T*_2_. Since *Y* is a function of both *X*_1_ and *X*_2_ in the underlying true model (2), *T*_1_ and *T*_2_ are correlated even when *X*_1_ and *X*_2_ are independent and do not interact, i.e. *b*_3_ = 0. The correlation between *T*_1_ and *T*_2_ can be substantial in certain genetic models. The asymptotic joint distribution of (*T*_1_, *T*_2_)′ is
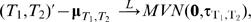
(9)where 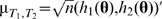, 
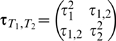
, 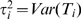, *i* = 1, 2, and τ_1,2_ = *Cov*(*T*_1_, *T*_2_). The covariance τ_1,2_ is gotten based on the result in (6), and its formula (as a constant of *n*) is given in Text S1 Section 4.1.

Let *T_j_*, *j* = 3, …, *p*, be the *T*-statistic from model (3) for a wrong SNP *j*, according to the asymptotic result in (5),
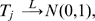
(10)which holds regardless of the allele frequencies and the underlying true genetic model. The proof for *T*_3_ as an example is provided in Text S1 Section 2.2. It can be shown that *T_j_* is also independent of *T*_1_ and *T*_2_ according to the result in (6). Under the assumption of fixed design matrix, 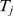 has a *T* distribution with *n*−2 degrees of freedom based on a traditional linear model analysis [6],[12]. This null distribution is still asymptotically valid for random predictors since the *T* distribution converges to the standard normal as *n*→∞.

#### Power of marginal search procedure

Based on the above results for the distributions of *T*-statistics, we first calculate the power of marginal search under power definition (A). If the marginal search is allowed to contain *R* wrong SNPs, i.e. the number of false discoveries is controlled by *R*, the power of identifying both true SNPs is just the probability that both |*T*_1_| and |*T*_2_| are greater than the *R*th largest value in the set {*T_j_*, *j*≥3}:
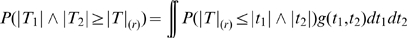
where 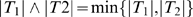, *r* = *p*−2−*R*+1, |*T*|_(*r*)_ is the *r*th smallest (or the *R*th largest) order statistics of |*T_j_*|, *j* = 3, …, *p*, and *g*(*t*_1_, *t*_2_) is the joint probability density function (PDF) of (*T*_1_, *T*_2_)′ given in (9). Let Φ(·) be the cumulative distribution function (CDF) of *N*(0, 1), then
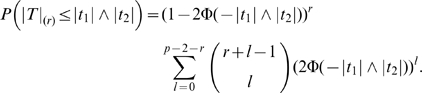
To get the power of marginal search under definition (B) that either SNP 1 or SNP 2 is selected, we calculate the probability that either |*T*_1_| or |*T*_2_| is larger than the random cutoff point: *P*(|*T*_1_|∨|*T*_2_|≥|*T*|_(*r*)_), where |*T*_1_|∨|*T*_2_| = max{|*T*_1_|, |*T*_2_|}.

### Exhaustive Search

#### Statistics and asymptotic distributions

The distributions of the relevant test statistics are derived first for calculating the power of exhaustive search. We first get the joint distribution of the test statistics involving true SNPs 1 and 2: *T*_1_, *T*_2_, and *F*_12_. Define 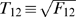. Based on the asymptotic distribution result in (5) (see Text S1 Section 2.3 for details of derivation), we have
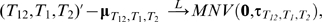
(11)where 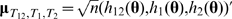 and 
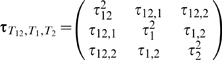
.

The formula of *h*_1_(**θ**) is given in (8), and
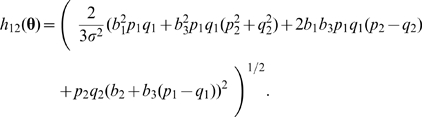
(12)The formulas of 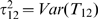 and τ_12,*i*_ = *Cov*(*T*_12_, *T_i_*), *i* = 1, 2, are independent of *n* and are given in Text S1 Section 4.1.

We then derive the *F*-statistics for the incorrect models in form (4) to fit *Y* with *X_j_* and *X_k_*, 3≤*j*<*k*≤*p*. Following the result in (7), *F_jk_* has a common marginal asymptotic distribution:
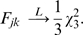
(13)With *F*_34_ as an example, the detailed proof is given in Text S1 Section 2.4.

Based on the traditional power calculation for regression models, the null model is the incorrect model with neither SNP associated with the phenotype. When the design matrix is fixed, the null distribution of *F_jk_* is an *F* distribution with degrees of freedom (3, *n*−4) [12]. Result (13) indicates the *F* distribution for null is also valid when the genotypes are treated as random variables, because *F*(3, *n*−4) converges to 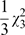 when *n* is large.

In order to calculate the power of model selection methods, we need to address the correlation structures among involved statistics. The statistics are correlated when two epistatic models in form (4) share a common SNP. Also, *F*-statistics involving *X*_1_ and those involving *X*_2_ are correlated because the true underlying model includes both SNPs. Consequently, the elements in the set {*F*_12_, *F_ij_*, *i* = 1, 2, *j* = 3,…,*p*} are all correlated with each other. To capture the important dependency, we decompose *F*-statistics as follows:
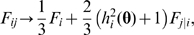
(14)when *i* = 1, *h*_1_(**θ**) is given by equation (8). The detailed proof for decomposing *F*_13_ as an example is shown in Text S1 Section 1.2. Through this decomposition, the correlation between *F_ij_* and *F_ik_*, can be explained by *F_i_* while we treat *F_j_*_|*i*_ and *F_k_*_|*i*_ to be independent. Furthermore, with the result (14) we can use the joint distribution (11) to capture the correlation between *F*_12_ and *F_ij_*.

Based on the asymptotic distribution in (7), we have
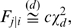
(15)where *i* = 1, 2, *j* = 3, …, *p*, *c* = *v*/2*e*, and *d* = 2*e* ^2^/*v*, with *E*(*F_j_*_|*i*_)→*e* and *Var*(*F_j_*_|*i*_)→*v*. Text S1 Section 2.5 shows the detailed proof for *F*_3|1_. The formulas of *e* and *v* are given in Text S1 Section 4.2. Based on our numerical studies (results not shown), *c* is close to 1/2 and *d* is close to 2 in a large proportion of the parameter space of {*q_i_*, *q_j_*, **b**, *σ* ^2^} (e.g. when allele frequencies *q_i_* and *q_j_* do not converge to 0 or 1, genetic effect **b** = (*b*_1_, *b*_2_, *b*_3_)′ and random error variance *σ* ^2^ are not too large). When *c* = 1/2 and *d* = 2, 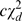 is asymptotically equivalent to an *F* distribution with degrees of freedom 2 and *n*−4. *F*(2, *n*−4) is the distribution of *F_j_*_|*i*_ when *X* is fixed [6],[12]. Our results demonstrate that for the random design matrix, the weighted chi-square distribution (15) is more appropriate.

#### Power of exhaustive search procedure

With the distribution of test statistics derived above, we first calculate the probability of exhaustive search to identify the exact true model. Under power definition (A), the test statistic *F*_12_ for the exact true model corresponds to the “alternative” distribution, whereas the *F*-statistics for all other models such as totally incorrect models and “half” correct models are combined together to generate a mixed “null” distribution. Let *S*_1_≡{*F_ij_*, *i* = 1, 2, *j* = 3,…,*p*}, *S*_2_≡{*F_jk_*, 3≤*j*<*k*≤*p*}, and *F_S_*_,[*R*]_ denote the *R*th largest variable in a set *S*. When controlling the false discovery number by *R*, the probability of detecting the exact true model (2) is
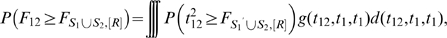
where *g*(*t*_12_,*t*_1_,*t*_2_) is the PDF of (11), 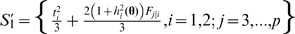 from the decomposition (14), and
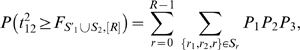
in which
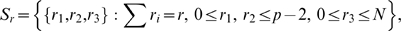


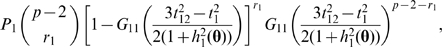


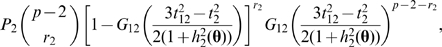


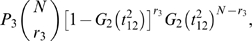


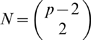
 is the number of variables in *S*_2_, *G*_1*i*_(•) is the CDF of distribution (15) for *i* = 1, 2, and *G*_2_(•) is the CDF of distribution (13). The test statistics within the sets *S* ^*^≡{*F_j_*_|1_, *F_j_*_|2_, *j* = 3,…,*p*} and *S*_2_≡{*F_jk_*, 3≤*j*<*k*≤*p*} are treated as asymptotically independent as *p*→∞ (see Text S1 Section 1.3 for details).

According to the power definition (B), the probability of exhaustive search to detect at least one of the associated SNPs is
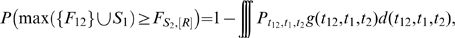
where
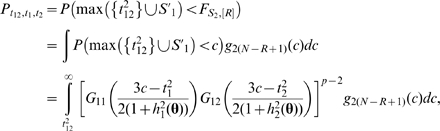

*g*_2(*N*−*R*+1)_(•) is the PDF of the (*N*−*R*+1)th order statistics distribution with the following density function:
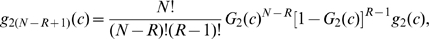

*G*_2_(•) and *g*_2_(•) are the CDF and PDF of the distribution of (13) respectively.

If *R* is neither too small nor too large, i.e. *R/N*→*c*, 0<*c*<1, as *N*→∞, we can use quantiles to replace the order statistics in order to simplify the calculation [20], i.e., 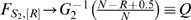. So for a given (*t*_12_,*t*_1_,*t*_2_), we can approximately replace the integrand 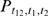 with
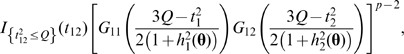
where *I_A_*(*x*) denotes the indicator function of set *A*. Simulations (results not shown) illustrate that the approximation of integrand is reasonably accurate for the integration.

### Forward Search

#### Statistics and asymptotic distributions

For forward search, first we derive the distributions of test statistics, which will be used to calculate the corresponding statistical power. Here we need to handle the comparison between two models: the model with SNPs 1 and *j*, *j* = 3, …, *p*, taking form (4), and the model with SNP *j* taking form (3). Let *F*_1|*j*_ be the *F* statistic measuring the significance of the extra terms in the bigger model over the smaller model [6]. Define 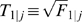. When *b*_1_+*b*_3_(*p*_2_−*q*_2_)≠0, following the asymptotic result in (5), we can derive
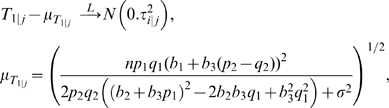
(16)and the formula for 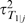 with *j* = 3 as an example is provided in Text S1 Section 4.3. Both 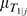 and 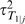 do not depend on the allele frequency *p_j_*. When *b*_1_+*b*_3_(*p*_2_−*q*_2_) = 0, 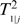 has a 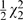 distribution by (7). Similarly we can get the asymptotic distribution of 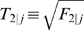 when comparing the model having SNPs 2 and *j* in form (4) with the model having SNP *j* in form (3). The covariance *Cov*(*T*_1|*j*_,*T*_2|*j*_) can also be calculated. As an example the formula of *Cov*(*T*_1|3_,*T*_2|3_) is given in Text S1 Section 4.3.

Moreover, the statistics (*T_1_*,*T_2_*,*T*_1|*j*_,*T*_2|*j*_)′ involving true SNPs have a multivariate normal distribution:
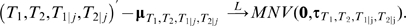
(17)When *j* = 3, the details of the calculation and the formulas for 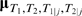 and 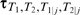 are given in Text S1 Sections 2.6 and 4.6.

Through result (6), we have proved that *T_j_* and *F*_1|*j*_ are asymptotically independent (refer to Text S1 Section 2.6 for details), i.e.
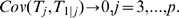
(18)When comparing the model having two incorrect SNPs *j* and *k* (3≤*j*<*k*≤*p*) in form (4) with the model having SNP *j* in form (3), the corresponding *F*-statistic *F_k_*_|*j*_ has the asymptotic distribution
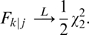
(19)Based on the result in (7), Text S1 Section 2.7 shows the proof for (19) with *j* = 3 and *k* = 4 as an example. This distribution is consistent with *F*(2, *n*−4) which can be derived with the fixed design matrix and is routinely used for *F_k_*_|*j*_ in the traditional model comparison [6],[12].

#### Power of forward search procedure

In the forward search procedure, we first apply marginal search to find the most significant SNP among models (3). Based on the selected SNP, we then fit models (4) in the second step to find the SNPs that have strong joint effects, while controlling for *R* false discoveries. Under power definition (A) for finding the exact true model, we need to calculate the probability of forward search to choose SNP 1 or 2 in the first step, and then pick up the true model in the second step. Define i^*^≡argmax*_i_*_ = 1,2_{|*T_i_*|}, 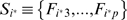, as *p*→∞, we can write the power as
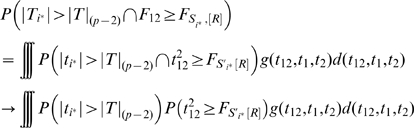
where *g*(*t*_12_,*t*_1_,*t*_2_) is the PDF of (*T*_12_,*T*_1_,*T*_2_)′ given in (11), 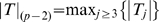, 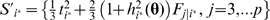 by *F*-statistic decomposition (14), and
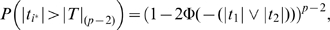


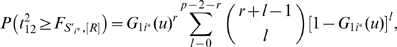
where 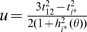, 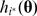 is given in (8) for i^*^ = 1, *r* = *p*−2−*R*+1, and 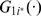 is the CDF of the distribution for 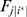 given in (15). i^*^ is fixed for an observed value (*t*_1_,*t*_2_)′ of random vector (*T*_1_,*T*_2_)′, so it is easy to implement the power calculation with Monte Carlo integration.

Note that 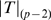 and 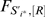 are asymptotically independent. This is because 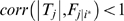 for each *j*≥3, so with *p*→∞, 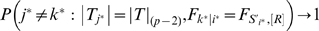. But when j^*^≠k^*^, 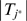 and 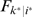 are always independent.

When *R* and *p* are large, we can simplify the formula of 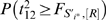 by approximating the *R*th largest variable in set 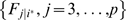 with 
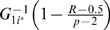
, where 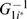 is the quantile function of 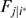. So we can approximately replace 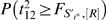 with 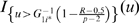 for calculating the integration.

Under power definition (B), the power of forward model selection method is the sum of *P_A_*: the probability to detect SNP 1 or 2 in the 1st step, and *P_B_*: the probability that step 1 fails but step 2 picks up at least one correct SNP, while controlling for *R* incorrect models as false positives. Specifically,
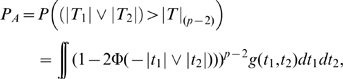
where *g*(*t*_1_,*t*_2_) is the PDF of joint distribution of (*T*_1_,*T*_2_)′ given in (9). Defining 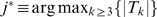 and 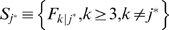, we have
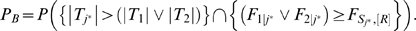



For each *k*≥3, *F_i_*_|*k*_ and *T_k_* are independent, so 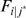 and 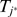 are independent. Given the results in (16) and (19), the distribution of 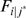 does not depend on j^*^. Hence, 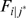 has the same distribution as *F_i_*_|*j*_, *j* = 3,…,*p*. We then have
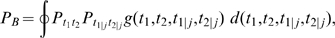
where *g*(*t_1_*,*t_2_*,*t*_1|*j*_,*t*_2|*j*_) is the PDF of (*T_1_*,*T_2_*,*T*_1|*j*_,*T*_2|*j*_)′ given in (17),
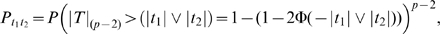


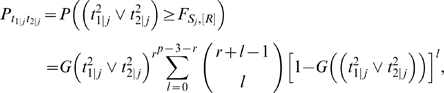
in which *r* = *p*−3−*R*+1, and *G*(•) is the CDF of *F_k_*_|*j*_, 3≤*j*<*k*≤*p*, given in (19). We can approximate 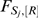 through the quantile function 
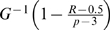
 to simplify the calculation of integration.

### Calculating Post-Hoc Power with a Given Marginal Model

To demonstrate how to evaluate the power of model selection methods in the empirical analysis, we have applied our approach in a real study example. In this example, the simple regression model on *X*_1_, 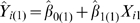, is an estimate of marginal model
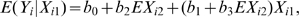
based on the full model (2). So the estimator of main effect is 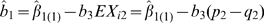. Similarly 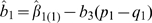, where 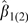 is given in the simple regression model on *X*_2_. To estimate the variance of random error, note that
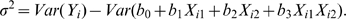
Therefore,
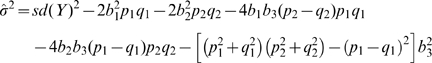
With an assumed value of *b*_3_ and the corresponding estimators 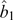, 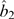, and 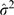, we can apply the above calculation to obtain the power of model selection strategies.

## Supporting Information

Text S1 Supplementary Note for proofs and arguments, distributions of test statistics, extended comparisons of power for model selection methods, and formulas for distribution parameters of test statistics.
(0.91 MB PDF)
