# Supplementary material for: Statistical Power of Model Selection Strategies for Genome-Wide Association Studies
Source: PLoS Genet. 2009 Jul 31;5(7):e1000582. doi: 10.1371/journal.pgen.1000582 (PMC2712761; doi:10.1371/journal.pgen.1000582)
Supplement: Text S1 — Supplementary Note for proofs and arguments, distributions of test statistics, extended comparisons of power for model selection methods, and formulas for distribution parameters of test statistics. (0.91 MB PDF) [file pgen.1000582.s001.pdf]

STATISTICAL POWER OF MODEL SELECTION STRATEGIES FOR

GENOME-WIDE ASSOCIATION STUDIES

Text S1

By Zheyang Wu, Hongyu Zhao

1 Proofs and arguments

1.1 Proof of the asymptotic distribution results

When $\nabla h(\boldsymbol{\theta}) \neq \mathbf{0}$, by Taylor expansion $h(\bar{\mathbf{Z}}) = h(\boldsymbol{\theta}) + (\bar{\mathbf{Z}} - \boldsymbol{\theta})' (\nabla h(\boldsymbol{\theta}) + R_n(\bar{\mathbf{Z}}, \boldsymbol{\theta}))$, so

$$\sqrt{n}\left(h\left(\bar{\mathbf{Z}}\right) - h(\boldsymbol{\theta})\right) = \sqrt{n}\left(\bar{\mathbf{Z}} - \boldsymbol{\theta}\right)' \left(\nabla h(\boldsymbol{\theta}) + R_n\left(\bar{\mathbf{Z}}, \boldsymbol{\theta}\right)\right).$$

By Central Limit Theory, $\sqrt{n}\left(\bar{\mathbf{Z}} - \boldsymbol{\theta}\right) \xrightarrow{L} N(0, \Sigma)$, so $\bar{\mathbf{Z}} \xrightarrow{P} \boldsymbol{\theta}$, and thus $R_n\left(\bar{\mathbf{Z}}, \boldsymbol{\theta}\right) \xrightarrow{P} 0$. Following this, we get

$$\sqrt{n}\left(h_1\left(\bar{\mathbf{Z}}\right) - h_1(\boldsymbol{\theta})\right) \xrightarrow{L} (\nabla h_1(\boldsymbol{\theta}))' N(0, \Sigma),$$
$$\sqrt{n}\left(h_2\left(\bar{\mathbf{Z}}\right) - h_2(\boldsymbol{\theta})\right) \xrightarrow{L} (\nabla h_2(\boldsymbol{\theta}))' N(0, \Sigma),$$

so that

$$Cov\left(\sqrt{n}h_1\left(\bar{\mathbf{Z}}\right), \sqrt{n}h_2\left(\bar{\mathbf{Z}}\right)\right) \xrightarrow{P} (\nabla h_1(\theta))' \Sigma (\nabla h_2(\boldsymbol{\theta})).$$

When $\nabla h(\boldsymbol{\theta}) = 0$, $h\left(\bar{\mathbf{Z}}\right) = h(\boldsymbol{\theta}) + \frac{1}{2}\left(\bar{\mathbf{Z}} - \boldsymbol{\theta}\right)' D^2 h(\boldsymbol{\theta} + \boldsymbol{\delta})\left(\bar{\mathbf{Z}} - \boldsymbol{\theta}\right)$ so that

$$n\left[h\left(\bar{\mathbf{Z}}\right) - h(\boldsymbol{\theta})\right] = \frac{1}{2}\sqrt{n}\left(\bar{\mathbf{Z}} - \boldsymbol{\theta}\right)' D^2 h(\boldsymbol{\theta} + \boldsymbol{\delta})\sqrt{n}\left(\bar{\mathbf{Z}} - \boldsymbol{\theta}\right).$$

By Central Limit Theory again,

$$n\left[h\left(\bar{\mathbf{Z}}\right) - h(\boldsymbol{\theta})\right] \xrightarrow{L} \frac{1}{2}\left[N(0, \Sigma)\right]' D^2 h(\boldsymbol{\theta})\left[N(0, \Sigma)\right].$$

Define $A \equiv D^2 h(\boldsymbol{\theta}) \Sigma$, if A is idempotent, we have (by [Searle(1971)], page 57), $n\left[h\left(\bar{\mathbf{Z}}\right) - h(\boldsymbol{\theta})\right] \xrightarrow{L} \frac{1}{2}\chi^2_{rank[A]}$. If A is not idempotent, by a theorem ([Searle(1971)], page 55),

$$E\left(n\left[h\left(\bar{\mathbf{Z}}\right) - h(\boldsymbol{\theta})\right]\right) \to \frac{1}{2}tr(A),$$

and

$$Var\left(n\left[h\left(\bar{\mathbf{Z}}\right) - h(\boldsymbol{\theta})\right]\right) \to \frac{1}{2}tr\left(A^2\right).$$

We can use $c\chi^2_d$ to approximate it ([Scheffé(1959)], page 414) by solving equations $cd = \frac{1}{2}tr(A)$ and $2c^2 d = \frac{1}{2}tr\left(A^2\right)$ for c and d.

1.2 Decomposition of F-statistics F_{13}

First note that the sum of squares can be decomposed as

$$\begin{aligned} SST &= SSM_1 + SSM_{3|1} + SSE_{13}, \\ SSM_{13} &= SSM_1 + SSM_{3|1}, \\ SSE_1 &= SSM_{3|1} + SSE_{13}, \end{aligned}$$

where SST is the total sum of squares, SSM_1 and SSE_1 are respectively the model sum of squares and error sum of squares for the single SNP model in the article equation (3) involving SNP 1. SSM_{13} and SSE_{13} are respectively

the model sum of squares and error sum of squares for the full epistatic model in the article equation (4) involving SNPs 1 and 3. $SSM_{3|1}$ is the extra variation (sum of squares) explained by the full model compared with the single SNP model. The F-statistics comparing the two models is

$$F_{3|1} = \frac{(SSM_{13} - SSM_1)/2}{SSE_{13}/(n-4)}.$$

So we have

$$\begin{aligned} F_{13} &= \frac{SSM_{13}/3}{SSE_{13}/(n-4)} \\ &= \frac{n-4}{3}\left[\frac{SSM_1}{SSE_1}\frac{SSM_{3|1}+SSE_{13}}{SSE_{13}} + \frac{SSM_{3|1}}{SSE_{13}}\right] \\ &= \frac{n-4}{3}\left[\frac{F_1}{n-2}\left(1+\frac{2F_{3|1}}{n-4}\right)+\frac{2F_{3|1}}{n-4}\right] \\ &\to \frac{1}{3}F_1 + \frac{2}{3}h_1^2(\boldsymbol{\theta})F_{3|1} + \frac{2}{3}F_{3|1}, \end{aligned}$$

since $\frac{1}{n}F_1 = \frac{1}{n}T_1^2 \xrightarrow{P} h_1^2(\boldsymbol{\theta})$, where $h_1(\boldsymbol{\theta})$ is defined in the article equation (8).

1.3 Independency dominates sets S^* and S_2

In set

$$S^* \equiv \left\{F_{j|i}, i=1,2,\ j=3,...,p\right\},$$

$F_{j|1}$ and $F_{j|2}$ are correlated but $F_{j|1}$ and $F_{k|1}$ (or $F_{k|2}$), $j \neq k$, are independent. So the average pair-wise correlation within set is decreasing to 0 at the rate of

$$\frac{\#\text{ of correlated pairs within } S_1}{\#\text{ of total pairs within } S_1} = \frac{(p-2)}{\binom{2(p-2)}{2}} = \frac{1}{2p-5}.$$

So as $p \to \infty$, the order statistics and quantiles of S^* converge to those when the elements in S^* are independent ([Rawlings(1976)], [David(1981)] Chapter 10.7). Since p is large in GWAS, we can approximately treat the statistics in S^* to be independent in the context of power calculation in the article Methods section, where only the order statistics and quantiles are relevant.

There exists a complex correlation structure within $S_2 \equiv \{F_{jk}, j<k=3,...,p\}$ since all test statistics from the models sharing one common SNP are correlated. However, the average pair-wise correlation within this set is decreasing to 0 at the rate of $\frac{4}{p-1}$. Similarly, we approximately treat the statistics in S_2 as being independent when they are considered for null distribution. Furthermore, we assume S_1 and S_2 are independent since the average pair-wise correlation between these two sets is again decreasing to 0 at the rate of $\frac{1}{p-3}$.

2 Distributions for test statistics

The asymptotic distributions for the relevant test statistics in three model selection methods are derived as following. These formulas are used in the Methods section of the article.

2.1 Asymptotic distribution of T_1

T_1, the T-statistic in the simple regression model involving SNP 1 is given by formula

$$T_1 = \frac{\frac{(X_1 - \bar{X}_1\mathbf{1})'Y}{\|X_1-\bar{X}_1\mathbf{1}\|}}{\sqrt{\frac{Y'(I-P_1)Y}{n-2}}},$$

where $X_1 = (X_{11},...,X_{n1})'$ is the vector of observed genotypes at SNP 1, with sample mean $\bar{X}_1 = \frac{1}{n}\sum_{i=1}^n X_{i1}$. I is an $n \times n$ identity matrix, $P_1 = \mathbf{X}_1(\mathbf{X}_1'\mathbf{X}_1)\mathbf{X}_1'$ is the projection matrix onto the vector space spanned by $\mathbf{X}_1 = (\mathbf{1}, X_1)$. $Y = (Y_1,...,Y_n)'$ is the observed trait vector generated by true model in the article equation (2).

Note the constant intercept b_0 does not affect the value of T- or F-statistics of the model. So in the following, we assume $b_0 = 0$.

To rewrite T_1 as a function of sample means, note that in the numerator of T_1,

$$\left\|X_1 - \bar{X}_1 \mathbf{1}\right\|^2 = \sum \left(X_{i1} - \bar{X}_1\right)^2 = \sum X_{i1}^2 - n\overline{X_1}^2 = n\left(\overline{X_1^2} - \overline{X_1}^2\right).$$

and

$$\begin{aligned} \left(X_1 - \bar{X}_1 \mathbf{1}\right)' Y &= \sum \left(X_{i1} - \bar{X}_1\right)\left(b_1 X_{i1} + b_2 X_{i2} + b_3 X_{i1} X_{i2} + \varepsilon_i\right) \\ = \; & n\left(b_1\left(\overline{X_1^2} - \overline{X_1}^2\right) + b_2\left(\overline{X_1 X_2} - \bar{X}_1 \bar{X}_2\right) + b_3\left(\overline{X_1^2 X_2} - \bar{X}_1 \overline{X_1 X_2}\right) + \overline{X_1 \varepsilon} - \overline{X_1 \bar{\varepsilon}}\right), \end{aligned}$$

For the denominator of T_1 we have

$$Y'\left(I - P_1\right) Y = Y'\left(I - \frac{J}{n}\right) Y - Y' P_{\left(X_1 - \bar{X}_1 \mathbf{1}\right)} Y,$$

where $J = \mathbf{1}\mathbf{1}'$ is the $n \times n$ matrix of elements 1, $P_{\left(X_1 - \bar{X}_1 \mathbf{1}\right)}$ is the projection matrix onto space spanned by vector $X_1 - \bar{X}_1 \mathbf{1}$, i.e.

$$P_{\left(X_1 - \bar{X}_1 \mathbf{1}\right)} = \frac{\left(X_1 - \bar{X}_1 \mathbf{1}\right)\left(X_1 - \bar{X}_1 \mathbf{1}\right)'}{\sum \left(X_{i1} - \bar{X}_1\right)^2},$$

and

$$\begin{aligned} Y'\left(I - \frac{J}{n}\right) Y &= \left(Y - \bar{Y}\mathbf{1}\right)'\left(Y - \bar{Y}\mathbf{1}\right) \\ = \; & n\left\{\begin{array}{c} b_1^2\left(\overline{X_1^2} - \bar{X}_1^2\right) + 2b_1 b_2\left(\overline{X_1 X_2} - \bar{X}_1 \bar{X}_2\right) + 2b_1 b_3\left(\overline{X_1^2 X_2} - \bar{X}_1 \overline{X_1 X_2}\right) \\ +2b_1\left(\overline{X_1 \varepsilon} - \bar{X}_1 \bar{\varepsilon}\right) + b_2^2\left(\overline{X_2^2} - \bar{X}_2^2\right) + 2b_2 b_3\left(\overline{X_1 X_2^2} - \bar{X}_2 \overline{X_1 X_2}\right) \\ +2b_2\left(\overline{X_2 \varepsilon} - \bar{X}_2 \bar{\varepsilon}\right) + b_3^2\left(\overline{X_1^2 X_2^2} - \overline{X_1 X_2}^2\right) + 2b_3\left(\overline{X_1 X_2 \varepsilon} - \overline{X_1 X_2} \bar{\varepsilon}\right) + \overline{\varepsilon^2} - \bar{\varepsilon}^2 \end{array}\right\}. \end{aligned}$$

So we can rewrite T_1 as a function of sample means

$$T_1 = \sqrt{n-2}\, h_1\left(\bar{\mathbf{Z}}\right),$$

where

$$\mathbf{Z}_i = \left(\varepsilon, X_{i1}, X_{i1}^2, X_{i2}, X_{i2}^2, X_{i1} X_{i2}, X_{i1}^2 X_{i2}, X_{i1}\varepsilon_i, X_{i1} X_{i2}^2, X_{i1}^2 X_{i2}^2, \varepsilon_i^2, X_{i2}\varepsilon_i, X_{i1} X_{i2} \varepsilon_i\right).$$

Let the population mean $\boldsymbol{\theta} = E\left(\mathbf{Z}_i\right)$, under the setup of genotype scale and allele frequency in the article equation (1), the elements of θ are:

$E\varepsilon_i = 0 = EX_1\varepsilon_i = EX_2\varepsilon_i = EX_1X_2\varepsilon_i$,
$EX_{i1} = p_1 - q_1$,
$EX_{i1}^2 = p_1^2 + q_1^2$,
$EX_{i2} = p_2 - q_2$,
$EX_{i2}^2 = p_2^2 + q_2^2$,
$EX_{i1}X_{i2} = (p_1 - q_1)(p_2 - q_2)$,
$EX_{i1}^2 X_{i2} = (p_1^2 + q_1^2)(p_2 - q_2)$,
$EX_{i1} X_{i2}^2 = (p_1 - q_1)(p_2^2 + q_2^2)$,
$EX_{i1}^2 X_{i2}^2 = (p_1^2 + q_1^2)(p_2^2 + q_2^2)$,
$E\varepsilon_i^2 = \sigma^2$.

The variance-covariance matrix of the random vector $\mathbf{Z}_i$ is $\Sigma = Var\left(\mathbf{Z}_i\right)$, with the nonzero variance and covariance elements being:

$Var\left(\varepsilon_i\right) = \sigma^2$,
$Var\left(X_{i1}\right) = 2p_1q_1$,
$Var\left(X_{i1}^2\right) = (p_1^2 + q_1^2)2p_1q_1$,
$Var\left(X_{i2}\right) = 2p_2q_2$,
$Var\left(X_{i2}^2\right) = (p_2^2 + q_2^2)2p_2q_2$,

$$
\begin{aligned}
&Var\,(X_{i1}X_{i2}) = (p_1^2+q_1^2)(p_2^2+q_2^2) - (p_1-q_1)^2(p_2-q_2)^2,\\
&Var\left(X_{i1}^2X_{i2}\right) = (p_1^2+q_1^2)(p_2^2+q_2^2) - (p_1^2+q_1^2)^2(p_2-q_2)^2,\\
&Var\,(X_{i1}\varepsilon) = (p_1^2+q_1^2)\sigma^2,\\
&Var\left(X_{i1}X_{i2}^2\right) = (p_1^2+q_1^2)(p_2^2+q_2^2) - (p_1-q_1)^2(p_2^2+q_2^2)^2,\\
&Var\left(X_{i1}^2X_{i2}^2\right) = (p_1^2+q_1^2)(p_2^2+q_2^2) - (p_1^2+q_1^2)^2(p_2^2+q_2^2)^2,\\
&Var\left(\varepsilon_i^2\right) = 2\sigma^4,\\
&Var\,(X_{i2}\varepsilon_i) = (p_2^2+q_2^2)\sigma^2,\\
&Var\,(X_{i1}X_{i2}\varepsilon_i) = (p_1^2+q_1^2)(p_2^2+q_2^2)\sigma^2,\\
&Cov\,(\varepsilon_i, X_{i1}\varepsilon_i) = (p_1-q_1)\sigma^2,\\
&Cov\,(\varepsilon_i, X_{i2}\varepsilon_i) = (p_2-q_2)\sigma^2,\\
&Cov\,(\varepsilon_i, X_{i1}X_{i2}\varepsilon_i) = (p_1-q_1)(p_2-q_2)\sigma^2,\\
&Cov\left(X_{i1}, X_{i1}^2\right) = (p_1-q_1)2p_1q_1,\\
&Cov\,(X_{i1}, X_{i1}X_{i2}) = (p_2-q_2)2p_1q_1,\\
&Cov\left(X_{i1}, X_{i1}^2X_{i2}\right) = (p_1-q_1)(p_2-q_2)2p_1q_1,\\
&Cov\left(X_{i1}, X_{i1}X_{i2}^2\right) = (p_2^2+q_2^2)2p_1q_1,\\
&Cov\left(X_{i1}, X_{i1}^2X_{i2}^2\right) = (p_1-q_1)(p_2^2+q_2^2)2p_1q_1,\\
&Cov\left(X_{i1}^2, X_{i1}X_{i2}\right) = (p_1-q_1)(p_2-q_2)2p_1q_1,\\
&Cov\left(X_{i1}^2, X_{i1}^2X_{i2}\right) = (p_1^2+q_1^2)(p_2-q_2)2p_1q_1,\\
&Cov\left(X_{i1}^2, X_{i1}X_{i2}^2\right) = (p_1-q_1)(p_2^2+q_2^2)2p_1q_1,\\
&Cov\left(X_{i1}^2, X_{i1}^2X_{i2}^2\right) = (p_1^2+q_1^2)(p_2^2+q_2^2)2p_1q_1,\\
&Cov\left(X_{i2}, X_{i2}^2\right) = (p_2-q_2)2p_2q_2,\\
&Cov\,(X_{i2}, X_{i1}X_{i2}) = (p_1-q_1)2p_2q_2,\\
&Cov\left(X_{i2}, X_{i1}^2X_{i2}\right) = (p_1^2+q_1^2)2p_2q_2,\\
&Cov\left(X_{i2}, X_{i1}X_{i2}^2\right) = (p_1-q_1)(p_2-q_2)2p_2q_2,\\
&Cov\left(X_{i2}, X_{i1}^2X_{i2}^2\right) = (p_1^2+q_1^2)(p_2-q_2)2p_2q_2,\\
&Cov\left(X_{i2}^2, X_{i1}X_{i2}\right) = (p_1-q_1)(p_2-q_2)2p_2q_2,\\
&Cov\left(X_{i2}^2, X_{i1}^2X_{i2}\right) = (p_2-q_2)(p_1^2+q_1^2)2p_2q_2,\\
&Cov\left(X_{i2}^2, X_{i1}X_{i2}^2\right) = (p_1-q_1)(p_2^2+q_2^2)2p_2q_2,\\
&Cov\left(X_{i2}^2, X_{i1}^2X_{i2}^2\right) = (p_1^2+q_1^2)(p_2^2+q_2^2)2p_2q_2,\\
&Cov\left(X_{i1}X_{i2}, X_{i1}^2X_{i2}\right) = (p_1-q_1)(p_2^2+q_2^2-(p_2-q_2)^2(p_1^2+q_1^2)),\\
&Cov\left(X_{i1}X_{i2}, X_{i1}X_{i2}^2\right) = (p_2-q_2)(p_1^2+q_1^2-(p_1-q_1)^2(p_2^2+q_2^2)),\\
&Cov\left(X_{i1}X_{i2}, X_{i1}^2X_{i2}^2\right) = (p_1-q_1)(p_2-q_2)(1-(p_1^2+q_1^2)(p_2^2+q_2^2)),\\
&Cov\left(X_{i1}^2X_{i2}, X_{i1}X_{i2}^2\right) = (p_1-q_1)(p_2-q_2)(1-(p_1^2+q_1^2)(p_2^2+q_2^2)),\\
&Cov\left(X_{i1}^2X_{i2}, X_{i1}^2X_{i2}^2\right) = (p_1^2+q_1^2)(p_2-q_2)(1-(p_1^2+q_1^2)(p_2^2+q_2^2)),\\
&Cov\,(X_{i1}\varepsilon_i, X_{i2}\varepsilon_i) = (p_1-q_1)(p_2-q_2)\sigma^2,\\
&Cov\,(X_{i1}\varepsilon_i, X_{i1}X_{i2}\varepsilon_i) = (p_1^2+q_1^2)(p_2-q_2)\sigma^2,\\
&Cov\left(X_{i1}X_{i2}^2, X_{i1}^2X_{i2}^2\right) = (p_1-q_1)(p_2^2+q_2^2)(1-(p_1^2+q_1^2)(p_2^2+q_2^2)),\\
&Cov\,(X_{i2}\varepsilon_i, X_{i1}X_{i2}\varepsilon_i) = (p_1-q_1)(p_2^2+q_2^2)\sigma^2.
\end{aligned}
$$

By the asymptotic distribution result in the article equation (5), we can derive (with Mathematica 5 [Wolfram(1999)]) that

$$T_1 - \sqrt{n-2}h_1(\boldsymbol{\theta}) = \sqrt{n-2}\left(h_1\left(\bar{\mathbf{Z}}\right) - h_1(\boldsymbol{\theta})\right) \xrightarrow{L} N\left(0, \tau_1^2\right),$$

where $h_1(\boldsymbol{\theta})$ is given in the article equation (8), τ_1^2 is given in Supplementary Note Section 4.1.

2.2 Asymptotic distribution for T_3

T_3 is the T statistic for simple regression model $\hat{Y}_{i(3)} = \hat{\beta}_{0(3)} + \hat{\beta}_{1(3)}X_{i3}$:

$$T_3 = \frac{\frac{\sum\left(X_{i3}-\bar{X}_3\right)Y_i}{\sqrt{\sum\left(X_{i3}-\bar{X}_3\right)^2}}}{\sqrt{\frac{Y'(I-P_3)Y}{n-2}}},$$

where $\bar{X}_3 = \frac{1}{n}\sum_{i=1}^{n} X_{i3}$, $P_3 = \mathbf{X}_3\left(\mathbf{X}_3'\mathbf{X}_3\right)^{-1}\mathbf{X}_3'$ is the projection matrix onto the vector space spanned by $\mathbf{X}_3 = (\mathbf{1}, X_3)'$.

To rewrite T_3 as a function of sample means, note that $\sum\left(X_{i3}-\bar{X}_3\right)^2 = \sum X_{i3}^2 - n\bar{X}_3^2 = n\left(\overline{X_3^2} - \bar{X}_3^2\right)$. Similarly,

$$\begin{aligned}&\left(X_3-\bar{X}_3\mathbf{1}\right)'Y = \left(X_3-\bar{X}_3\mathbf{1}\right)'\left(b_1X_1+b_2X_2+b_3X_1X_2+\varepsilon\right)\\ =\;& n\left(b_1\left(\overline{X_1X_3}-\bar{X}_1\bar{X}_3\right)+b_2\left(\overline{X_2X_3}-\bar{X}_2\bar{X}_3\right)+b_3\left(\overline{X_1X_2X_3}-\bar{X}_3\overline{X_1X_2}\right)+\overline{X_3\varepsilon}-\bar{X}_3\bar{\varepsilon}\right).\end{aligned}$$

The projection matrix $P_3 = P_{\left(X_3-\bar{X}_3\mathbf{1}\right)} + \frac{J}{n}$, where $P_{\left(X_3-\bar{X}_3\mathbf{1}\right)}$ is the projection matrix onto space spanned by vector $X_3 - \bar{X}_3\mathbf{1}$, i.e.

$$\begin{aligned}P_{\left(X_3-\bar{X}_3\mathbf{1}\right)} &= \left(X_3-\bar{X}_3\mathbf{1}\right)\left(\left(X_3-\bar{X}_3\mathbf{1}\right)'\left(X_3-\bar{X}_3\mathbf{1}\right)\right)^{-1}\left(X_3-\bar{X}_3\mathbf{1}\right)'\\ &= \frac{\left(X_3-\bar{X}_3\mathbf{1}\right)\left(X_3-\bar{X}_3\mathbf{1}\right)'}{\sum\left(X_{i3}-\bar{X}_3\right)^2}.\end{aligned}$$

For the denominator of T_3 we have

$$Y'\left(I-P_3\right)Y = Y'\left(I-\frac{J}{n}\right)Y - Y'P_{\left(X_3-\bar{X}_3\mathbf{1}\right)}Y.$$

So we can rewrite T_3 as a function of sample means

$$T_3 = \sqrt{n-2}\,h_3\left(\bar{\mathbf{Z}}\right),$$

where

$$\mathbf{Z}_i = \left(\begin{array}{c}\varepsilon_i, X_{i1}, X_{i1}^2, X_{i2}, X_{i2}^2, X_{i1}X_{i2}, X_{i1}^2X_{i2}, X_{i1}\varepsilon_i, X_{i1}X_{i2}^2, X_{i1}^2X_{i2}^2, \varepsilon_i^2, X_{i2}\varepsilon_i, X_{i1}X_{i2}\varepsilon_i, X_{i3},\\ X_{i3}^2, X_{i3}\varepsilon_i, X_{i1}X_{i3}, X_{i2}X_{i3}, X_{i1}X_{i2}X_{i3}, X_{i1}^2X_{i3}, X_{i1}X_{i3}^2, X_{i1}^2X_{i3}^2, X_{i1}^2X_{i2}X_{i3}, X_{i1}X_{i3}\varepsilon_i\end{array}\right).$$

Based on the same argument for T_1 above in the Supplementary Note Section 2.1, we have

$$T_j \xrightarrow{L} N(0,1).$$

2.3 Asymptotic distribution for F_{12}

Let F_{12} be the F-statistics for the full regression model with interaction involving true SNPs 1 and 2.

$$F_{12} = \frac{\frac{Y'\left(P_{12}-\frac{J}{n}\right)Y}{3}}{\frac{Y'\left(I-P_{12}\right)Y}{n-4}},$$

where $P_{12} = \mathbf{X}_{12}\left(\mathbf{X}_{12}'\mathbf{X}_{12}\right)^{-1}\mathbf{X}_{12}'$ is the projection matrix onto the vector space spanned by $\mathbf{X}_{12} = (\mathbf{1}, X_1, X_2, X_1X_2)$, $X_1X_2 = (X_{11}X_{12}, ..., X_{n1}X_{n2})'$, $J = \mathbf{1}\mathbf{1}'$. Further, $P_{12} - \frac{J}{n} = \tilde{P}_{12} = \tilde{\mathbf{X}}_{12}\left(\tilde{\mathbf{X}}_{12}'\tilde{\mathbf{X}}_{12}\right)^{-1}\tilde{\mathbf{X}}_{12}'$ is the projection matrix onto the vector space spanned by $\tilde{\mathbf{X}}_{12} = \left(X_1-\bar{X}_1\mathbf{1},\ X_2-\bar{X}_2\mathbf{1},\ X_1X_2-\overline{X_1X_2}\mathbf{1}\right)$, so that

$$\begin{aligned}\tilde{\mathbf{X}}_{12}'Y &= \left(\left(X_1-\bar{X}_1\mathbf{1}\right)'Y,\ \left(X_2-\bar{X}_2\mathbf{1}\right)'Y,\ \left(X_1X_2-\overline{X_1X_2}\mathbf{1}\right)'Y\right)\\ &= n\left(\begin{array}{c} b_1\left(\overline{X_1^2}-\overline{X_1}^2\right)+b_2\left(\overline{X_1X_2}-\bar{X}_1\bar{X}_2\right)+b_3\left(\overline{X_1^2X_2}-\bar{X}_1\overline{X_1X_2}\right)+\overline{X_1\varepsilon}-\overline{X_1}\bar{\varepsilon}\\ b_1\left(\overline{X_1X_2}-\bar{X}_1\bar{X}_2\right)+b_2\left(\overline{X_2^2}-\bar{X}_2^2\right)+b_3\left(\overline{X_1X_2^2}-\bar{X}_2\overline{X_1X_2}\right)+\overline{X_2\varepsilon}-\overline{X_2}\bar{\varepsilon}\\ b_1\left(\overline{X_1^2X_2}-\bar{X}_1\overline{X_1X_2}\right)+b_2\left(\overline{X_1X_2^2}-\bar{X}_2\overline{X_1X_2}\right)+b_3\left(\overline{X_1^2X_2^2}-\overline{X_1X_2}^2\right)+\overline{X_1X_2\varepsilon}-\overline{X_1X_2}\bar{\varepsilon}\end{array}\right),\end{aligned}$$

and

$$\tilde{\mathbf{X}}_{12}'\tilde{\mathbf{X}}_{12} = \left(\begin{array}{ccc}\overline{X_1^2}-\overline{X_1}^2 & \overline{X_1X_2}-\bar{X}_1\bar{X}_2 & \overline{X_1^2X_2}-\bar{X}_1\overline{X_1X_2}\\ \overline{X_1X_2}-\bar{X}_1\bar{X}_2 & \overline{X_2^2}-\bar{X}_2^2 & \overline{X_1X_2^2}-\bar{X}_2\overline{X_1X_2}\\ \overline{X_1^2X_2}-\bar{X}_1\overline{X_1X_2} & \overline{X_1X_2^2}-\bar{X}_2\overline{X_1X_2} & \overline{X_1^2X_2^2}-\overline{X_1X_2}^2\end{array}\right).$$

For the denominator part, since $X_1 P_{12} = X_1$, $X_2 P_{12} = X_2$, $X_1 X_2 P_{12} = X_1 X_2$,

$$\begin{aligned} Y'(I - P_{12}) Y &= \varepsilon'(I - P_{12})\varepsilon \\ &= \varepsilon'\left(I - \frac{J}{n} - \tilde{P}_{12}\right)\varepsilon \\ &= \varepsilon'\left(I - \frac{J}{n}\right)\varepsilon - \varepsilon'\tilde{P}_{12}\varepsilon \\ &= \overline{\varepsilon^2} - \bar{\varepsilon}^2 - \varepsilon'\tilde{\mathbf{X}}_{12}\left(\tilde{\mathbf{X}}_{12}'\tilde{\mathbf{X}}_{12}\right)^{-1}\tilde{\mathbf{X}}_{12}'\varepsilon, \end{aligned}$$

where

$$\tilde{\mathbf{X}}_{12}'\varepsilon = \begin{pmatrix} \overline{X_1\varepsilon} - \overline{X_1}\bar{\varepsilon} \\ \overline{X_2\varepsilon} - \overline{X_2}\bar{\varepsilon} \\ \overline{X_1X_2\varepsilon} - \overline{X_1X_2}\bar{\varepsilon} \end{pmatrix}.$$

So $T_{12} \equiv \sqrt{F_{12}}$ can be written as a function of sample means

$$T_{12} \equiv \sqrt{F_{12}} = \sqrt{n-4}\,h_{12}\left(\bar{\mathbf{Z}}\right),$$

where

$$\mathbf{Z}_i = \left(\varepsilon_i, X_{i1}, X_{i1}^2, X_{i2}, X_{i2}^2, X_{i1}X_{i2}, X_{i1}^2X_{i2}, X_{i1}\varepsilon_i, X_{i1}X_{i2}^2, X_{i1}^2X_{i2}^2, \varepsilon_i^2, X_{i2}\varepsilon_i, X_{i1}X_{i2}\varepsilon_i\right).$$

Based on the asymptotic distribution result in (5) in the article, we get

$$T_{12} - \mu_{T_{12}} \xrightarrow{L} N\left(0, \tau_{T12}^2\right),$$

where

$$\mu_{T_{12}} = \begin{pmatrix} \frac{2n}{3\sigma^2}\left(b_1^2 p_1 q_1 + b_3^2 p_1 q_1\left(p_2^2 + q_2^2\right) + 2b_1 b_3 p_1 q_1 (p_2 - q_2)\right. \\ \left. + p_2 q_2\left(b_2 + b_3\left(p_1 - q_1\right)\right)^2\right) \end{pmatrix}^{1/2},$$

and the formula of τ_{T12}^2 is given in the Supplementary Note Section 3.1.

Also by asymptotic result (6) in the article, we have

$$\begin{aligned} \tau_{12,1} &= Cov\left(T_{12}, T_1\right) \xrightarrow{P} \left[\nabla h_{12}(\boldsymbol{\theta})\right]'\Sigma\left[\nabla h_1(\boldsymbol{\theta})\right], \\ \tau_{12,2} &= Cov\left(T_{12}, T_2\right) \xrightarrow{P} \left[\nabla h_{12}(\boldsymbol{\theta})\right]'\Sigma\left[\nabla h_2(\boldsymbol{\theta})\right], \end{aligned}$$

where $h_1(\boldsymbol{\theta})$ is given in equation (8) of the article, the formula of $\tau_{12,1}$ is given in the Supplementary Note Section 3.1.

2.4 Asymptotic distribution for F_{34}

Let F_{34} be the F-statistics for the full interaction regression model fitting Y with X_3 and X_4,

$$F_{34} = \frac{\frac{Y'\left(P_{34} - \frac{J}{n}\right)Y}{3}}{\frac{Y'(I - P_{34})Y}{n-4}},$$

where $P_{34} = \mathbf{X}_{34}\left(\mathbf{X}_{34}'\mathbf{X}_{34}\right)\mathbf{X}_{34}'$ is the projection matrix onto the vector space spanned by $\mathbf{X}_{34} = \left(\mathbf{1}, X_3, X_4, X_3X_4\right)'$. $P_{34} - \frac{J}{n} = \tilde{P}_{34} = \tilde{\mathbf{X}}_{34}\left(\tilde{\mathbf{X}}_{34}'\tilde{\mathbf{X}}_{34}\right)^{-1}\tilde{\mathbf{X}}_{34}'$ is the projection matrix onto the vector space spanned by

$$\tilde{\mathbf{X}}_{34} = \left(X_3 - \bar{X}_3\mathbf{1},\ X_4 - \bar{X}_4\mathbf{1},\ X_3X_4 - \overline{X_3X_4}\mathbf{1}\right),$$

so that the numerator is $\frac{Y'\tilde{P}_{34}Y}{3}$, where

$$\begin{aligned} \tilde{\mathbf{X}}_{34}'Y &= \left(\left(X_3 - \bar{X}_3\mathbf{1}\right)'Y,\ \left(X_4 - \bar{X}_4\mathbf{1}\right)'Y,\ \left(X_3X_4 - \overline{X_3X_4}\mathbf{1}\right)'Y\right) \\ &= n\begin{pmatrix} b_1\left(\overline{X_1X_3} - \bar{X}_1\bar{X}_3\right) + b_2\left(\overline{X_2X_3} - \bar{X}_2\bar{X}_3\right) + b_3\left(\overline{X_1X_2X_3} - \bar{X}_3\overline{X_1X_2}\right) + \overline{X_3\varepsilon} - \overline{X_3}\bar{\varepsilon} \\ b_1\left(\overline{X_1X_4} - \bar{X}_1\bar{X}_4\right) + b_2\left(\overline{X_2X_4} - \bar{X}_2\bar{X}_4\right) + b_3\left(\overline{X_1X_2X_4} - \bar{X}_4\overline{X_1X_2}\right) + \overline{X_4\varepsilon} - \overline{X_4}\bar{\varepsilon} \\ b_1\left(\overline{X_1X_3X_4} - \bar{X}_1\overline{X_3X_4}\right) + b_2\left(\overline{X_2X_3X_4} - \bar{X}_2\overline{X_3X_4}\right) + b_3\left(\overline{X_1X_2X_3X_4} - \left(\overline{X_1X_2}\right)\left(\overline{X_3X_4}\right)\right) + \overline{X_3X_4\varepsilon} - \overline{X_3X_4}\bar{\varepsilon} \end{pmatrix}, \end{aligned}$$

and

$$\tilde{\mathbf{X}}_{34}'\tilde{\mathbf{X}}_{34} = \begin{pmatrix} \overline{X_3^2} - \overline{X_3}^2 & \overline{X_3X_4} - \bar{X}_3\bar{X}_4 & \overline{X_3^2X_4} - \bar{X}_3\overline{X_3X_4} \\ \overline{X_3X_4} - \bar{X}_3\bar{X}_4 & \overline{X_4^2} - \bar{X}_4^2 & \overline{X_3X_4^2} - \bar{X}_4\overline{X_3X_4} \\ \overline{X_3^2X_4} - \bar{X}_3\overline{X_3X_4} & \overline{X_3X_4^2} - \bar{X}_4\overline{X_3X_4} & \overline{X_3^2X_4^2} - \overline{X_3X_4}^2 \end{pmatrix}.$$

For the denominator part,

$$Y'\left(I - P_{34}\right)Y = Y'\left(I - \frac{J}{n}\right)Y - Y'\tilde{P}_{34}Y,$$

where $Y'\left(I - \frac{J}{n}\right)Y$ is given in the above Supplementary Note Section 2.1, $Y'\tilde{P}_{34}Y$ is given in the denominator.

Thus F_{34} can be rewritten as a function of sample means

$$F_{34} = (n-4)\,h_{34}\left(\bar{\mathbf{Z}}\right).$$

where

$$\mathbf{Z}_i = \begin{pmatrix} \varepsilon_i, X_{i1}, X_{i1}^2, X_{i2}, X_{i2}^2, X_{i1}X_{i2}, X_{i1}^2X_{i2}, X_{i1}\varepsilon_i, X_{i1}X_{i2}^2, X_{i1}^2X_{i2}^2, \varepsilon_i^2, X_{i2}\varepsilon_i, X_{i1}X_{i2}\varepsilon_i, \\ X_{i3}, X_{i3}^2, X_{i4}, X_{i4}^2, X_{i3}X_{i4}, X_{i3}^2X_{i4}, X_{i3}\varepsilon_i, X_{i3}X_{i4}^2, X_{i3}^2X_{i4}^2, X_{i4}\varepsilon_i, X_{i3}X_{i4}\varepsilon_i, X_{i1}X_{i3}, \\ X_{i2}X_{i3}, X_{i1}X_{i2}X_{i3}, X_{i1}X_{i4}, X_{i2}X_{i4}, X_{i1}X_{i2}X_{i4}, X_{i1}X_{i3}X_{i4}, X_{i2}X_{i3}X_{i4}, X_{i1}X_{i2}X_{i3}X_{i4} \end{pmatrix}.$$

Let $\boldsymbol{\theta} = E\left(\mathbf{Z}_i\right)$, $\Sigma = Var\left(\mathbf{Z}_i\right)$ (elements are not given here, but with similar calculation as in the above Supplementary Note Section 2.1). Calculation leads $h_{34}\left(\boldsymbol{\theta}\right) = 0$ and $\bigtriangledown h_{34}\left(\boldsymbol{\theta}\right) = 0$. The vector of eigenvalues of $D^2h\left(\boldsymbol{\theta}\right)\Sigma$ is $\left(\frac{2}{3}, \frac{2}{3}, \frac{2}{3}, 0, ...0\right)$, which indicates that $\frac{3}{2}D^2h\left(\boldsymbol{\theta}\right)\Sigma$ is idempotent. By the asymptotic distribution result in (7) of the article,

$$\begin{aligned} F_{34} &= (n-4)\,h_{34}\left(\bar{\mathbf{Z}}\right) \\ &\xrightarrow{L} \frac{1}{2}\frac{2}{3}\left[N\left(0, \sqrt{\frac{3}{2}}\Sigma\right)\right]' D^2h\left(\boldsymbol{\theta}\right)\left[N\left(0, \sqrt{\frac{3}{2}}\Sigma\right)\right] \\ &\overset{(d)}{=} \frac{1}{3}\chi_3^2, \end{aligned}$$

where the last equation is based on a theorem by Searle ([Searle(1971)], page 57).

2.5 Asymptotic distribution for $F_{3|1}$

$F_{3|1}$ is the F-statistic for comparing the full epistatic model

$$\hat{Y}_{i(13)} = \hat{\beta}_{0(13)} + \hat{\beta}_{1(13)}X_{i1} + \hat{\beta}_{2(13)}X_{i3} + \hat{\beta}_{3(13)}X_{i1}X_{i3}, \tag{1}$$

to the single SNP model

$$\hat{Y}_{i(1)} = \hat{\beta}_{0(1)} + \hat{\beta}_{1(1)}X_{i1}.$$

$F_{3|1}$ measures how much variation the full model can explain more than the simple model.

$$F_{3|1} = \frac{\left(SSM_{13} - SSM_1\right)/2}{SSE_{13}/\left(n-4\right)},$$

where $SSM_1 = Y'P_{\left(X_1 - \bar{X}_1\mathbf{1}\right)}Y$, $SSM_{13} = Y'\tilde{P}_{13}Y = Y'\tilde{\mathbf{X}}_{13}\left(\tilde{\mathbf{X}}_{13}'\tilde{\mathbf{X}}_{13}\right)^{-1}\tilde{\mathbf{X}}_{13}'Y$, and $SSE_{13} = Y'\left(I - P_{13}\right)Y = Y'\left(I - \frac{J}{n}\right)Y - Y'\tilde{P}_{13}Y$.

Matrix $\tilde{\mathbf{X}}_{13} = \left(X_1 - \bar{X}_1\mathbf{1},\ X_3 - \bar{X}_3\mathbf{1},\ X_1X_3 - \overline{X_1X_3}\mathbf{1}\right)$, so that

$$\begin{aligned} \tilde{\mathbf{X}}_{13}'Y &= \left(\left(X_1 - \bar{X}_1\mathbf{1}\right)'Y,\ \left(X_3 - \bar{X}_3\mathbf{1}\right)'Y,\ \left(X_1X_3 - \overline{X_1X_3}\mathbf{1}\right)'Y\right) \\ &= n\begin{pmatrix} b_1\left(\overline{X_1^2} - \overline{X_1}^2\right) + b_2\left(\overline{X_1X_2} - \bar{X}_1\bar{X}_2\right) + b_3\left(\overline{X_1^2X_2} - \bar{X}_1\overline{X_1X_2}\right) + \overline{X_1\varepsilon} - \overline{X_1}\overline{\varepsilon} \\ b_1\left(\overline{X_1X_3} - \bar{X}_1\bar{X}_3\right) + b_2\left(\overline{X_2X_3} - \bar{X}_2\bar{X}_3\right) + b_3\left(\overline{X_1X_2X_3} - \bar{X}_3\overline{X_1X_2}\right) + \overline{X_3\varepsilon} - \overline{X_3}\overline{\varepsilon} \\ b_1\left(\overline{X_1^2X_3} - \bar{X}_1\overline{X_1X_3}\right) + b_2\left(\overline{X_1X_2X_3} - \bar{X}_2\overline{X_1X_3}\right) + b_3\left(\overline{X_1^2X_2X_3} - \left(\overline{X_1X_2}\right)\left(\overline{X_1X_3}\right)\right) + \overline{X_1X_3\varepsilon} - \overline{X_1X_3}\overline{\varepsilon} \end{pmatrix}, \end{aligned}$$

and

$$\tilde{\mathbf{X}}_{13}'\tilde{\mathbf{X}}_{13}=\begin{pmatrix} \overline{X_1^2}-\bar{X}_1^2 & \overline{X_1X_3}-\bar{X}_1\bar{X}_3 & \overline{X_1^2X_3}-\bar{X}_1\overline{X_1X_3} \\ \overline{X_1X_3}-\bar{X}_1\bar{X}_3 & \overline{X_3^2}-\bar{X}_3^2 & \overline{X_1X_3^2}-\bar{X}_3\overline{X_1X_3} \\ \overline{X_1^2X_3}-\bar{X}_1\overline{X_1X_3} & \overline{X_1X_3^2}-\bar{X}_3\overline{X_1X_3} & \overline{X_1^2X_3^2}-\overline{X_1X_3}^2 \end{pmatrix}.$$

So we can rewrite

$$F_{3|1}=(n-4)\,h_{3|1}\left(\bar{\mathbf{Z}}\right),$$

where

$$\mathbf{Z}_i=\begin{pmatrix} \varepsilon_i, X_{i1}, X_{i1}^2, X_{i2}, X_{i2}^2, X_{i1}X_{i2}, X_{i1}^2X_{i2}, X_{i1}\varepsilon_i, X_{i1}X_{i2}^2, X_{i1}^2X_{i2}^2, \varepsilon_i^2, X_{i2}\varepsilon_i, X_{i1}X_{i2}\varepsilon_i, X_{i3}, \\ X_{i3}^2, X_{i3}\varepsilon_i, X_{i1}X_{i3}, X_{i2}X_{i3}, X_{i1}X_{i2}X_{i3}, X_{i1}^2X_{i3}, X_{i1}X_{i3}^2, X_{i1}^2X_{i3}^2, X_{i1}^2X_{i2}X_{i3}, X_{i1}X_{i3}\varepsilon_i \end{pmatrix}.$$

By calculating $\boldsymbol{\theta}=E\left(\mathbf{Z}_i\right)$ and $\Sigma=Var\left(\mathbf{Z}_i\right)$ (analog to the above Supplementary Note Section 2.1, formulas not listed here), we can derive $h_{3|1}\left(\boldsymbol{\theta}\right)=0$ and $\triangledown h_{3|1}\left(\boldsymbol{\theta}\right)=0$. By Taylor expansion and Central Limit Theory,

$$\begin{aligned} F_{3|1} &\rightarrow \frac{1}{2}\left[\sqrt{n-4}\left(\bar{\mathbf{Z}}-\boldsymbol{\theta}\right)\right]' D^2h_{3|1}\left(\boldsymbol{\theta}\right)\left[\sqrt{n-4}\left(\bar{\mathbf{Z}}-\boldsymbol{\theta}\right)\right] \\ &\xrightarrow{L} \frac{1}{2}\left[N\left(0,\Sigma\right)\right]' D^2h_{3|1}\left(\boldsymbol{\theta}\right)\left[N\left(0,\Sigma\right)\right]. \end{aligned}$$

Let $A\equiv D^2h_{3|1}\left(\boldsymbol{\theta}\right)\Sigma$, $rank\left(A\right)=2$. Unfortunately A cannot be idempotent since the two eigenvalues, as two function of genetical parameters, are not equal in general. We note that by S.R. Searle ([Searle(1971)], Chapter 2),

$$\begin{aligned} E\left(F_{3|1}\right) &\rightarrow \frac{1}{2}tr\left(D^2\left(h_{3|1}\left(\boldsymbol{\theta}_{13}\right)\right)\boldsymbol{\Sigma}\right)\equiv e, \\ Var\left(F_{3|1}\right) &\rightarrow \frac{1}{2}tr\left(\left[D^2\left(h_{3|1}\left(\boldsymbol{\theta}_{13}\right)\right)\boldsymbol{\Sigma}\right]^2\right)\equiv v. \end{aligned}$$

So the distribution for $F_{3|1}$ can be approximated by

$$F_{3|1}\overset{(d)}{\cong} c\chi_d^2,$$

where $c=\frac{v}{2e}$, $d=\frac{2e^2}{v}$ ([Scheffé(1959)], Appendix 5). The complicated formula of e and v, as functions of genetical parameters, are given in Supplementary Note Section 3.2.

2.6 Asymptotic distribution for $\left(T_1,T_2,T_{1|3},T_{2|3}\right)'$

$F_{1|3}$ is the F statistic for comparing full epistatic model (1) to single SNP model $\hat{Y}_{i(3)}=\hat{\beta}_{0(3)}+\hat{\beta}_{1(3)}X_{i3}$, measuring how much more variation the full model can explain than the simple model. We can write

$$F_{1|3}=\frac{\left(SSM_{13}-SSM_3\right)/2}{SSE_{13}/\left(n-4\right)},$$

where $SSM_3=Y'P_{\left(X_3-\bar{X}_3\mathbf{1}\right)}Y$ (calculation is given above in Supplementary Note Section 2.2), $SSM_{13}=Y'\tilde{P}_{13}Y=Y'\tilde{\mathbf{X}}_{13}\left(\tilde{\mathbf{X}}_{13}'\tilde{\mathbf{X}}_{13}\right)^{-1}\tilde{\mathbf{X}}_{13}'Y$, and $SSE_{13}=Y'\left(I-P_{13}\right)Y=Y'\left(I-\frac{J}{n}\right)Y-Y'\tilde{P}_{13}Y$. The calculation for SSM_{13} and SSE_{13} is given above in Supplementary Note Section 2.5.

Define

$$T_{1|3}\equiv\sqrt{F_{1|3}}=\sqrt{n-4}h_{1|3}\left(\bar{\mathbf{Z}}\right),$$

by the asymptotic distribution result (5) given in the article, we can derive

$$T_{1|3}-\mu_{T_{1|3}}\xrightarrow{L} N\left(0,\tau_{T_{1|3}}^2\right),$$

where

$$\mu_{T_{1|3}}=\left(\frac{np_1q_1\left(b_1+b_3\left(p_2-q_2\right)\right)^2}{2p_2q_2\left(\left(b_2+b_3p_1\right)^2-2b_2b_3q_1+b_3^2q_1^2\right)+\sigma^2}\right)^{1/2},$$

the formula of $\tau^2_{T_{1|3}}$ is given in Supplementary Note Section 4.3.

Similarly,

$$F_{2|3} = \frac{(SSM_{23} - SSM_3)/2}{SSE_{23}/(n-4)},$$

where $SSM_{23} = Y'\tilde{P}_{23}Y = Y'\tilde{\mathbf{X}}_{23}\left(\tilde{\mathbf{X}}'_{23}\tilde{\mathbf{X}}_{23}\right)^{-1}\tilde{\mathbf{X}}'_{23}Y$, and $SSE_{23} = Y'(I - P_{23})Y = Y'\left(I - \frac{J}{n}\right)Y - Y'\tilde{P}_{23}Y$ with matrix $\tilde{\mathbf{X}}_{23} = \left(X_2 - \bar{X}_2\mathbf{1},\ X_3 - \bar{X}_3\mathbf{1},\ X_2X_3 - \overline{X_2X_3}\mathbf{1}\right)$, so that

$$\begin{aligned}
\tilde{\mathbf{X}}'_{23}Y &= \left(\left(X_2 - \bar{X}_2\mathbf{1}\right)'Y,\ \left(X_3 - \bar{X}_3\mathbf{1}\right)'Y,\ \left(X_2X_3 - \overline{X_2X_3}\mathbf{1}\right)'Y\right) \\
&= n\begin{pmatrix}
b_1\left(\overline{X_1X_2} - \bar{X}_1\bar{X}_2\right) + b_2\left(\overline{X_2^2} - \bar{X}_2^2\right) + b_3\left(\overline{X_1X_2^2} - \bar{X}_2\overline{X_1X_2}\right) + \overline{X_2\varepsilon} - \bar{X}_2\bar{\varepsilon} \\
b_1\left(\overline{X_1X_3} - \bar{X}_1\bar{X}_3\right) + b_2\left(\overline{X_2X_3} - \bar{X}_2\bar{X}_3\right) + b_3\left(\overline{X_1X_2X_3} - \bar{X}_3\overline{X_1X_2}\right) + \overline{X_3\varepsilon} - \bar{X}_3\bar{\varepsilon} \\
b_1\left(\overline{X_1X_2X_3} - \bar{X}_1\overline{X_2X_3}\right) + b_2\left(\overline{X_2^2X_3} - \bar{X}_2\overline{X_2X_3}\right) + b_3\left(\overline{X_1X_2^2X_3} - \left(\overline{X_1X_2}\right)\left(\overline{X_2X_3}\right)\right) + \overline{X_2X_3\varepsilon} - \overline{X_2X_3}\bar{\varepsilon}
\end{pmatrix},
\end{aligned}$$

and

$$\tilde{\mathbf{X}}'_{23}\tilde{\mathbf{X}}_{23} = \begin{pmatrix}
\overline{X_2^2} - \bar{X}_2^2 & \overline{X_2X_3} - \bar{X}_2\bar{X}_3 & \overline{X_2^2X_3} - \bar{X}_2\overline{X_2X_3} \\
\overline{X_2X_3} - \bar{X}_2\bar{X}_3 & \overline{X_3^2} - \bar{X}_3^2 & \overline{X_2X_3^2} - \bar{X}_3\overline{X_2X_3} \\
\overline{X_2^2X_3} - \bar{X}_2\overline{X_2X_3} & \overline{X_2X_3^2} - \bar{X}_3\overline{X_2X_3} & \overline{X_2^2X_3^2} - \overline{X_2X_3}^2
\end{pmatrix}.$$

Define

$$T_{2|3} \equiv \sqrt{F_{2|3}} = \sqrt{n-4}\,h_{2|3}\left(\bar{\mathbf{Z}}\right),$$

by the asymptotic distribution result (5) given in the article, we can derive

$$T_{2|3} - \mu_{T_{2|3}} \xrightarrow{L} N\left(0, \tau^2_{T_{2|3}}\right),$$

where the formulas of $\mu_{T_{2|3}}$ and $\tau^2_{T_{2|3}}$ are analogous to $\mu_{T_{1|3}}$ and $\tau^2_{T_{1|3}}$.

More generally, $\left(T_1, T_2, T^2_{1|3}, T^2_{2|3}\right)'$ has multivariate normal distribution

$$\left(T_1, T_2, T_{1|3}, T_{2|3}\right)' - \boldsymbol{\mu}_{T_1,T_2,T_{1|3},T_{2|3}} \xrightarrow{L} MVN\left(\mathbf{0}, \boldsymbol{\tau}_{T_1,T_2,T_{1|3},T_{2|3}}\right), \tag{2}$$

with

$$\begin{aligned}
\tau_{1|3,2|3} &= Cov\left(T_{2|3}, T_{1|3}\right) \xrightarrow{P} \left[\nabla h_{1|3}(\boldsymbol{\theta})\right]'\Sigma\left[\nabla h_{2|3}(\boldsymbol{\theta})\right], \\
\tau_{1|3,1} &= Cov\left(T_1, T_{1|3}\right) \xrightarrow{P} \left[\nabla h_{1|3}(\boldsymbol{\theta})\right]'\Sigma\left[\nabla h_1(\boldsymbol{\theta})\right], \\
\tau_{1|3,2} &= Cov\left(T_2, T_{1|3}\right) \xrightarrow{P} \left[\nabla h_{1|3}(\boldsymbol{\theta})\right]'\Sigma\left[\nabla h_2(\boldsymbol{\theta})\right], \\
\tau_{2|3,1} &= Cov\left(T_1, T_{2|3}\right) \xrightarrow{P} \left[\nabla h_{2|3}(\boldsymbol{\theta})\right]'\Sigma\left[\nabla h_1(\boldsymbol{\theta})\right], \\
\tau_{2|3,2} &= Cov\left(T_2, T_{2|3}\right) \xrightarrow{P} \left[\nabla h_{2|3}(\boldsymbol{\theta})\right]'\Sigma\left[\nabla h_2(\boldsymbol{\theta})\right],
\end{aligned}$$

where $h_1(\boldsymbol{\theta})$ and $h_2(\boldsymbol{\theta})$ are given in the above Supplementary Note Section 2.1. The formulas of $\tau_{1|3,1}$, $\tau_{1|3,2}$, and $\tau_{1|3,2|3}$ are given in Supplementary Note Section 4.3.

Furthermore, we have

$$Cov\left(T_3, T_{1|3}\right) \xrightarrow{P} \left[\nabla h_{1|3}(\boldsymbol{\theta})\right]'\Sigma\left[\nabla h_3(\boldsymbol{\theta})\right] = 0.$$

As T_3 and $T_{1|3}$ are normal, they are independent. So T_3 and $F_{1|3}$ are also independent.

2.7 Asymptotic distribution for $F_{4|3}$

The asymptotic distribution of $F_{4|3}$ is calculated as following.

$$F_{4|3} = \frac{(SSM_{34} - SSM_3)/2}{SSE_{34}/(n-4)},$$

where $SSM_3 = Y'P_{\left(X_3 - \bar{X}_3\mathbf{1}\right)}Y$ is given above in Supplementary Note Section 2.2, $SSM_{34} = Y'\left(P_{34} - \frac{J}{n}\right)Y$, $SSE_{34} = Y'\left(I - P_{34}\right)Y$ are given above in Supplementary Note Section 2.4.

We can rewrite

$$F_{4|3} = (n-4)\, h_{4|3}\left(\bar{\mathbf{Z}}\right),$$

where $\mathbf{Z}_i$ is the same as given above in Supplementary Note Section 2.4. We get that $h_{4|3}\left(\boldsymbol{\theta}\right) = 0$ and the vector of eigenvalues of $D^2 h\left(\boldsymbol{\theta}\right)\Sigma$ is $(1, 1, 0, ...0)$. This indicates that $D^2 h\left(\boldsymbol{\theta}\right)\Sigma$ is idempotent. By the asymptotic distribution result (7) in the article,

$$\begin{aligned} F_{4|3} &= (n-4)\, h\left(\bar{\mathbf{Z}}\right) \\ &\stackrel{L}{\to} \frac{1}{2}\left[N\left(0, \Sigma\right)\right]' D^2 h\left(\boldsymbol{\theta}\right)\left[N\left(0, \Sigma\right)\right] \\ &\stackrel{(d)}{=} \frac{1}{2}\chi^2_2. \end{aligned}$$

References

[David(1981)] H. David. Order Statistics. *New York*, 1981.

[Rawlings(1976)] J. Rawlings. Order statistics for a special class of unequally correlated multinormal variates. *Biometrics*, 32(4):875–887, 1976.

[Scheffé(1959)] H. Scheffé. *The Analysis of Variance*. John Wiley & Sons Inc, 1959.

[Searle(1971)] S. Searle. Linear Models. *New York*, 1971.

[Wolfram(1999)] S. Wolfram. *The Mathematica Book*. Cambridge University Press, 1999.

3 Comparison of power for model selection methods

Assume the true genetic model is a two-marker epistatic model given in equation (2) in the article. Setting the number of observations $n = 100$, the number of scanned SNPs $p = 1000$, and the variance of random error $\sigma^2 = 3$, the following figures demonstrate values and comparisons of power for marginal selection, forward selection, and exhaustive search while controlling the number of false discoveries at $R = 1$, 5, and 10. Two minor allele frequencies are studied: $q_j = 0.5$ (Figures S1 - S4) and 0.3 (Figures S5 - S8). Genetic effects ($b_1 = b_2$ and b_3) vary from -2 to 2 by a step size of 0.2. Power values of model selection methods are calculated and compared under two definitions: (A) finding the exact true underlying genetic model (or both SNPs in the case of marginal selection) (Figures S1, S3, S5, S7); (B): finding at least one of the true underlying SNPs (Figures S2, S4, S6, S8).

Figure S1. 3D plots of power under definition (A), $q_j = 0.5$, $R = 1, 5, 10$ (row 1-3).

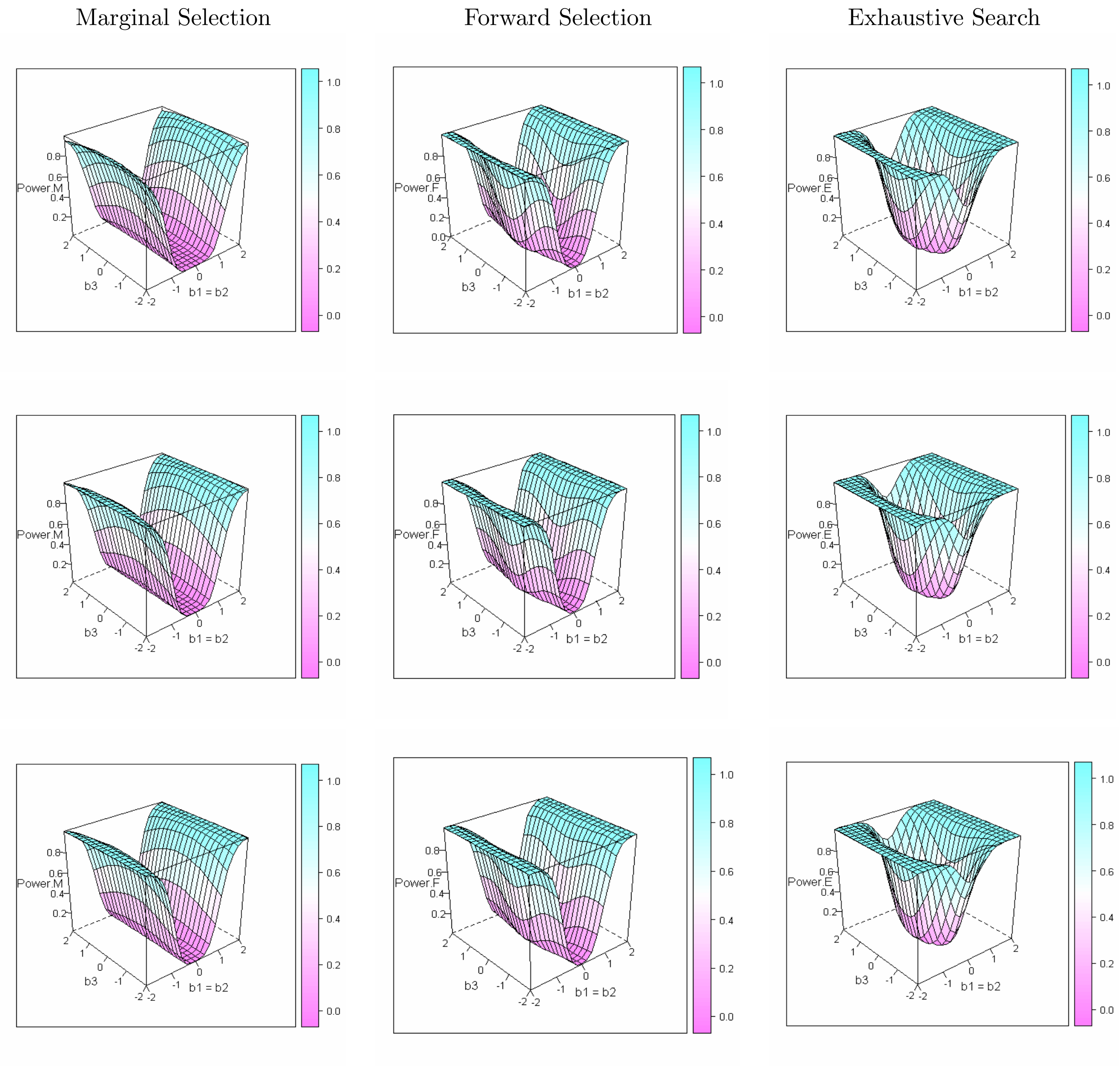

Figure S2. 3D plots of power under definition (B), $q_j = 0.5$, $R = 1, 5, 10$ (row 1-3).

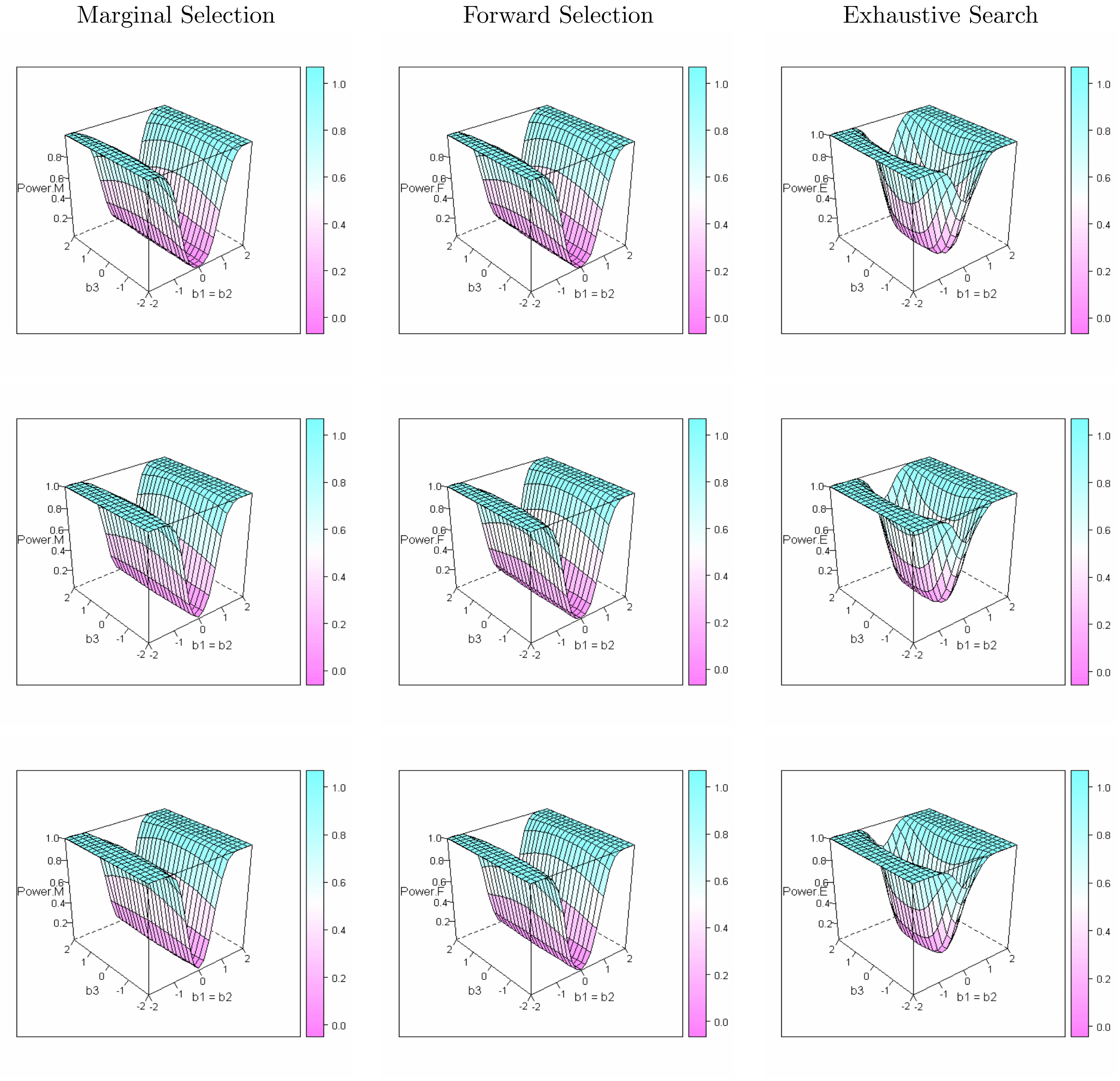

Figure S3. Power comparisons under definition (A), $q_j = 0.5$, $R = 1, 5, 10$ (row 1-3).

Marginal - Exhaustive | Forward - Exhaustive | Marginal –Forward

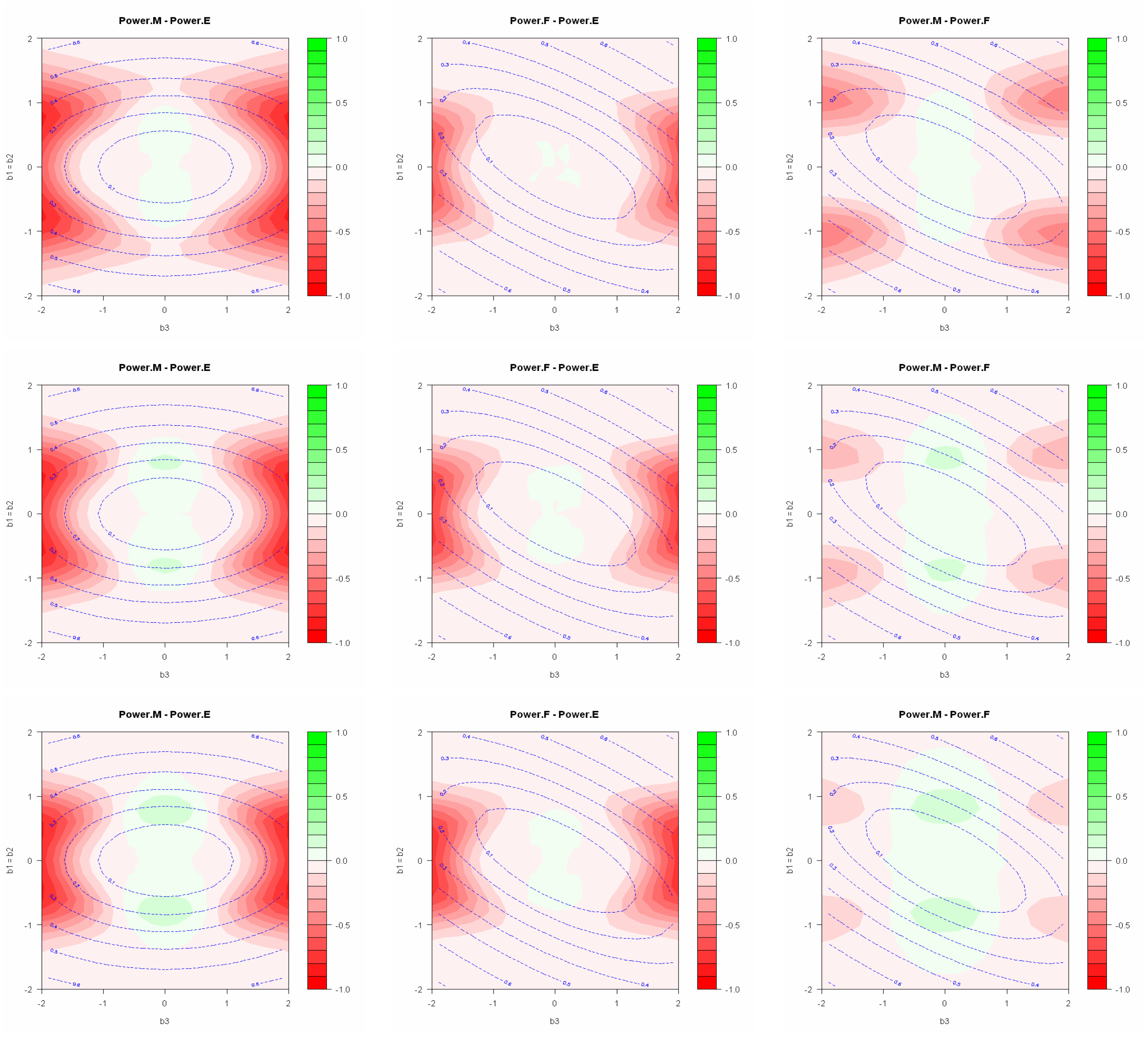

Figure S4. Power comparisons under definition (B), $q_j = 0.5$, $R = 1, 5, 10$ *(row 1-3).*

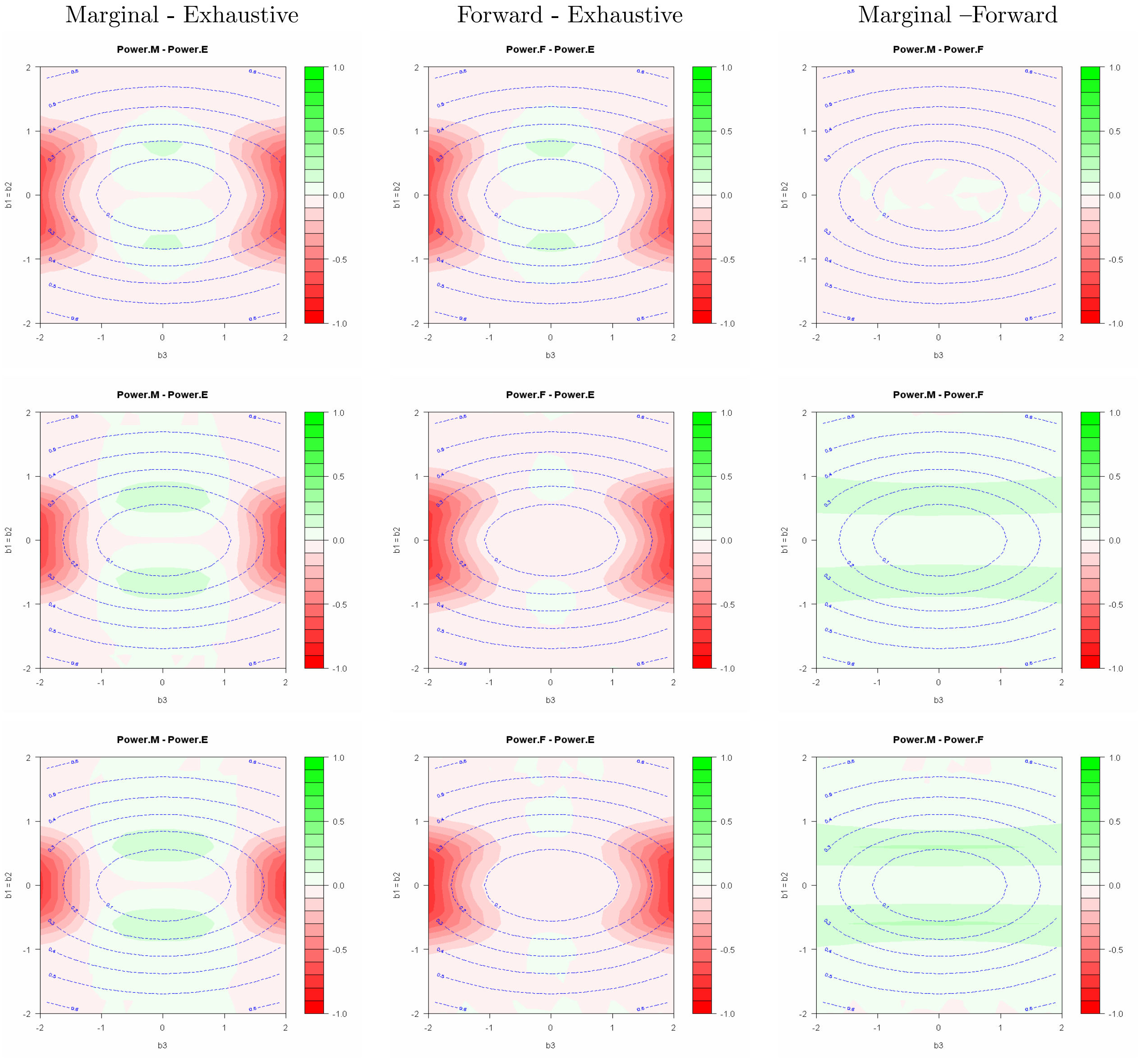

Figure S5. 3D plots of power under definition (A), $q_j = 0.3$, $R = 1, 5, 10$ (row 1-3).

Marginal Selection | Forward Selection | Exhaustive Search

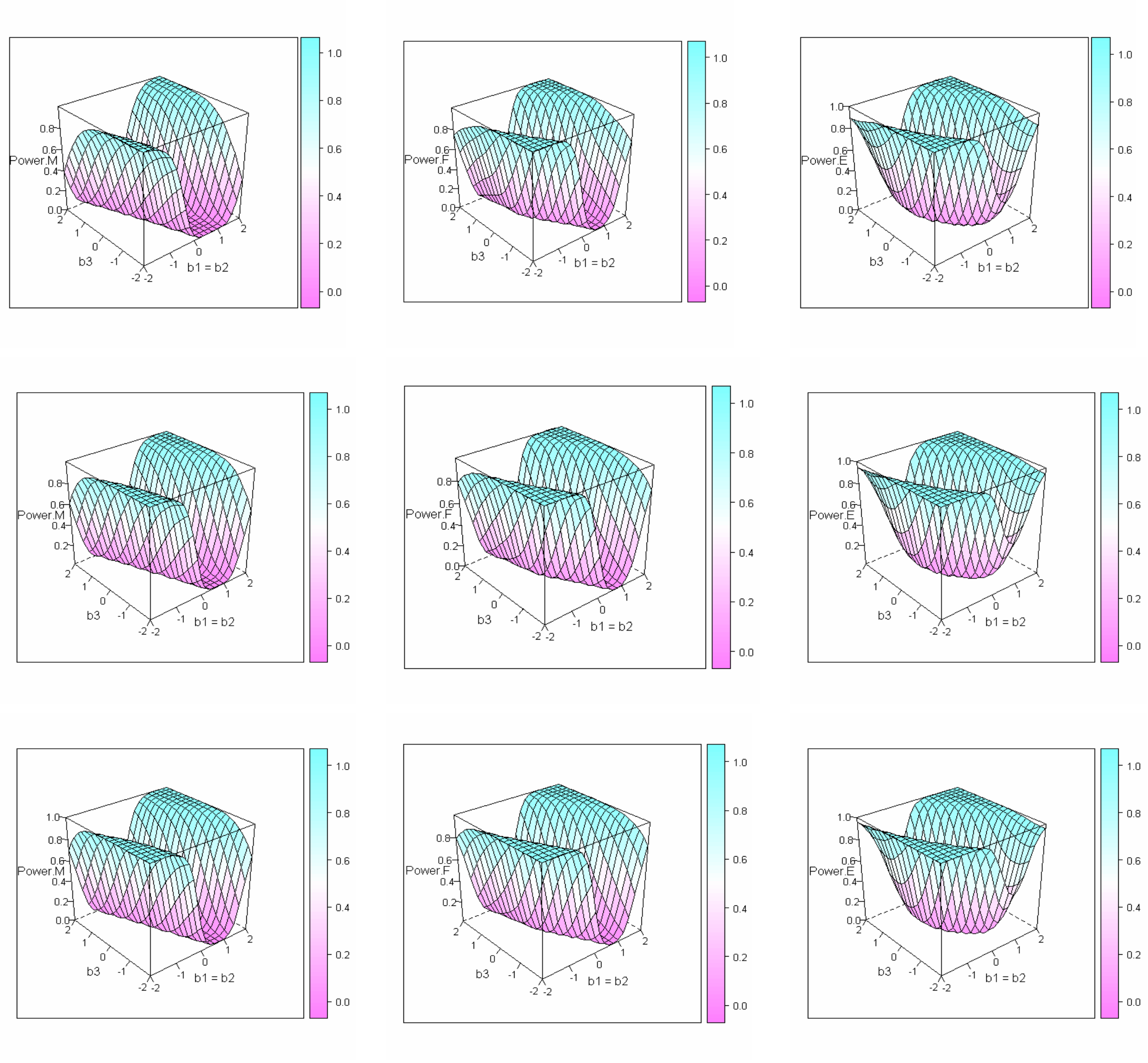

Figure S6. 3D plots of powers under definition (B), $q_j = 0.3$, $R = 1, 5, 10$ (row 1-3).

Marginal Selection | Forward Selection | Exhaustive Search

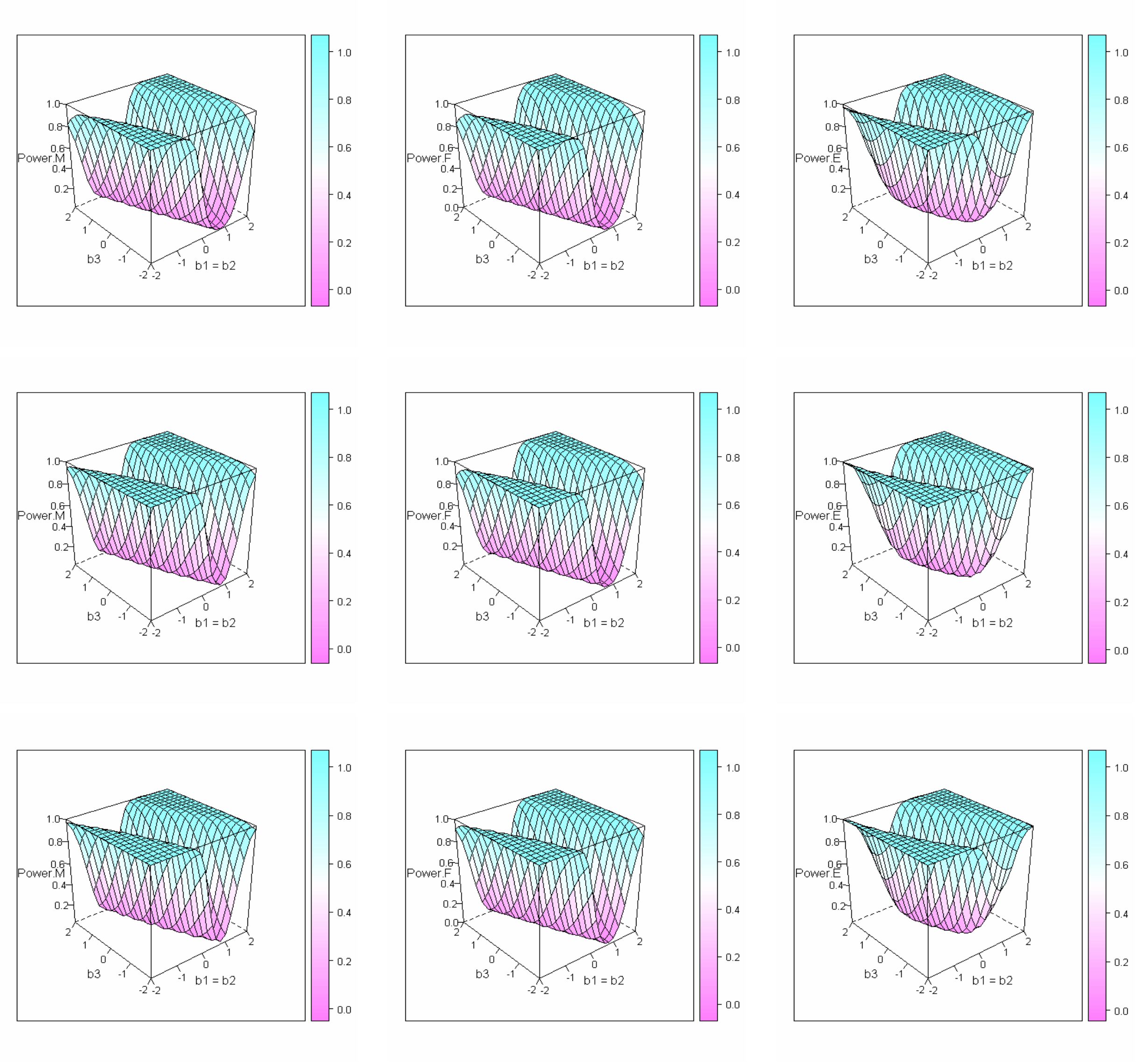

Figure S7. Power comparisons under definition (A), $q_j = 0.3$, $R = 1, 5, 10$ (row 1-3).

Marginal - Exhaustive | Forward - Exhaustive | Marginal –Forward

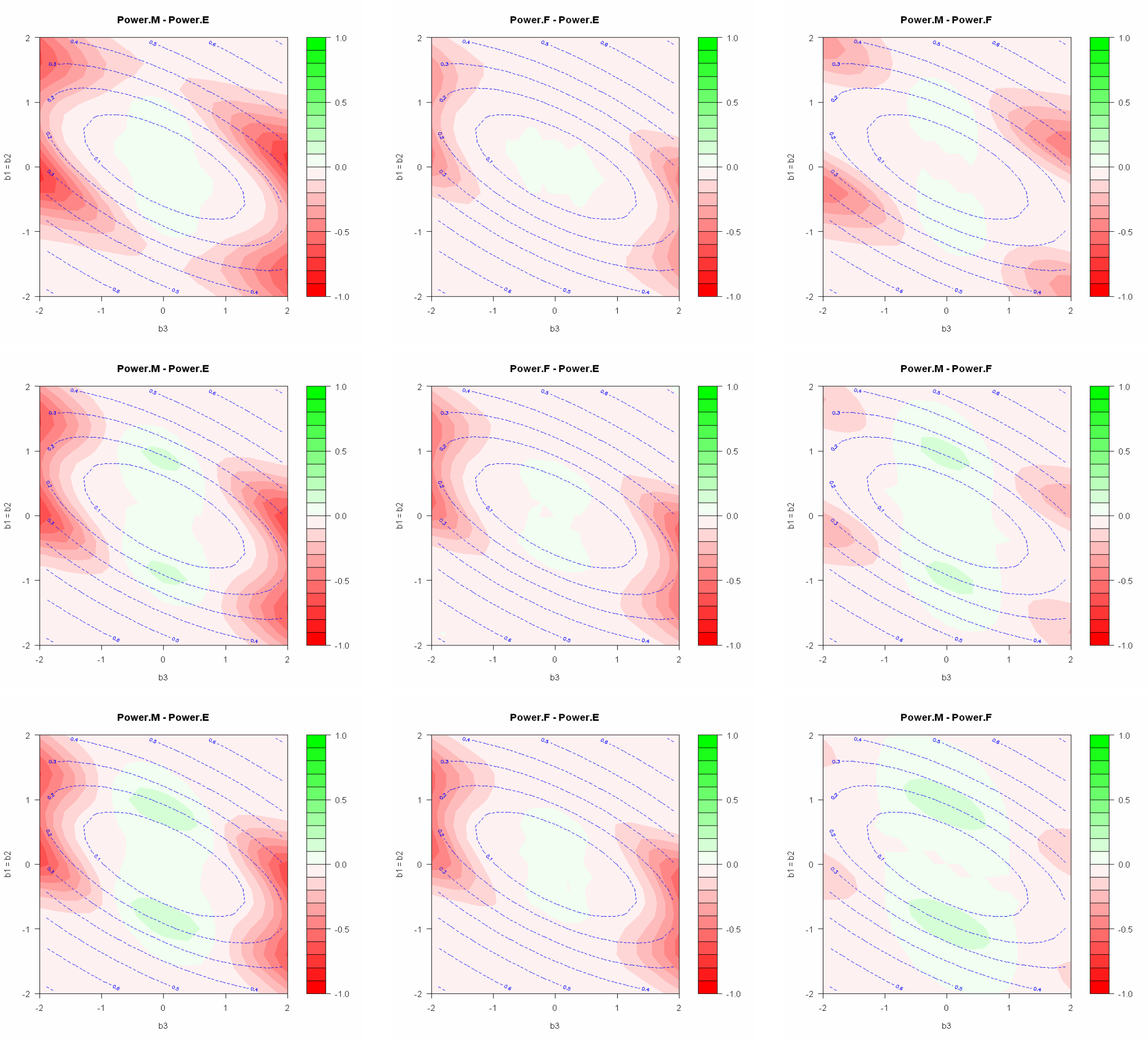

Figure S8. Power comparisons under definition (B), $q_j = 0.3$, $R = 1, 5, 10$ *(row 1-3).*

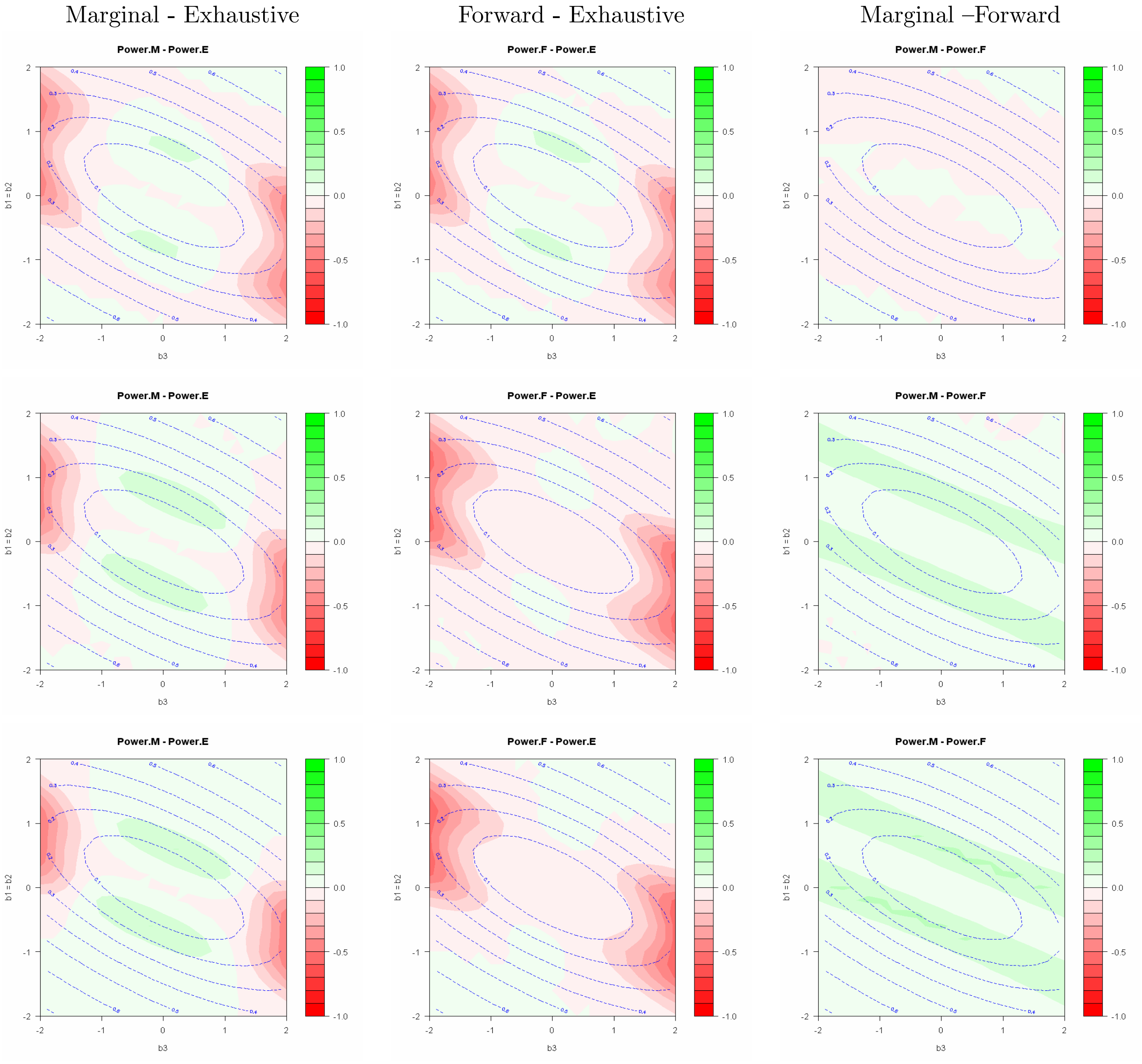

4 Formulas for distribution parameters of test statistics

The following formulas of distribution parameters are estimated based on genetic setup described in article equation (1).

4.1 Parameters for (T_{12}, T_1, T_2)

$E(T_1) \to \sqrt{n} h_1 \left(\bar{\mathbf{Z}}\right) =$

sqrt(n)*
(sqrt(B)*sqrt(n)*p1*q1*(b1 + b3*(p2 - q2)))/
sqrt(p1*q1*(2*p2*((b2 + b3*p1)^2 - 2*b2*b3*q1 + b3^2*q1^2)*q2 + sigm^2))

$Var(T_1) = \tau_1^2 \to [\nabla h_1(\boldsymbol{\theta})]' \Sigma [\nabla h_2(\boldsymbol{\theta})] =$

(-6*b3^6*p1^4*p2^4*q1^2 + 8*b3^6*p1^4*p2^6*q1^2 - 2*b3^6*p1^6*p2^6*q1^2 -
4*b3^6*p1^5*p2^6*q1^3 - 6*b3^6*p1^2*p2^4*q1^4 + 8*b3^6*p1^2*p2^6*q1^4 -
4*b3^6*p1^4*p2^6*q1^4 - 4*b3^6*p1^3*p2^6*q1^5 - 2*b3^6*p1^2*p2^6*q1^6 +
12*b3^6*p1^4*p2^3*q1^2*q2 + 4*b3^6*p1^6*p2^5*q1^2*q2 +
8*b3^6*p1^5*p2^5*q1^3*q2 + 12*b3^6*p1^2*p2^3*q1^4*q2 +
8*b3^6*p1^4*p2^5*q1^4*q2 + 8*b3^6*p1^3*p2^5*q1^5*q2 +
4*b3^6*p1^2*p2^5*q1^6*q2 - 8*b3^6*p1^6*p2^4*q2^2 + 8*b3^6*p1^8*p2^4*q2^2 +
32*b3^6*p1^5*p2^4*q1*q2^2 - 12*b3^6*p1^7*p2^4*q1*q2^2 -
12*b3^6*p1^4*p2^2*q1^2*q2^2 - 32*b3^6*p1^4*p2^4*q1^2*q2^2 +
10*b3^6*p1^6*p2^4*q1^2*q2^2 + 64*b3^6*p1^3*p2^4*q1^3*q2^2 -
32*b3^6*p1^5*p2^4*q1^3*q2^2 - 12*b3^6*p1^2*p2^2*q1^4*q2^2 -
32*b3^6*p1^2*p2^4*q1^4*q2^2 + 4*b3^6*p1^4*p2^4*q1^4*q2^2 +
32*b3^6*p1*p2^4*q1^5*q2^2 - 32*b3^6*p1^3*p2^4*q1^5*q2^2 -
8*b3^6*p2^4*q1^6*q2^2 + 10*b3^6*p1^2*p2^4*q1^6*q2^2 -
12*b3^6*p1*p2^4*q1^7*q2^2 + 8*b3^6*p2^4*q1^8*q2^2 +
32*b2^6*p1*p2^3*q1*q2^3 - 8*b3^6*p1^7*p2^3*q1*q2^3 +
12*b3^6*p1^4*p2*q1^2*q2^3 + 40*b3^6*p1^6*p2^3*q1^2*q2^3 -
40*b3^6*p1^5*p2^3*q1^3*q2^3 + 12*b3^6*p1^2*p2*q1^4*q2^3 +
80*b3^6*p1^4*p2^3*q1^4*q2^3 - 40*b3^6*p1^3*p2^3*q1^5*q2^3 +
40*b3^6*p1^2*p2^3*q1^6*q2^3 - 8*b3^6*p1*p2^3*q1^7*q2^3 -
8*b3^6*p1^6*p2^2*q2^4 + 8*b3^6*p1^8*p2^2*q2^4 + 32*b3^6*p1^5*p2^2*q1*q2^4 -
12*b3^6*p1^7*p2^2*q1*q2^4 - 6*b3^6*p1^4*q1^2*q2^4 -
32*b3^6*p1^4*p2^2*q1^2*q2^4 + 10*b3^6*p1^6*p2^2*q1^2*q2^4 +
64*b3^6*p1^3*p2^2*q1^3*q2^4 - 32*b3^6*p1^5*p2^2*q1^3*q2^4 -
6*b3^6*p1^2*q1^4*q2^4 - 32*b3^6*p1^2*p2^2*q1^4*q2^4 +
4*b3^6*p1^4*p2^2*q1^4*q2^4 + 32*b3^6*p1*p2^2*q1^5*q2^4 -
32*b3^6*p1^3*p2^2*q1^5*q2^4 - 8*b3^6*p2^2*q1^6*q2^4 +
10*b3^6*p1^2*p2^2*q1^6*q2^4 - 12*b3^6*p1*p2^2*q1^7*q2^4 +
8*b3^6*p2^2*q1^8*q2^4 + 4*b3^6*p1^6*p2*q1^2*q2^5 +
8*b3^6*p1^5*p2*q1^3*q2^5 + 8*b3^6*p1^4*p2*q1^4*q2^5 +
8*b3^6*p1^3*p2*q1^5*q2^5 + 4*b3^6*p1^2*p2*q1^6*q2^5 +
8*b3^6*p1^4*q1^2*q2^6 - 2*b3^6*p1^6*q1^2*q2^6 - 4*b3^6*p1^5*q1^3*q2^6 +
8*b3^6*p1^2*q1^4*q2^6 - 4*b3^6*p1^4*q1^4*q2^6 - 4*b3^6*p1^3*q1^5*q2^6 -
2*b3^6*p1^2*q1^6*q2^6 - 16*b2^5*b3*p2^2*(p1 - q1)*q2^2*
(p2^2*(-1 + p1 + q1)*(1 + p1 + q1) - 8*p1*p2*q1*q2 +
(-1 + p1 + q1)*(1 + p1 + q1)*q2^2) +
4*b3^4*(p1^5*p2*q1*q2*(-3*p2^2 + 2*p2*q2 - 3*q2^2) +
2*p1^6*p2*q2*(p2^2 + q2^2) + 2*p2*q1^4*(-1 + q1^2)*q2*(p2^2 + q2^2) +
p1*p2*q1^3*q2*(p2^2*(8 - 3*q1^2) + 2*p2*q1^2*q2 + (8 - 3*q1^2)*q2^2) +
2*p1^3*q1*(-2*p2^4*q1^2 + p2^3*(4 + 5*q1^2)*q2 - 10*p2^2*q1^2*q2^2 +
p2*(4 + 5*q1^2)*q2^3 - 2*q1^2*q2^4) -
2*p1^4*(p2^4*q1^2 + p2*(1 - 4*q1^2)*q2^3 + q1^2*q2^4 +
p2^3*(q2 - 4*q1^2*q2)) - 2*p1^2*q1^2*(p2^4*(-2 + q1^2) +
p2^3*(6 - 4*q1^2)*q2 + q2^2 + (-2 + q1^2)*q2^4 + p2^2*(1 - 4*q2^2) -

```
2*p2*(q2 + (-3 + 2*q1^2)*q2^3)))*sigm^2 +
b3^2*(p2^2*(2*p1^4 - 3*p1^3*q1 + 2*q1^2*(-1 + q1^2) - 2*p1^2*(1 + 4*q1^2) +
p1*(8*q1 - 3*q1^3)) + 2*p1*p2*q1*(7*p1^2 + 12*p1*q1 + 7*q1^2)*q2 +
(2*p1^4 - 3*p1^3*q1 + 2*q1^2*(-1 + q1^2) - 2*p1^2*(1 + 4*q1^2) +
p1*(8*q1 - 3*q1^3))*q2^2)*sigm^4 + 4*p1*q1*sigm^6 +
4*b2^4*p2*q2*(b3^2*(-14*p1^4*p2*q2*(p2^2 + q2^2) -
14*p2*q1^2*(-1 + q1^2)*q2*(p2^2 + q2^2) +
p1^2*(p2^4*q1^2 - 78*p2^2*q1^2*q2^2 + 14*p2*(1 + 3*q1^2)*q2^3 +
q1^2*q2^4 + 14*p2^3*(q2 + 3*q1^2*q2)) +
p1*(4*p2^4*q1^3 + 8*p2*q1^3*q2 - 3*p2^3*q1*(8 + q1^2)*q2 -
3*p2*q1*(8 + q1^2)*q2^3 + 4*q1^3*q2^2*(-1 + q2^2) +
2*p2^2*q1^3*(-2 + 19*q2^2)) + p1^3*q1*(4*p2^4 - 3*p2^3*q2 +
4*q2^2*(-1 + q2^2) + p2^2*(-4 + 38*q2^2) + p2*(8*q2 - 3*q2^3))) +
12*p1*p2*q1*q2*sigm^2) - 8*b2^3*b3*p2*(p1 - q1)*q2*
(b3^2*(p1*p2^2*q1*(3 + p1^2*(2 + p2^2) + 4*p1*p2^2*q1 + 2*q1^2 +
p2^2*(-6 + q1^2)) + 2*p2*(4*p1^4*p2^2 + p1^3*(-2 + p2^2)*q1 +
4*p2^2*q1^2*(-1 + q1^2) - 2*p1^2*p2^2*(2 + 11*q1^2) +
p1*q1*(-3 - 2*q1^2 + p2^2*(6 + q1^2)))*q2 +
p1*q1*(3 + 2*p1^2*(1 + 5*p2^2) + 48*p1*p2^2*q1 + 2*q1^2 +
2*p2^2*(-6 + 5*q1^2))*q2^2 + 2*p2*(4*p1^4 + p1^3*q1 +
4*q1^2*(-1 + q1^2) + p1*q1*(6 + q1^2) - 2*p1^2*(2 + 11*q1^2))*q2^3 +
p1*q1*(-6 + p1^2 + 4*p1*q1 + q1^2)*q2^4) +
2*(p2^2*(-1 + p1 + q1)*(1 + p1 + q1) - 8*p1*p2*q1*q2 +
(-1 + p1 + q1)*(1 + p1 + q1)*q2^2)*sigm^2) -
4*b2^2*(2*b3^4*(2*p1^6*p2^2*q2^2*(p2^2 + q2^2) + 2*p2^2*q1^4*(-1 + q1^2)*
q2^2*(p2^2 + q2^2) + p1^5*p2*q1*q2*(8*p2^4 - 7*p2^3*q2 - 2*q2^2 +
8*q2^4 + p2^2*(-2 + 34*q2^2) + p2*(4*q2 - 7*q2^3)) -
p1^4*(3*p2^6*q1^2 + p2^5*q1^2*q2 - 2*p2^3*q1^2*q2*(-3 + q2^2) +
3*q1^2*q2^4*(-1 + q2^2) + p2*q1^2*q2^3*(6 + q2^2) +
p2^4*(-3*q1^2 + (2 + 39*q1^2)*q2^2) +
p2^2*(-6*q1^2*q2^2 + (2 + 39*q1^2)*q2^4)) +
p1^2*q1^2*(-3*p2^6*q1^2 - p2^5*(-20 + q1^2)*q2 -
3*q1^2*q2^4*(-1 + q2^2) + 3*p2^4*(q1^2 - (4 + 13*q1^2)*q2^2) +
p2^2*q2^2*(20 - 12*q2^2 + q1^2*(6 - 39*q2^2)) +
2*p2^3*q2*(-5 + 20*q2^2 + q1^2*(-3 + q2^2)) -
p2*q2^3*(10 - 20*q2^2 + q1^2*(6 + q2^2))) +
2*p1^3*p2*q1*q2*(-2*p2^4*(3 + q1^2) + p2^3*(4 + 29*q1^2)*q2 +
q2^2*(3 - 2*q1^2 - 2*(3 + q1^2)*q2^2) -
p2^2*(-3 + 2*q1^2 + 6*(2 + 3*q1^2)*q2^2) +
p2*q2*(-6 + 4*q2^2 + q1^2*(4 + 29*q2^2))) +
p1*p2*q1^3*q2*(4*p2^4*(-3 + 2*q1^2) + p2^3*(8 - 7*q1^2)*q2 -
2*(-3 + q1^2)*q2^2 + 4*(-3 + 2*q1^2)*q2^4 +
p2*q2*(-12 + 8*q2^2 + q1^2*(4 - 7*q2^2)) +
p2^2*(6 - 24*q2^2 + q1^2*(-2 + 34*q2^2)))) +
b3^2*(6*p1^4*p2*q2*(p2^2 + q2^2) + 6*p2*q1^2*(-1 + q1^2)*q2*
(p2^2 + q2^2) - 2*p1^2*p2*q2*(p2^2*(3 + 10*q1^2) - 16*p2*q1^2*q2 +
(3 + 10*q1^2)*q2^2) + p1*(-2*p2^4*q1^3 - 4*p2*q1^3*q2 +
p2^3*q1*(8 + 3*q1^2)*q2 + p2*q1*(8 + 3*q1^2)*q2^3 +
2*p2^2*q1^3*(1 - 13*q2^2) - 2*q1^3*q2^2*(-1 + q2^2)) +
p1^3*q1*(-2*p2^4 + 3*p2^3*q2 + 2*q2^2 - 2*q2^4 + p2^2*(2 - 26*q2^2) +
p2*q2*(-4 + 3*q2^2)))*sigm^2 - 6*p1*p2*q1*q2*sigm^4) +
4*b2*b3*(p1 - q1)*(2*b3^4*(2*p1^6*p2^2*q2^2*(p2^2 + q2^2) +
2*p2^2*q1^4*(-1 + q1^2)*q2^2*(p2^2 + q2^2) - p1^4*(p2^2 + q2^2)*
(p2^4*q1^2 - 2*p2^2*(-1 + 4*q1^2)*q2^2 + q1^2*q2^4) +
p1^2*q1^2*(-(p2^4*(3 + p2^2*(-4 + q1^2))) - 2*p2^3*(-5 + 4*p2^2)*q2 +
p2^2*(-14 + p2^2*(8 + 7*q1^2))*q2^2 - 2*p2*(-5 + 8*p2^2)*q2^3 +
```

```
(-3 + p2^2*(8 + 7*q1^2))*q2^4 - 8*p2*q2^5 - (-4 + q1^2)*q2^6) +
p1^5*p2*q1*q2*(-5*p2^4 + 2*p2^3*q2 + 2*q2^2 - 5*q2^4 +
p2^2*(2 - 18*q2^2) + 2*p2*q2*(-2 + q2^2)) +
p1*p2*q1^3*q2*(p2^4*(6 - 5*q1^2) + 2*p2^3*(2 + q1^2)*q2 +
2*p2*q2*(3 - 2*q1^2 + (2 + q1^2)*q2^2) +
p2^2*(-3 + 2*q1^2 - 6*(-2 + 3*q1^2)*q2^2) +
q2^2*(-3 + 6*q2^2 + q1^2*(2 - 5*q2^2))) +
p1^3*q1*(-2*p2^6*q1^2 + 2*p2^5*(3 + q1^2)*q2 - 2*p2^4*(-2 + 13*q1^2)*
q2^2 - 2*q1^2*q2^6 + p2*q2^3*(-3 + 4*q1^2 + 2*(3 + q1^2)*q2^2) +
p2^3*q2*(-3 + 4*q1^2 + 4*(3 + q1^2)*q2^2) +
2*p2^2*q2^2*(3 + 2*q2^2 - q1^2*(4 + 13*q2^2)))) -
b3^2*p1*q1*(p2^4*(-6 + 5*p1^2 + 6*p1*q1 + 5*q1^2) +
2*p2^3*(-2 + p1^2 - 8*p1*q1 + q1^2)*q2 +
2*p2*q2*(-3 + 2*p1^2 + 2*q1^2 + (-2 + p1^2 - 8*p1*q1 + q1^2)*q2^2) +
p2^2*(3 - 2*p1^2 - 2*q1^2 + 2*(-6 + p1^2 + 10*p1*q1 + q1^2)*q2^2) +
q2^2*(3 - 2*p1^2 - 2*q1^2 + (-6 + 5*p1^2 + 6*p1*q1 + 5*q1^2)*q2^2))*
sigm^2 - (p2^2*(-1 + p1 + q1)*(1 + p1 + q1) - 8*p1*p2*q1*q2 +
(-1 + p1 + q1)*(1 + p1 + q1)*q2^2)*sigm^4) +
b1^2*p1*q1*(4*b2^4*p2*q2*(p2*q1 + p1*q2)*(p1*p2 + q1*q2) +
2*b3^4*(p1^2 + q1^2)*
(-(p1*p2^2*q1*(3 + p2^2*(-2 + p1 + q1)*(2 + p1 + q1))) +
8*p1*p2*q1*(1 + p2^2*(-1 + p1 + q1)*(1 + p1 + q1))*q2 +
(2*p1^4*p2^2 - 10*p1^3*p2^2*q1 - 56*p1^2*p2^2*q1^2 + 2*p2^2*q1^4 +
p1*q1*(-3 + 2*p2^2*(4 - 5*q1^2)))*q2^2 + 8*p1*p2*q1*(-1 + p1 + q1)*
(1 + p1 + q1)*q2^3 - p1*q1*(-2 + p1 + q1)*(2 + p1 + q1)*q2^4) +
16*b2^3*b3*p2*(p1 - q1)*q2*(p1^2*p2*q2 + p2*q1^2*q2 +
p1*q1*(p2 + q2)^2) + 4*b3^2*p2*(p1^4 + 6*p1^3*q1 - 6*p1^2*q1^2 +
6*p1*q1^3 + q1^4)*q2*sigm^2 + (p1^2 + 4*p1*q1 + q1^2)*sigm^4 +
4*b2^2*(2*b3^2*(3*p1*p2^2*(-1 + p2^2)*q1*(p1^2 + q1^2) +
p1*p2*(8 + p2^2)*q1*(p1^2 + q1^2)*q2 +
(3*p1^4*p2^2 - p1^3*(3 + 4*p2^2)*q1 - 10*p1^2*p2^2*q1^2 -
p1*(3 + 4*p2^2)*q1^3 + 3*p2^2*q1^4)*q2^2 + p1*p2*q1*(p1^2 + q1^2)*
q2^3 + 3*p1*q1*(p1^2 + q1^2)*q2^4) + p2*(p1^2 + 4*p1*q1 + q1^2)*q2*
sigm^2) + 8*b2*b3*(p1 - q1)*
(b3^2*(-(p1*p2^2*q1*(3 + p2^2*(-2 + p1 + q1)*(2 + p1 + q1))) +
8*p1*p2*q1*(1 + p2^2*(-1 + p1 + q1)*(1 + p1 + q1))*q2 +
(2*p1^4*p2^2 - 10*p1^3*p2^2*q1 - 40*p1^2*p2^2*q1^2 + 2*p2^2*q1^4 +
p1*q1*(-3 + 2*p2^2*(4 - 5*q1^2)))*q2^2 + 8*p1*p2*q1*(-1 + p1 + q1)*
(1 + p1 + q1)*q2^3 - p1*q1*(-2 + p1 + q1)*(2 + p1 + q1)*q2^4) +
p2*(p1^2 + 6*p1*q1 + q1^2)*q2*sigm^2)) +
2*b1*b3*p1*q1*(p2 - q2)*(2*b3^4*(p1^2 + q1^2)*
(-(p1*p2^2*q1*(3 + p2^2*(-2 + p1 + q1)*(2 + p1 + q1))) +
4*p1*p2*q1*(1 + p2^2*(p1 + q1)^2)*q2 +
(2*p1^4*p2^2 + 6*p1^3*p2^2*q1 - 24*p1^2*p2^2*q1^2 + 2*p2^2*q1^4 +
p1*q1*(-3 + p2^2*(8 + 6*q1^2)))*q2^2 + 4*p1*p2*q1*(p1 + q1)^2*q2^3 -
p1*q1*(-2 + p1 + q1)*(2 + p1 + q1)*q2^4) - 4*b2^3*b3*p2*(p1 - q1)*q2*
(3 - 6*p2^2 - 6*q2^2 + 2*p1*q1*(p2^2 - 28*p2*q2 + q2^2) +
p1^2*(2 + p2^2 - 14*p2*q2 + q2^2) + q1^2*(2 + p2^2 - 14*p2*q2 +
q2^2)) + 4*b2^4*p2*q2*(p1*q1*(p2^2 + 12*p2*q2 + q2^2) +
p1^2*(-2 + (2*p2 + q2)*(p2 + 2*q2)) +
q1^2*(-2 + (2*p2 + q2)*(p2 + 2*q2))) +
4*b3^2*(-(p1*q1*(1 + p2^2*(-2 + (p1 + q1)^2))) +
p2*(p1^2 + q1^2)*(p1^2 + 10*p1*q1 + q1^2)*q2 - p1*q1*(-2 + (p1 + q1)^2)*
q2^2)*sigm^2 + (p1^2 + 4*p1*q1 + q1^2)*sigm^4 -
4*b2^2*(2*b3^2*(p1^4*p2*q2*(-1 + 4*p2^2 - 7*p2*q2 + 4*q2^2) +
p1^2*p2*q2*(3 - 2*q1^2 - 2*p2^2*(3 + q1^2) + 42*p2*q1^2*q2 -
```

2*(3 + q1^2)*q2^2) + p2*q1^2*q2*(3 + p2^2*(-6 + 4*q1^2) -
7*p2*q1^2*q2 - 6*q2^2 + q1^2*(-1 + 4*q2^2)) -
p1^3*q1*(3*p2^4 + 4*p2^3*q2 + 3*q2^2*(-1 + q2^2) +
p2^2*(-3 + 26*q2^2) + 4*p2*(q2 + q2^3)) +
p1*(-3*p2^4*q1^3 + 2*p2^3*q1*(5 - 2*q1^2)*q2 +
p2^2*q1^3*(3 - 26*q2^2) - 3*q1^3*q2^2*(-1 + q2^2) -
p2*q1*q2*(5 - 10*q2^2 + 4*q1^2*(1 + q2^2)))) -
((-1 + p2^2)*(p1^2 + q1^2) + p2*(3*p1 + q1)*(p1 + 3*q1)*q2 +
(p1^2 + q1^2)*q2^2)*sigm^2) - 2*b2*b3*(p1 - q1)*
(2*b3^2*(2*p1*p2^2*q1*(3 + p2^2*(-2 + p1 + q1)*(2 + p1 + q1)) +
p2*(p1^4*(-2 + 5*p2^2) - 6*p1^3*p2^2*q1 -
2*p1*q1*(6 + p2^2*(-4 + 3*q1^2)) + q1^2*(3 - 2*q1^2 +
p2^2*(-6 + 5*q1^2)) - p1^2*(-3 + 4*q1^2 + 2*p2^2*(3 + 7*q1^2)))*
q2 - 2*(3*p1^4*p2^2 + 10*p1^3*p2^2*q1 - 22*p1^2*p2^2*q1^2 +
3*p2^2*q1^4 + p1*q1*(-3 + 2*p2^2*(4 + 5*q1^2)))*q2^2 +
p2*(5*p1^4 - 6*p1^3*q1 + q1^2*(-6 + 5*q1^2) - 2*p1^2*(3 + 7*q1^2) +
p1*(8*q1 - 6*q1^3))*q2^3 + 2*p1*q1*(-2 + p1 + q1)*(2 + p1 + q1)*
q2^4) + (3 - 6*p2^2 - 6*q2^2 + 6*p1*q1*(p2^2 - 8*p2*q2 + q2^2) +
p1^2*(-2 + 5*p2^2 - 6*p2*q2 + 5*q2^2) +
q1^2*(-2 + 5*p2^2 - 6*p2*q2 + 5*q2^2))*sigm^2)))/
(4*p1*q1*(2*p2*((b2 + b3*p1)^2 - 2*b2*b3*q1 + b3^2*q1^2)*q2 + sigm^2)^3)

$Cov\,(T_1, T_2) = \tau_{12} \rightarrow [\nabla h_1\,(\boldsymbol{\theta})]'\,\Sigma\,[\nabla h_2\,(\boldsymbol{\theta})] =$
(p1*p2*q1*q2*(-2*b1^4*b3*p1^2*q1^2*(p2 - q2)*
(b3*(p1 - q1)*(1 + p2^2*(-2 + p1^2 + 2*p1*q1 + q1^2) -
2*p2*(p1^2 + 4*p1*q1 + q1^2)*q2 + (-2 + p1^2 + 2*p1*q1 + q1^2)*q2^2) -
2*b2*(4*p1*p2*q1*q2 + p1^2*(-1 + p2^2 + 2*p2*q2 + q2^2) +
q1^2*(-1 + p2^2 + 2*p2*q2 + q2^2))) -
b1^3*p1*q1*(2*b2*b3^2*(p1^4*p2*q2*(4*p2^2 + 3*p2*q2 + 4*q2^2) +
p2*q1^2*q2*(4*p2^2*(-1 + q1^2) + 3*p2*q1^2*q2 + 4*(-1 + q1^2)*q2^2) -
2*p1^2*p2*q2*(2*p2^2*(1 + 9*q1^2) - 27*p2*q1^2*q2 +
2*(1 + 9*q1^2)*q2^2) - p1^3*q1*(2*p2^4 + p2^3*q2 + p2*q2^3 +
2*p2^2*(-1 + q2^2) + 2*q2^2*(-1 + q2^2)) -
p1*q1*(2*p2^4*q1^2 + p2^3*(-8 + q1^2)*q2 + p2*(-8 + q1^2)*q2^3 +
2*p2^2*q1^2*(-1 + q2^2) + 2*q1^2*q2^2*(-1 + q2^2))) +
2*b3^3*(p1 - q1)*(p1^4*p2*q2*(2*p2^2 + p2*q2 + 2*q2^2) +
p2*q1^2*q2*(2*p2^2*(-1 + q1^2) + p2*q1^2*q2 + 2*(-1 + q1^2)*q2^2) +
p1^3*q1*(3*p2^4 - 5*p2^3*q2 + 12*p2^2*q2^2 - 5*p2*q2^3 + 3*q2^4) +
p1^2*(6*p2^4*q1^2 + 46*p2^2*q1^2*q2^2 - 2*p2*(1 + 14*q1^2)*q2^3 +
6*q1^2*q2^4 - 2*p2^3*(q2 + 14*q1^2*q2)) +
p1*q1*(p2^4*(-4 + 3*q1^2) + p2^3*(8 - 5*q1^2)*q2 + q2^2 +
p2*(8 - 5*q1^2)*q2^3 + (-4 + 3*q1^2)*q2^4 +
p2^2*(1 + 4*(-2 + 3*q1^2)*q2^2))) +
b2*p2*q2*(2*b2^2*(p1^2*p2*q2 + p2*q1^2*q2 +
p1*q1*(p2^2 - 8*p2*q2 + q2^2)) + (p1^2 + q1^2)*sigm^2) +
b3*(p1 - q1)*(2*b2^2*p2*q2*(p2^2*(-2 + 2*p1^2 + 7*p1*q1 + 2*q1^2) +
p2*(3*p1^2 - 8*p1*q1 + 3*q1^2)*q2 + (-2 + 2*p1^2 + 7*p1*q1 + 2*q1^2)*
q2^2) + (2*p2^2*(-1 + p1^2 + 2*p1*q1 + q1^2) +
p2*(p1^2 + 12*p1*q1 + q1^2)*q2 + 2*(-1 + p1^2 + 2*p1*q1 + q1^2)*
q2^2)*sigm^2)) - b1^2*b3*p1*q1*(p2 - q2)*
(2*b3^3*(p1 - q1)*(p1^4*p2*q2*(2 + 3*p2^2 + p2*q2 + 3*q2^2) +
p1^3*q1*(3*p2^4 + 5*p2^3*q2 + 12*p2^2*q2^2 + 5*p2*q2^3 + 3*q2^4) +
p2*q1^2*q2*(-1 + p2^2*(-4 + 3*q1^2) + p2*q1^2*q2 - 4*q2^2 +
q1^2*(2 + 3*q2^2)) + p1*q1*(3*p2^4*q1^2 + p2^3*(4 + 5*q1^2)*q2 +
3*q2^2*(-1 + q1^2*q2^2) + 3*p2^2*(-1 + 4*q1^2*q2^2) +
p2*q2*(10 + (4 + 5*q1^2)*q2^2)) +
p1^2*(6*p2^4*q1^2 - 2*p2^3*(2 + 11*q1^2)*q2 + 30*p2^2*q1^2*q2^2 +

6*q1^2*q2^4 - p2*(q2 - 4*q1^2*q2 + (4 + 22*q1^2)*q2^3))) +
2*b2*b3^2*(p1^4*p2*q2*(6 + p2^2 + 7*p2*q2 + q2^2) +
p2*q1^2*q2*(-5 + p2^2*(-2 + q1^2) + 7*p2*q1^2*q2 - 2*q2^2 +
q1^2*(6 + q2^2)) + p1^3*q1*(6*p2^4 + 7*p2^3*q2 +
6*q2^2*(-1 + q2^2) + 6*p2^2*(-1 + 3*q2^2) + p2*q2*(12 + 7*q2^2)) -
p1^2*p2*q2*(5 + p2^2*(2 + 66*q1^2) - 14*p2*q1^2*q2 + 2*q2^2 +
6*q1^2*(-2 + 11*q2^2)) + p1*q1*(6*p2^4*q1^2 + p2^3*(8 + 7*q1^2)*q2 +
6*q1^2*q2^2*(-1 + q2^2) + 6*p2^2*q1^2*(-1 + 3*q2^2) +
p2*q2*(8*(1 + q2^2) + q1^2*(12 + 7*q2^2)))) +
b2*p2*q2*(2*b2^2*(p1^2*(-2 + 2*p2^2 + 7*p2*q2 + 2*q2^2) +
q1^2*(-2 + 2*p2^2 + 7*p2*q2 + 2*q2^2) +
p1*q1*(3*p2^2 - 8*p2*q2 + 3*q2^2)) + (3*p1^2 + 4*p1*q1 + 3*q1^2)*
sigm^2) + b3*(p1 - q1)*(2*b2^2*p2*q2*(2*p2^2 + 13*p2*q1^2*q2 +
p1^2*(2 + 13*p2*q2) + 2*(-2 + q1^2 + q2^2) +
p1*q1*(13*p2^2 + 24*p2*q2 + 13*q2^2)) +
(-1 + 2*q1^2 + p2^2*(-4 + 3*q1^2) + p2*q1^2*q2 - 4*q2^2 +
3*q1^2*q2^2 + 10*p1*q1*(p2^2 + 4*p2*q2 + q2^2) +
p1^2*(2 + 3*p2^2 + p2*q2 + 3*q2^2))*sigm^2)) +
b1*(-(b3^5*(p1 - q1)*(8*p1^6*p2^2*q2^2*(p2^2 + q2^2) +
8*p2^2*q1^4*(-1 + q1^2)*q2^2*(p2^2 + q2^2) +
2*p1^4*(p2^6*q1^2 + 6*p2^5*q1^2*q2 - p2^4*(4 + q1^2)*q2^2 +
20*p2^3*q1^2*q2^3 - p2^2*(4 + q1^2)*q2^4 + 6*p2*q1^2*q2^5 +
q1^2*q2^6) - p1^5*p2*q1*q2*(p2^4 - 4*p2^3*q2 + q2^2*(-8 + q2^2) -
4*p2*q2*(-4 + q2^2) + 2*p2^2*(-4 + 5*q2^2)) +
2*p1^2*q1^2*(p2^6*(4 + q1^2) + 2*p2^5*(-4 + 3*q1^2)*q2 -
p2^4*(5 + (-4 + q1^2)*q2^2) - p2^2*q2^2*(34 + (-4 + q1^2)*q2^2) +
q2^4*(-5 + (4 + q1^2)*q2^2) + 2*p2^3*q2*(11 + 2*(-4 + 5*q1^2)*
q2^2) + p2*(22*q2^3 + (-8 + 6*q1^2)*q2^5)) +
p1*p2*q1^3*q2*(-(p2^4*q1^2) + 4*p2^3*(4 + q1^2)*q2 +
p2^2*(-7 + q1^2*(8 - 10*q2^2)) - q2^2*(7 + q1^2*(-8 + q2^2)) +
4*p2*q2*(q1^2*(-4 + q2^2) + 4*(1 + q2^2))) +
p1^3*q1*(4*p2^6*q1^2 - 26*p2^5*q1^2*q2 + 4*p2^4*(4 + 13*q1^2)*q2^2 +
4*q1^2*q2^6 + p2^3*q2*(-7 + q1^2*(16 - 68*q2^2)) +
p2*q2^3*(-7 + q1^2*(16 - 26*q2^2)) + 4*p2^2*q2^2*
(4*(1 + q2^2) + q1^2*(-8 + 13*q2^2))))) +
b2*b3^4*(-20*p1^6*p2^2*q2^2*(p2^2 + q2^2) - 20*p2^2*q1^4*(-1 + q1^2)*
q2^2*(p2^2 + q2^2) + p1^5*p2*q1*q2*(7*p2^4 + 4*p2^3*q2 +
4*p2*q2*(4 + q2^2) + q2^2*(-12 + 7*q2^2) + 2*p2^2*(-6 + 43*q2^2)) -
4*p1^4*(5*p2^6*q1^2 - p2^5*q1^2*q2 - p2*q1^2*q2^3*(-14 + q2^2) +
5*q1^2*q2^4*(-1 + q2^2) + 2*p2^3*q1^2*q2*(7 + 13*q2^2) +
p2^2*(-5*q2^4 + 2*q1^2*q2^2*(-9 + 4*q2^2)) +
p2^4*(-5*q2^2 + q1^2*(-5 + 8*q2^2))) - 4*p1^2*q1^2*
(5*p2^6*q1^2 - p2^5*(4 + q1^2)*q2 + 5*q1^2*q2^4*(-1 + q2^2) +
p2*q2^3*(8 - 4*q2^2 - q1^2*(-14 + q2^2)) + 2*p2^2*q2^2*
(-8 - 9*q2^2 + q1^2*(-9 + 4*q2^2)) +
p2^4*(-18*q2^2 + q1^2*(-5 + 8*q2^2)) + 2*p2^3*q2*
(4 - 4*q2^2 + q1^2*(7 + 13*q2^2))) + p1*p2*q1^3*q2*
(p2^4*(-12 + 7*q1^2) + 4*p2^3*(-14 + q1^2)*q2 +
q2^2*(17 - 12*q2^2 + q1^2*(-12 + 7*q2^2)) +
4*p2*q2*(q1^2*(4 + q2^2) - 2*(4 + 7*q2^2)) +
p2^2*(17 - 24*q2^2 + 2*q1^2*(-6 + 43*q2^2))) +
p1^3*p2*q1*q2*(2*p2^4*(-6 + 43*q1^2) - 8*p2^3*(7 + 13*q1^2)*q2 -
8*p2*q2*(4 + 7*q2^2 + q1^2*(-4 + 13*q2^2)) +
p2^2*(17 - 24*q2^2 + 4*q1^2*(-6 + 79*q2^2)) +
q2^2*(17 - 12*q2^2 + q1^2*(-24 + 86*q2^2)))) -
b2*p1*p2*q1*q2*sigm^2*(b2^2*(p2^2 + q2^2) + 2*sigm^2) +

```
2*b2*b3^2*(b2^2*p2*q2*(2*p1^4*p2*q2*(p2^2 + q2^2) +
2*p2*q1^2*(-1 + q1^2)*q2*(p2^2 + q2^2) -
p1^2*(3*p2^4*q1^2 - 2*p2^3*(-1 + q1^2)*q2 + 54*p2^2*q1^2*q2^2 -
2*p2*(-1 + q1^2)*q2^3 + 3*q1^2*q2^4) +
p1^3*q1*(-4*p2^4 + p2^3*q2 + p2*q2*(-8 + q2^2) -
4*q2^2*(-1 + q2^2) + 4*p2^2*(1 + 9*q2^2)) +
p1*q1^3*(-4*p2^4 + p2^3*q2 + p2*q2*(-8 + q2^2) -
4*q2^2*(-1 + q2^2) + 4*p2^2*(1 + 9*q2^2))) +
(-3*p1^4*p2*q2*(p2^2 + q2^2) - 3*p2*q1^2*(-1 + q1^2)*q2*
(p2^2 + q2^2) + p1^2*p2*q2*(p2^2*(3 + 4*q1^2) + 8*p2*q1^2*q2 +
(3 + 4*q1^2)*q2^2) + p1^3*q1*(-3*p2^4 + p2^3*q2 +
p2*q2*(-8 + q2^2) - 3*q2^2*(-1 + q2^2) + p2^2*(3 + 4*q2^2)) +
p1*(-3*p2^4*q1^3 + p2^3*q1*(-8 + q1^2)*q2 - 3*q1^3*q2^2*
(-1 + q2^2) + p2^2*q1^3*(3 + 4*q2^2) +
p2*(-8*q1*q2^3 + q1^3*q2*(-8 + q2^2))))*sigm^2) -
b3^3*(p1 - q1)*(2*b2^2*p2*q2*(6*p1^4*p2*q2*(p2^2 + q2^2) +
6*p2*q1^2*(-1 + q1^2)*q2*(p2^2 + q2^2) + p1^2*(p2^2 + q2^2)*
(7*p2^2*q1^2 + 6*p2*(-1 + 3*q1^2)*q2 + 7*q1^2*q2^2) +
p1^3*q1*(p2^4 + 7*p2^3*q2 + q2^2*(-2 + q2^2) + p2*q2*(8 + 7*q2^2) -
2*p2^2*(1 + 33*q2^2)) + p1*q1*(p2^4*(6 + q1^2) +
p2^3*(12 + 7*q1^2)*q2 + q2^2*(-5 + 6*q2^2 + q1^2*(-2 + q2^2)) +
p2*q2*(8 + 12*q2^2 + q1^2*(8 + 7*q2^2)) -
p2^2*(5 - 12*q2^2 + q1^2*(2 + 66*q2^2)))) +
(6*p1^4*p2*q2*(p2^2 + q2^2) + 6*p2*q1^2*(-1 + q1^2)*q2*(p2^2 + q2^2) +
p1^2*(6*p2^4*q1^2 + 2*p2^3*(-3 + 20*q1^2)*q2 - 60*p2^2*q1^2*q2^2 +
2*p2*(-3 + 20*q1^2)*q2^3 + 6*q1^2*q2^4) -
p1^3*q1*(p2^4 - 10*p2^3*q2 + q2^2*(-4 + q2^2) + 2*p2^2*(-2 + q2^2) +
p2*(8*q2 - 10*q2^3)) + p1*q1*(-(p2^4*(-2 + q1^2)) +
2*p2^3*(2 + 5*q1^2)*q2 + q2^2*(-5 + 2*q2^2 - q1^2*(-4 + q2^2)) +
p2^2*(-5 + 4*q2^2 - 2*q1^2*(-2 + q2^2)) +
2*p2*q2*(2*(3 + q2^2) + q1^2*(-4 + 5*q2^2))))*sigm^2) +
b3*(p1 - q1)*(4*b2^4*p2^2*q2^2*(p2^2*(-1 + p1^2 + 2*p1*q1 + q1^2) +
4*p1*p2*q1*q2 + (-1 + p1^2 + 2*p1*q1 + q1^2)*q2^2) -
b2^2*p1*p2*q1*q2*(3*p2^2 + 4*p2*q2 + 3*q2^2)*sigm^2 -
(p2^2*(-1 + p1^2 + 2*p1*q1 + q1^2) + 8*p1*p2*q1*q2 +
(-1 + p1^2 + 2*p1*q1 + q1^2)*q2^2)*sigm^4)) -
b3*(p2 - q2)*(b3^5*(p1 - q1)*(4*p1^6*p2^2*q2^2 + 4*p2^2*q1^4*(-1 + q1^2)*
q2^2 + p1^5*p2*q1*q2*(-3*p2^4 + 4*p2^3*q2 + 4*q2^2 - 3*q2^4 +
p2^2*(4 - 6*q2^2) + 4*p2*q2*(-2 + q2^2)) +
4*p1^4*p2*q2*(p2^4*q1^2 - 2*p2^3*q1^2*q2 + 2*p2^2*q1^2*q2^2 +
q1^2*q2^4 - p2*(q2 - 3*q1^2*q2 + 2*q1^2*q2^3)) +
4*p1^2*q1^2*(p2^6 + p2^5*(-2 + q1^2)*q2 + q2^4*(-1 + q2^2) +
p2^4*(-1 + (3 - 2*q1^2)*q2^2) + 2*p2^3*q2*(2 + (-2 + q1^2)*q2^2) +
p2*q2^3*(4 + (-2 + q1^2)*q2^2) + p2^2*q2^2*(-8 + 3*q2^2 +
q1^2*(3 - 2*q2^2))) + p1^3*p2*q1*q2*(p2^4*(4 - 6*q1^2) +
8*p2^3*q1^2*q2 + p2^2*(-5 + 8*q2^2 + q1^2*(8 - 12*q2^2)) +
q2^2*(-5 + 4*q2^2 + q1^2*(8 - 6*q2^2)) +
8*p2*q2*(2 + q1^2*(-2 + q2^2))) + p1*p2*q1^3*q2*
(p2^4*(4 - 3*q1^2) + 4*p2^3*q1^2*q2 +
p2^2*(-5 + 8*q2^2 + q1^2*(4 - 6*q2^2)) +
q2^2*(-5 + 4*q2^2 + q1^2*(4 - 3*q2^2)) +
4*p2*q2*(4 + q1^2*(-2 + q2^2)))) +
b2*b3^4*(2*p1^6*p2^2*q2^2*(4 + p2^2 + 2*p2*q2 + q2^2) +
2*p2^2*q1^4*q2^2*(-5 + q1^2*(4 + p2^2 + 2*p2*q2 + q2^2)) -
p1^5*p2*q1*q2*(p2^4 - 12*p2^3*q2 + 26*p2^2*q2^2 + q2^4 -
4*p2*q2*(-4 + 3*q2^2)) + 2*p1^4*(4*p2^6*q1^2 + 2*p2^5*q1^2*q2 +
```

4*q1^2*q2^4*(-1 + q2^2) - p2^4*q1^2*(4 + q2^2) +
2*p2*q1^2*q2^3*(4 + q2^2) + 2*p2^3*q1^2*q2*(4 + 13*q2^2) -
p2^2*q2^2*(5 + q1^2*(-4 + q2^2))) - p1*p2*q1^3*q2*
(p2^4*(-8 + q1^2) - 12*p2^3*q1^2*q2 + q2^2*(7 + (-8 + q1^2)*q2^2) +
p2^2*(7 + 2*(-8 + 13*q1^2)*q2^2) - 4*p2*q2*
(11 + q1^2*(-4 + 3*q2^2))) + p1^3*p2*q1*q2*(p2^4*(8 - 10*q1^2) +
40*p2^3*q1^2*q2 - 7*q2^2 + (8 - 10*q1^2)*q2^4 +
p2^2*(-7 + (16 - 68*q1^2)*q2^2) + 4*p2*q2*
(11 + 2*q1^2*(-4 + 5*q2^2))) + 2*p1^2*q1^2*(4*p2^6*q1^2 +
2*p2^5*(-4 + q1^2)*q2 + 4*q1^2*q2^4*(-1 + q2^2) -
p2^4*q1^2*(4 + q2^2) - p2^2*q2^2*(34 + q1^2*(-4 + q2^2)) +
2*p2*q2^3*(4 - 4*q2^2 + q1^2*(4 + q2^2)) +
2*p2^3*q2*(4 - 8*q2^2 + q1^2*(4 + 13*q2^2)))) +
b2*sigm^2*(b2^2*p2*q2*(2*p1^2*(-1 + p2^2 + 2*p2*q2 + q2^2) +
2*q1^2*(-1 + p2^2 + 2*p2*q2 + q2^2) + p1*q1*(p2^2 + 12*p2*q2 +
q2^2)) + (8*p1*p2*q1*q2 + p1^2*(-1 + p2^2 + 2*p2*q2 + q2^2) +
q1^2*(-1 + p2^2 + 2*p2*q2 + q2^2))*sigm^2) +
b2*b3^2*(2*b2^2*p2*q2*(p1^4*p2*q2*(-4 + 3*p2^2 + 6*p2*q2 + 3*q2^2) +
p2*q1^2*q2*(1 + q1^2*(-4 + 3*p2^2 + 6*p2*q2 + 3*q2^2)) +
p1^3*q1*(2*p2^4 - 5*p2^3*q2 + 2*q2^2*(-1 + q2^2) -
2*p2^2*(1 + 14*q2^2) + p2*(8*q2 - 5*q2^3)) +
p1*q1^3*(2*p2^4 - 5*p2^3*q2 + 2*q2^2*(-1 + q2^2) -
2*p2^2*(1 + 14*q2^2) + p2*(8*q2 - 5*q2^3)) +
p1^2*(p2^4*q1^2 + 12*p2^3*q1^2*q2 + 46*p2^2*q1^2*q2^2 + q1^2*q2^4 +
p2*(q2 - 8*q1^2*q2 + 12*q1^2*q2^3))) +
(-(p1^4*p2*q2*(-2 + p2^2 - 6*p2*q2 + q2^2)) +
p1^2*p2*q2*(-5 - 2*p2^2*(-2 + q1^2) - 60*p2*q1^2*q2 + 4*q2^2 -
2*q1^2*(-2 + q2^2)) + p2*q1^2*q2*(-5 - p2^2*(-4 + q1^2) +
6*p2*q1^2*q2 + 4*q2^2 - q1^2*(-2 + q2^2)) +
2*p1^3*q1*(3*p2^4 + 5*p2^3*q2 + 3*q2^2*(-1 + q2^2) +
p2*q2*(2 + 5*q2^2) + p2^2*(-3 + 20*q2^2)) +
2*p1*q1*(3*p2^4*q1^2 + p2^3*(-4 + 5*q1^2)*q2 +
3*q1^2*q2^2*(-1 + q2^2) + p2^2*q1^2*(-3 + 20*q2^2) +
p2*q2*(6 - 4*q2^2 + q1^2*(2 + 5*q2^2))))*sigm^2) +
2*b3^3*(p1 - q1)*(b2^2*p2*q2*(3*p1^4*p2*q2*(p2 + q2)^2 +
3*p2*q1^2*q2*(-1 + p2^2*q1^2 + 2*p2*q1^2*q2 + q1^2*q2^2) +
p1^3*q1*(3*p2^4 + 5*p2^3*q2 + q2^2*(-4 + 3*q2^2) +
p2*q2*(4 + 5*q2^2) - 2*p2^2*(2 + 11*q2^2)) +
p1^2*(p2^4*q1^2 + 12*p2^3*q1^2*q2 + 30*p2^2*q1^2*q2^2 + q1^2*q2^4 +
3*p2*q2*(-1 + 4*q1^2*q2^2)) + p1*q1*(p2^4*(2 + 3*q1^2) +
5*p2^3*q1^2*q2 + q2^2*(-1 + 2*q2^2 + q1^2*(-4 + 3*q2^2)) +
p2*q2*(10 + q1^2*(4 + 5*q2^2)) - p2^2*(1 - 4*q2^2 +
q1^2*(4 + 22*q2^2)))) + (-(p1^4*p2*q2*(-2 + p2^2 + q2^2)) -
p2*q1^2*(-1 + q1^2)*q2*(-2 + p2^2 + q2^2) +
p1^3*q1*(-p2^4 + 3*p2^3*q2 + q2^2 - q2^4 + p2*q2*(-2 + 3*q2^2) +
p2^2*(1 + 6*q2^2)) + p1^2*p2*q2*(-2 + p2^2*(1 + 6*q1^2) -
16*p2*q1^2*q2 + q2^2 + q1^2*(4 + 6*q2^2)) +
p1*q1*(-(p2^4*(-2 + q1^2)) + p2^3*(-2 + 3*q1^2)*q2 -
(-2 + q1^2)*q2^2*(-1 + q2^2) + p2*q2*(6 - 2*q2^2 +
q1^2*(-2 + 3*q2^2)) + p2^2*(-2 + 4*q2^2 + q1^2*(1 + 6*q2^2))))*
sigm^2) + b3*(p1 - q1)*(2*b2^4*p2^2*q2^2*
(1 + p1^2*(-2 + p2^2 + 2*p2*q2 + q2^2) +
q1^2*(-2 + p2^2 + 2*p2*q2 + q2^2) - 2*p1*q1*(p2^2 + 4*p2*q2 +
q2^2)) + b2^2*p2*q2*(-1 - 4*q1^2 + p2^2*(2 + 3*q1^2) +
10*p2*q1^2*q2 + 2*q2^2 + 3*q1^2*q2^2 +
p1*q1*(p2^2 + 40*p2*q2 + q2^2) + p1^2*(-4 + 3*p2^2 + 10*p2*q2 +

3*q2^2))*sigm^2 - (-14*p1*p2*q1*q2 + p1^2*(-1 + p2^2 + q2^2) +
(-1 + q1^2)*(-1 + p2^2 + q2^2))*sigm^4))))/
(2*(p1*q1*(2*b2^2*p2*q2 + 4*b2*b3*p2*(p1 - q1)*q2 +
2*b3^2*p2*(p1^2 + q1^2)*q2 + sigm^2))^(3/2)*
(p2*q2*(2*b1^2*p1*q1 + 4*b1*b3*p1*q1*(p2 - q2) +
2*b3^2*p1*q1*(p2^2 + q2^2) + sigm^2))^(3/2))

When $b_3 = 0$, $Cov\,(T_1, T_2)$ is still nonzero:
$Cov\,(T_1, T_2) =$
-(b1*b2*p1^2*p2^2*q1^2*q2^2*(b2^2*(p2^2 + q2^2)*sigm^2 + 2*sigm^4 +
b1^2*(2*b2^2*(p1*p2^2*q1 + p2*(p1^2 - 8*p1*q1 + q1^2)*q2 + p1*q1*q2^2) +
(p1^2 + q1^2)*sigm^2)))/(2*(p2*q2*(2*b1^2*p1*q1 + sigm^2))^(3/2)*
(p1*q1*(2*b2^2*p2*q2 + sigm^2))^(3/2))

$E\,(T_{12}) \to \sqrt{n-4}h_{12}\,(\boldsymbol{\theta}) =$
sqrt(2/3)*sqrt(n)*sqrt((b1^2*p1*q1 + 2*b1*b3*p1*q1*(p2 - q2) + b2^2*p2*q2 +
2*b2*b3*p2*(p1 - q1)*q2 + b3^2*(p1*q1*(p2 - q2)^2 + p1^2*p2*q2 +
p2*q1^2*q2))/sigm^2)

$Var\,(T_{12}) \to [\nabla h_{12}\,(\boldsymbol{\theta})]'\,\Sigma\,[\nabla h_{12}\,(\boldsymbol{\theta})] =$
(2*b1^4*p1*q1*(p1^2 + 4*p1*q1 + q1^2) + 8*b1^3*b3*p1*q1*
(p1^2 + 4*p1*q1 + q1^2)*(p2 - q2) + 2*b2^4*p2*q2*(p2^2 + 4*p2*q2 + q2^2) +
8*b2^3*b3*p2*(p1 - q1)*q2*(p2^2 + 4*p2*q2 + q2^2) +
4*b1^2*(8*b2^2*p1*p2*q1*q2 - 2*b2*b3*(p1 - q1)*
(p2^2*(-1 + p1^2 + 2*p1*q1 + q1^2) - 2*p1*p2*q1*q2 +
(-1 + p1^2 + 2*p1*q1 + q1^2)*q2^2) +
b3^2*(p1^4*(p2^2 + q2^2) + q1^2*(-1 + q1^2)*(p2^2 + q2^2) +
p1^2*(-1 + 6*q1^2)*(p2^2 + q2^2) + p1^3*q1*(p2^2 - 4*p2*q2 + q2^2) +
p1*q1*(p2^2*(4 + q1^2) - 4*p2*q1^2*q2 + (4 + q1^2)*q2^2)) +
2*p1*q1*sigm^2) + 8*b2*b3*(p1 - q1)*
(b3^2*(-(p2^2*q1^2) + p2^4*q1^2 - 4*p1*p2*q1*(p2 - q2)^2*q2 +
p2*(q2 + 2*q1^2*q2) + q1^2*q2^2*(-1 + q2^2) +
p1^2*(-p2^2 + p2^4 + 2*p2*q2 - q2^2 + q2^4)) + 2*p2*q2*sigm^2) +
4*b2^2*(b3^2*(-4*p1*p2*q1*q2*(p2^2 + q2^2) +
p1^2*(p2^4 + p2^3*q2 + q2^2*(-1 + q2^2) + p2*q2*(4 + q2^2) +
p2^2*(-1 + 6*q2^2)) + q1^2*(p2^4 + p2^3*q2 + q2^2*(-1 + q2^2) +
p2*q2*(4 + q2^2) + p2^2*(-1 + 6*q2^2))) + 2*p2*q2*sigm^2) +
b3^2*(b3^2*(-16*p1^3*p2*q1*(p2 - q2)^2*q2 - 8*p1*q1*(p2 - q2)^2*
(-1 + 2*p2*q1^2*q2) + p1^4*(3*p2^4 - 2*p2^2*q2^2 + 3*q2^4) +
q1^2*(3*p2^4*q1^2 + 8*p2*q2 + 3*q2^2*(-1 + q1^2*q2^2) -
p2^2*(3 + 2*q1^2*q2^2)) - p1^2*(2*p2^4*q1^2 - 32*p2^3*q1^2*q2 +
q2^2*(3 + 2*q1^2*q2^2) + p2^2*(3 + 52*q1^2*q2^2) -
8*p2*(q2 + 4*q1^2*q2^3))) + 8*(p1*q1*(p2 - q2)^2 + p1^2*p2*q2 +
p2*q1^2*q2)*sigm^2) - 4*b1*b3*(p2 - q2)*
(2*b2^2*(-2*p1*p2*q1*q2 + p1^2*(-1 + p2^2 + 2*p2*q2 + q2^2) +
q1^2*(-1 + p2^2 + 2*p2*q2 + q2^2)) - b2*b3*(p1 - q1)*
(3 - 2*q1^2 + p2^2*(-2 + q1^2) - 2*p2*q1^2*q2 - 2*q2^2 + q1^2*q2^2 +
p1^2*(-2 + p2^2 - 2*p2*q2 + q2^2) - 2*p1*q1*(p2^2 + 8*p2*q2 + q2^2)) -
2*(b3^2*(-4*p1^3*p2*q1*q2 + p1^4*(p2^2 + q2^2) + q1^2*(-1 + q1^2)*
(p2^2 + q2^2) - p1^2*(p2^2 - 8*p2*q1^2*q2 + q2^2) +
p1*(q1 + 2*p2^2*q1 - 4*p2*q1^3*q2 + 2*q1*q2^2)) + 2*p1*q1*sigm^2))))/
(24*(b1^2*p1*q1 + 2*b1*b3*p1*q1*(p2 - q2) + b2^2*p2*q2 +
2*b2*b3*p2*(p1 - q1)*q2 + b3^2*(p1*q1*(p2 - q2)^2 + p1^2*p2*q2 +
p2*q1^2*q2))*sigm^2)

$Cov\,(T_{12}, T_1) = \tau_{12,1} \to [\nabla h_{12}\,(\boldsymbol{\theta})]'\,\Sigma\,[\nabla h_1\,(\boldsymbol{\theta})] =$

```
(p1*q1*(b1^2*b3*p1*q1*(p2 - q2)*
(2*b2^2*(12*p1*p2*q1*q2 + p1^2*(-2 + 2*p2^2 + 7*p2*q2 + 2*q2^2) +
q1^2*(-2 + 2*p2^2 + 7*p2*q2 + 2*q2^2)) - 2*b2*b3*(p1 - q1)*
(3 - 2*q1^2 + p2^2*(-6 + 5*q1^2) - 8*p2*q1^2*q2 - 6*q2^2 +
5*q1^2*q2^2 + 6*p1*q1*(p2^2 - 6*p2*q2 + q2^2) +
p1^2*(-2 + 5*p2^2 - 8*p2*q2 + 5*q2^2)) +
b3^2*(6*p1^4*p2*q2 + 6*p2*q1^4*q2 - 4*p1^3*q1*(p2^2 - 7*p2*q2 + q2^2) -
4*p1^2*q1^2*(2*p2^2 + p2*q2 + 2*q2^2) -
4*p1*q1*(1 + p2^2*(-2 + q1^2) - 7*p2*q1^2*q2 + (-2 + q1^2)*q2^2)) +
3*(p1^2 + 4*p1*q1 + q1^2)*sigm^2) +
b1^3*p1*q1*(4*b2*b3*p2*(p1 - q1)*(p1 + q1)^2*q2 +
2*b2^2*p2*(p1^2 + q1^2)*q2 + (p1^2 + 4*p1*q1 + q1^2)*
(2*b3^2*p2*(p1 - q1)^2*q2 + sigm^2)) -
b1*(2*b2^4*p1*p2*q1*q2*(p2^2 - 8*p2*q2 + q2^2) +
8*b2^3*b3*p2*(p1 - q1)*q2*(p2^2*(-1 + p1^2 + 3*p1*q1 + q1^2) -
3*p1*p2*q1*q2 + (-1 + p1^2 + 3*p1*q1 + q1^2)*q2^2) -
b3^4*(4*p1^6*p2*q2*(p2^2 + q2^2) + 4*p2*q1^4*(-1 + q1^2)*q2*
(p2^2 + q2^2) + p1^5*q1*(p2^4 - 10*p2^3*q2 + 2*p2^2*q2^2 -
10*p2*q2^3 + q2^4) + p1^4*(-6*p2^4*q1^2 + 4*p2^3*(-1 + 6*q1^2)*q2 +
4*p2^2*q1^2*q2^2 + 4*p2*(-1 + 6*q1^2)*q2^3 - 6*q1^2*q2^4) -
2*p1^2*q1^2*(p2^4*(-8 + 3*q1^2) - 4*p2^3*(-5 + 3*q1^2)*q2 -
2*p2^2*(-2 + (8 + q1^2)*q2^2) + q2^2*(4 + (-8 + 3*q1^2)*q2^2) -
4*p2*q2*(2 + (-5 + 3*q1^2)*q2^2)) +
p1*q1^3*(p2^4*(-4 + q1^2) + 2*p2^3*(12 - 5*q1^2)*q2 +
q2^2*(3 + (-4 + q1^2)*q2^2) + p2^2*(3 + 2*(-4 + q1^2)*q2^2) -
2*p2*q2*(4 + (-12 + 5*q1^2)*q2^2)) +
p1^3*q1*(-2*p2^4*(2 + 7*q1^2) + 4*p2^3*(6 + 7*q1^2)*q2 + 3*q2^2 -
2*(2 + 7*q1^2)*q2^4 + 4*p2*q2*(-2 + (6 + 7*q1^2)*q2^2) +
p2^2*(3 - 4*(2 + 15*q1^2)*q2^2))) +
b3^2*(-2*p1^4*(p2^2 + q2^2) - 2*q1^2*(-1 + q1^2)*(p2^2 + q2^2) +
p1^3*q1*(p2^2 + 4*p2*q2 + q2^2) + 2*p1^2*(p2^2 - 8*p2*q1^2*q2 +
q2^2) + p1*q1*(p2^2*(-8 + q1^2) + 4*p2*q1^2*q2 + (-8 + q1^2)*q2^2))*
sigm^2 - 4*p1*q1*sigm^4 +
2*b2^2*(b3^2*(6*p1^4*p2*q2*(p2^2 + q2^2) + 6*p2*q1^2*(-1 + q1^2)*q2*
(p2^2 + q2^2) - 2*p1^2*p2*q2*(p2^2*(3 + 14*q1^2) - 4*p2*q1^2*q2 +
(3 + 14*q1^2)*q2^2) + p1^3*q1*(2*p2^4 + 3*p2^3*q2 +
2*q2^2*(-1 + q2^2) - 2*p2^2*(1 + 2*q2^2) + p2*q2*(8 + 3*q2^2)) +
p1*q1*(2*p2^4*q1^2 + p2^3*(8 + 3*q1^2)*q2 + 2*q1^2*q2^2*
(-1 + q2^2) - 2*p2^2*q1^2*(1 + 2*q2^2) +
p2*(8*q2^3 + q1^2*q2*(8 + 3*q2^2)))) - 6*p1*p2*q1*q2*sigm^2) +
4*b2*b3*(p1 - q1)*(b3^2*p1*q1*(p2^4*(-2 + 4*q1^2) +
p2^3*(-4 + q1^2)*q2 - 2*q1^2*q2^2 - 2*q2^4 + 4*q1^2*q2^4 +
2*p1*q1*(2*p2^4 - 5*p2^3*q2 + 2*p2^2*q2^2 - 5*p2*q2^3 + 2*q2^4) +
p2*q2*(2 - 4*q2^2 + q1^2*(4 + q2^2)) +
p1^2*(4*p2^4 + p2^3*q2 - 2*q2^2 + 4*q2^4 + p2*q2*(4 + q2^2) +
p2^2*(-2 + 6*q2^2)) + p2^2*(-4*q2^2 + q1^2*(-2 + 6*q2^2))) +
(p2^2*(-1 + p1^2 + 2*p1*q1 + q1^2) - p1*p2*q1*q2 +
(-1 + p1^2 + 2*p1*q1 + q1^2)*q2^2)*sigm^2)) -
b3*(p2 - q2)*(2*b2^4*p2*q2*(2*p1^2*(-1 + p2^2 + 2*p2*q2 + q2^2) +
2*q1^2*(-1 + p2^2 + 2*p2*q2 + q2^2) + p1*q1*(p2^2 + 4*p2*q2 + q2^2)) +
2*b2^3*b3*p2*(p1 - q1)*q2*(-3 - 2*q1^2 + p2^2*(2 + 3*q1^2) +
10*p2*q1^2*q2 + 2*q2^2 + 3*q1^2*q2^2 +
6*p1*q1*(p2^2 + 6*p2*q2 + q2^2) + p1^2*(-2 + 3*p2^2 + 10*p2*q2 +
3*q2^2)) + b3^4*(-4*p1^6*p2*q2*(p2^2 + q2^2) -
4*p2*q1^4*(-1 + q1^2)*q2*(p2^2 + q2^2) -
p1^5*q1*(p2^4 - 10*p2^3*q2 - 6*p2^2*q2^2 - 10*p2*q2^3 + q2^4) +
```

```
2*p1^4*(p2^4*q1^2 + 2*p2^3*q2 - 14*p2^2*q1^2*q2^2 + 2*p2*q2^3 +
q1^2*q2^4) + p1*q1^3*(-(p2^4*(-4 + q1^2)) + 2*p2^3*(-8 + 5*q1^2)*
q2 - q2^2*(3 + (-4 + q1^2)*q2^2) +
2*p2*q2*(2 + (-8 + 5*q1^2)*q2^2) + p2^2*(-3 + (8 + 6*q1^2)*q2^2)) +
2*p1^2*q1^2*(p2^4*(-4 + q1^2) + 12*p2^3*q2 + 4*p2*q2*(-1 + 3*q2^2) +
q2^2*(2 + (-4 + q1^2)*q2^2) - 2*p2^2*(-1 + (4 + 7*q1^2)*q2^2)) +
p1^3*q1*(p2^4*(4 + 6*q1^2) - 4*p2^3*(4 + 3*q1^2)*q2 -
4*p2*q2*(-1 + (4 + 3*q1^2)*q2^2) + q2^2*(-3 + (4 + 6*q1^2)*q2^2) +
p2^2*(-3 + (8 + 60*q1^2)*q2^2))) +
b3^2*(-2*p1^4*(p2^2 + q2^2) - 2*q1^2*(-1 + q1^2)*(p2^2 + q2^2) +
p1^3*q1*(p2^2 + 8*p2*q2 + q2^2) + p1*q1*(-2 + p2^2*(-4 + q1^2) +
8*p2*q1^2*q2 + (-4 + q1^2)*q2^2) + 2*p1^2*(p2^2*(1 + 2*q1^2) -
8*p2*q1^2*q2 + (1 + 2*q1^2)*q2^2))*sigm^2 - 4*p1*q1*sigm^4 +
2*b2^2*(b3^2*(-2*p1^4*p2*q2*(-1 + p2^2 - 4*p2*q2 + q2^2) +
2*p2*q1^2*q2*(-(p2^2*(-3 + q1^2)) + 4*p2*q1^2*q2 -
(-3 + q1^2)*(-1 + q2^2)) - 2*p1^2*p2*q2*(3 + p2^2*(-3 + 6*q1^2) +
40*p2*q1^2*q2 - 3*q2^2 + q1^2*(-2 + 6*q2^2)) +
p1^3*q1*(4*p2^4 + 11*p2^3*q2 + 4*q2^2*(-1 + q2^2) +
p2*q2*(4 + 11*q2^2) + p2^2*(-4 + 48*q2^2)) +
p1*q1*(4*p2^4*q1^2 + p2^3*(-12 + 11*q1^2)*q2 +
4*q1^2*q2^2*(-1 + q2^2) + 4*p2^2*q1^2*(-1 + 12*q2^2) +
p2*q2*(10 - 12*q2^2 + q1^2*(4 + 11*q2^2)))) +
(p1^2 + q1^2)*(-1 + p2^2 + 2*p2*q2 + q2^2)*sigm^2) -
b2*b3*(p1 - q1)*(2*b3^2*(p1^4*p2*q2*(-2 + 5*p2^2 - 2*p2*q2 + 5*q2^2) +
p2*q1^2*q2*(3 + p2^2*(-6 + 5*q1^2) - 2*p2*q1^2*q2 - 6*q2^2 +
q1^2*(-2 + 5*q2^2)) - p1^3*q1*(3*p2^4 + 8*p2^3*q2 +
q2^2*(-2 + 3*q2^2) + p2^2*(-2 + 30*q2^2) + 4*p2*(q2 + 2*q2^3)) -
p1^2*(2*p2^4*q1^2 + 6*p2^3*(1 + q1^2)*q2 - 32*p2^2*q1^2*q2^2 +
2*q1^2*q2^4 + p2*q2*(-3 + 6*q2^2 + q1^2*(4 + 6*q2^2))) -
p1*q1*(p2^4*(2 + 3*q1^2) + 4*p2^3*(-3 + 2*q1^2)*q2 +
q2^2*(-3 + 2*q2^2 + q1^2*(-2 + 3*q2^2)) +
2*p2*q2*(3 - 6*q2^2 + q1^2*(2 + 4*q2^2)) +
p2^2*(-3 + 4*q2^2 + q1^2*(-2 + 30*q2^2)))) +
(3 - 2*q1^2 + p2^2*(-2 + q1^2) - 2*p2*q1^2*q2 - 2*q2^2 + q1^2*q2^2 +
p1^2*(-2 + p2^2 - 2*p2*q2 + q2^2) - 2*p1*q1*(p2^2 + 10*p2*q2 +
q2^2))*sigm^2))))/
(4*sqrt(3)*sqrt((b1^2*p1*q1 + 2*b1*b3*p1*q1*(p2 - q2) + b2^2*p2*q2 +
2*b2*b3*p2*(p1 - q1)*q2 + b3^2*(p1*q1*(p2 - q2)^2 + p1^2*p2*q2 +
p2*q1^2*q2))/sigm^2)*sigm^2*
(p1*q1*(2*b2^2*p2*q2 + 4*b2*b3*p2*(p1 - q1)*q2 + 2*b3^2*p2*(p1^2 + q1^2)*
q2 + sigm^2))^(3/2))
```

4.2 Mean and variance of $F_{3|1}$

For F statistic $F_{3|1}$, we have $F_{3|1} \overset{(d)}{\cong} c\chi^2_d$, where $c = \frac{v}{2e}$, $d = \frac{2e^2}{v}$ with

$E\left(F_{3|1}\right) \to \frac{1}{2} tr\left(D^2\left(h_{3|1}\left(\boldsymbol{\theta}_{13}\right)\right)\boldsymbol{\Sigma}\right) \equiv e =$

```
(0.125*(8*b2^2*p2*q2 +
(b3*(b2*(p1 - q1)*(p2^2*(-1 + p1^2 + 2*p1*q1 + q1^2)*(p3 - q3)^2 +
(-1 + p1^2 + 2*p1*q1 + q1^2)*q2^2*(p3 - q3)^2 -
2*p2*q2*(p3^2*(-1 + p1^2 + 2*p1*q1 + q1^2) - 4*p1*p3*q1*q3 +
(-1 + p1^2 + 2*p1*q1 + q1^2)*q3^2)) +
2*b3*p1*q1*(p2^2*(-1 + p1^2 + 2*p1*q1 + q1^2)*(p3 - q3)^2 +
(-1 + p1^2 + 2*p1*q1 + q1^2)*q2^2*(p3 - q3)^2 -
2*p2*q2*(p3^2*(-1 + p1^2 + 2*p1*q1 + q1^2) - 2*p3*(p1 + q1)^2*q3 +
```

(-1 + p1^2 + 2*p1*q1 + q1^2)*q3^2))))/(p1*p3*q1*q3) +
(8*b2^2*p1*p2*p3*q1*q2*q3 + b2*b3*(p1 - q1)*
(p2^2*(-1 + p1^2 + 2*p1*q1 + q1^2)*(p3 - q3)^2 +
(-1 + p1^2 + 2*p1*q1 + q1^2)*q2^2*(p3 - q3)^2 -
2*p2*q2*(p3^2*(-1 + p1^2 + 2*p1*q1 + q1^2) - 4*p1*p3*q1*q3 +
(-1 + p1^2 + 2*p1*q1 + q1^2)*q3^2)) +
b3^2*(-(p1^4*(p2^2*(p3 - q3)^2 + q2^2*(p3 - q3)^2 -
2*p2*q2*(p3^2 + q3^2))) - 2*p1*q1*(p2^2*(p3 - q3)^2 +
q2^2*(p3 - q3)^2 - 2*p2*q2*(p3^2 + q3^2)) -
q1^2*(-1 + q1^2)*(p2^2*(p3 - q3)^2 + q2^2*(p3 - q3)^2 -
2*p2*q2*(p3^2 + q3^2)) + p1^2*(p2^2*(1 + 2*q1^2)*(p3 - q3)^2 +
(1 + 2*q1^2)*q2^2*(p3 - q3)^2 - 2*p2*q2*(p3^2*(1 + 2*q1^2) -
8*p3*q1^2*q3 + (1 + 2*q1^2)*q3^2))))/(p1*p3*q1*q3) + 8*sigm^2))/
(2*b2^2*p2*q2 + 4*b2*b3*p2*(p1 - q1)*q2 + 2*b3^2*p2*(p1^2 + q1^2)*q2 +
sigm^2)

$Var\left(F_{3|1}\right) \rightarrow \frac{1}{2} tr\left(\left[D^2\left(h_{3|1}\left(\boldsymbol{\theta}_{13}\right)\right)\boldsymbol{\Sigma}\right]^2\right) \equiv v =$
(0.03125*(64*b2^4*p2^2*q2^2 +
(b3^2*(b2*(p1 - q1)*(p2^2*(-1 + p1^2 + 2*p1*q1 + q1^2)*(p3 - q3)^2 +
(-1 + p1^2 + 2*p1*q1 + q1^2)*q2^2*(p3 - q3)^2 -
2*p2*q2*(p3^2*(-1 + p1^2 + 2*p1*q1 + q1^2) - 4*p1*p3*q1*q3 +
(-1 + p1^2 + 2*p1*q1 + q1^2)*q3^2)) +
2*b3*p1*q1*(p2^2*(-1 + p1^2 + 2*p1*q1 + q1^2)*(p3 - q3)^2 +
(-1 + p1^2 + 2*p1*q1 + q1^2)*q2^2*(p3 - q3)^2 -
2*p2*q2*(p3^2*(-1 + p1^2 + 2*p1*q1 + q1^2) - 2*p3*(p1 + q1)^2*q3 +
(-1 + p1^2 + 2*p1*q1 + q1^2)*q3^2)))^2)/(p1^2*p3^2*q1^2*q3^2) +
(48*b2^2*b3^2*p2*q2*(-(p1^4*(p2^2*(p3 - q3)^2 + q2^2*(p3 - q3)^2 -
2*p2*q2*(p3^2 + q3^2))) - 2*p1*q1*(p2^2*(p3 - q3)^2 +
q2^2*(p3 - q3)^2 - 2*p2*q2*(p3^2 + q3^2)) -
q1^2*(-1 + q1^2)*(p2^2*(p3 - q3)^2 + q2^2*(p3 - q3)^2 -
2*p2*q2*(p3^2 + q3^2)) + p1^2*(p2^2*(1 + 2*q1^2)*(p3 - q3)^2 +
(1 + 2*q1^2)*q2^2*(p3 - q3)^2 - 2*p2*q2*(p3^2*(1 + 2*q1^2) -
8*p3*q1^2*q3 + (1 + 2*q1^2)*q3^2))))/(p1*p3*q1*q3) +
(8*b2^2*p1*p2*p3*q1*q2*q3 + b2*b3*(p1 - q1)*
(p2^2*(-1 + p1^2 + 2*p1*q1 + q1^2)*(p3 - q3)^2 +
(-1 + p1^2 + 2*p1*q1 + q1^2)*q2^2*(p3 - q3)^2 -
2*p2*q2*(p3^2*(-1 + p1^2 + 2*p1*q1 + q1^2) - 4*p1*p3*q1*q3 +
(-1 + p1^2 + 2*p1*q1 + q1^2)*q3^2)) +
b3^2*(-(p1^4*(p2^2*(p3 - q3)^2 + q2^2*(p3 - q3)^2 -
2*p2*q2*(p3^2 + q3^2))) - 2*p1*q1*(p2^2*(p3 - q3)^2 +
q2^2*(p3 - q3)^2 - 2*p2*q2*(p3^2 + q3^2)) - q1^2*(-1 + q1^2)*
(p2^2*(p3 - q3)^2 + q2^2*(p3 - q3)^2 - 2*p2*q2*(p3^2 + q3^2)) +
p1^2*(p2^2*(1 + 2*q1^2)*(p3 - q3)^2 + (1 + 2*q1^2)*q2^2*(p3 - q3)^2 -
2*p2*q2*(p3^2*(1 + 2*q1^2) - 8*p3*q1^2*q3 + (1 + 2*q1^2)*q3^2))))^2/
(p1^2*p3^2*q1^2*q3^2) + (2*b2*b3*(8*b2*p1*p2*p3*q1*q2*q3 +
b3*(p1 - q1)*(p2^2*(-1 + p1^2 + 2*p1*q1 + q1^2)*(p3 - q3)^2 +
(-1 + p1^2 + 2*p1*q1 + q1^2)*q2^2*(p3 - q3)^2 -
2*p2*q2*(p3^2*(-1 + p1^2 + 2*p1*q1 + q1^2) - 4*p1*p3*q1*q3 +
(-1 + p1^2 + 2*p1*q1 + q1^2)*q3^2)))*
(b2*(p1 - q1)*(p2^2*(-1 + p1^2 + 2*p1*q1 + q1^2)*(p3 - q3)^2 +
(-1 + p1^2 + 2*p1*q1 + q1^2)*q2^2*(p3 - q3)^2 -
2*p2*q2*(p3^2*(-1 + p1^2 + 2*p1*q1 + q1^2) - 4*p1*p3*q1*q3 +
(-1 + p1^2 + 2*p1*q1 + q1^2)*q3^2)) +
b3*(2*p1^3*q1*(p2 - q2)^2*(p3 - q3)^2 -
p1^4*(p2^2*(p3 - q3)^2 + q2^2*(p3 - q3)^2 - 2*p2*q2*(p3^2 + q3^2)) -
q1^2*(-1 + q1^2)*(p2^2*(p3 - q3)^2 + q2^2*(p3 - q3)^2 -

2*p2*q2*(p3^2 + q3^2)) + 2*p1*q1*(p2^2*(-2 + q1^2)*(p3 - q3)^2 +
(-2 + q1^2)*q2^2*(p3 - q3)^2 - 2*p2*q2*(p3^2*(-2 + q1^2) -
2*p3*q1^2*q3 + (-2 + q1^2)*q3^2)) +
p1^2*(p2^2*(1 + 6*q1^2)*(p3 - q3)^2 + (1 + 6*q1^2)*q2^2*(p3 - q3)^2 -
2*p2*q2*(p3^2*(1 + 6*q1^2) - 16*p3*q1^2*q3 + (1 + 6*q1^2)*q3^2)))))/
(p1^2*p3^2*q1^2*q3^2) + 64*b2^2*p2*q2*sigm^2 +
(8*b2*(8*b2*p1*p2*p3*q1*q2*q3 + b3*(p1 - q1)*
(p2^2*(-1 + p1^2 + 2*p1*q1 + q1^2)*(p3 - q3)^2 +
(-1 + p1^2 + 2*p1*q1 + q1^2)*q2^2*(p3 - q3)^2 -
2*p2*q2*(p3^2*(-1 + p1^2 + 2*p1*q1 + q1^2) - 4*p1*p3*q1*q3 +
(-1 + p1^2 + 2*p1*q1 + q1^2)*q3^2)))*sigm^2)/(p1*p3*q1*q3) +
(8*b3*(b2*(p1 - q1)*(p2^2*(-1 + p1^2 + 2*p1*q1 + q1^2)*(p3 - q3)^2 +
(-1 + p1^2 + 2*p1*q1 + q1^2)*q2^2*(p3 - q3)^2 -
2*p2*q2*(p3^2*(-1 + p1^2 + 2*p1*q1 + q1^2) - 4*p1*p3*q1*q3 +
(-1 + p1^2 + 2*p1*q1 + q1^2)*q3^2)) +
2*b3*p1*q1*(p2^2*(-1 + p1^2 + 2*p1*q1 + q1^2)*(p3 - q3)^2 +
(-1 + p1^2 + 2*p1*q1 + q1^2)*q2^2*(p3 - q3)^2 -
2*p2*q2*(p3^2*(-1 + p1^2 + 2*p1*q1 + q1^2) - 2*p3*(p1 + q1)^2*q3 +
(-1 + p1^2 + 2*p1*q1 + q1^2)*q3^2)))*sigm^2)/(p1*p3*q1*q3) +
(8*b3^2*(-(p1^4*(p2^2*(p3 - q3)^2 + q2^2*(p3 - q3)^2 -
2*p2*q2*(p3^2 + q3^2))) - 2*p1*q1*(p2^2*(p3 - q3)^2 +
q2^2*(p3 - q3)^2 - 2*p2*q2*(p3^2 + q3^2)) -
q1^2*(-1 + q1^2)*(p2^2*(p3 - q3)^2 + q2^2*(p3 - q3)^2 -
2*p2*q2*(p3^2 + q3^2)) + p1^2*(p2^2*(1 + 2*q1^2)*(p3 - q3)^2 +
(1 + 2*q1^2)*q2^2*(p3 - q3)^2 - 2*p2*q2*(p3^2*(1 + 2*q1^2) -
8*p3*q1^2*q3 + (1 + 2*q1^2)*q3^2)))*sigm^2)/(p1*p3*q1*q3) +
32*sigm^4))/(2*b2^2*p2*q2 + 4*b2*b3*p2*(p1 - q1)*q2 +
2*b3^2*p2*(p1^2 + q1^2)*q2 + sigm^2)^2

4.3 Parameters for the joint distribution of $\left(T_{1|3}, T_{2|3}, T_1, T_2\right)$

$E\left(T_{1|3}\right) = \mu_{T_{1|3}} = \sqrt{n}h_{1|3} \rightarrow$
sqrt(n)*sqrt((p1*q1*(b1 + b3*(p2 - q2))^2)/(2*b2^2*p2*q2 +
4*b2*b3*p2*(p1 - q1)*q2 + 2*b3^2*p2*(p1^2 + q1^2)*q2 + sigm^2))

$Var\left(T_{1|3}\right) = \tau^2_{T_{1|3}} \rightarrow$
(16*b2*p1*p2*(b2 + b3*(p1 - q1))*q1*(b1 + b3*(p2 - q2))^2*q2*sigm^2 +
4*p1*q1*(b1 + b3*(p2 - q2))^2*sigm^4 +
4*sigm^2*(2*b2^2*p2*q2 + 4*b2*b3*p2*(p1 - q1)*q2 +
2*b3^2*p2*(p1^2 + q1^2)*q2 + sigm^2)*
(2*b3*p1*q1*(b1 + b3*(p2 - q2))*(p2 - q2) + 2*b2^2*p2*q2 +
4*b2*b3*p2*(p1 - q1)*q2 + 2*b3^2*p2*(p1^2 + q1^2)*q2 + sigm^2) +
8*b3*p1*q1*(b1 + b3*(p2 - q2))*sigm^2*
(2*b1*p2*(b2*(p1 - q1) + b3*(p1^2 + q1^2))*q2 -
(p2 - q2)*(2*b2^2*p2*q2 + 2*b2*b3*p2*(p1 - q1)*q2 + sigm^2)) -
2*(b1 + b3*(p2 - q2))*(2*b2^2*p2*(p1 - q1)*q2 + 4*b2*b3*p2*(p1^2 + q1^2)*
q2 + (p1 - q1)*(2*b3^2*p2*(p1 + q1)^2*q2 + sigm^2))*
(-2*b2*b3*p1*q1*(b1 + b3*(p2 - q2))*(p2 - q2)^2 -
(b1*(p1 - q1) + b2*(p2 - q2))*(2*b2^2*p2*q2 + 4*b2*b3*p2*(p1 - q1)*q2 +
2*b3^2*p2*(p1^2 + q1^2)*q2 + sigm^2)) -
(4*b2*b3*p2*(p1 - q1)^3*q2 + 2*b3^2*p2*(p1 - q1)^2*(p1^2 + q1^2)*q2 +
2*b2^2*p2*(p1^2 - 4*p1*q1 + q1^2)*q2 + (p1^2 - 4*p1*q1 + q1^2)*sigm^2)*
(-2*b3^2*p1*q1*(b1 + b3*(p2 - q2))^2*(p2 - q2)^2 +

(b1^2 - b3^2*(p2 - q2)^2)*(2*b2^2*p2*q2 + 4*b2*b3*p2*(p1 - q1)*q2 +
2*b3^2*p2*(p1^2 + q1^2)*q2 + sigm^2)) +
4*b2*p2*q2*(2*b2*p1*q1*(b1 + b3*(p2 - q2))*(p2 - q2) -
(p1 - q1)*(2*b2^2*p2*q2 + 4*b2*b3*p2*(p1 - q1)*q2 +
2*b3^2*p2*(p1^2 + q1^2)*q2 + sigm^2))*
(b1*(b2^2 + 2*b2*b3*(p1 - q1) + b3^2*(p1^2 + q1^2))*(p2 - q2) +
b3*(b2^2*(p2 + q2)^2 + 2*b2*b3*(p1 - q1)*(p2 + q2)^2 +
b3^2*(p1^2 + q1^2)*(p2 + q2)^2 + 2*sigm^2)) -
4*b2^2*p1*p2*q1*(b1 + b3*(p2 - q2))*q2*
(b1*(b2^2 + 2*b2*b3*(p1 - q1) + b3^2*(p1^2 + q1^2))*
(p2^2 - 4*p2*q2 + q2^2) + b3*(p2 - q2)*(b2^2*(p2^2 + q2^2) +
2*b2*b3*(p1 - q1)*(p2^2 + q2^2) + b3^2*(p1^2 + q1^2)*(p2^2 + q2^2) +
2*sigm^2)) - (2*(4*b2*b3*p1*q1*(b1 + b3*(p2 - q2))*(p2 - q2) +
(b2 + b3*(-p1 + q1))*(2*b2^2*p2*q2 + 4*b2*b3*p2*(p1 - q1)*q2 +
2*b3^2*p2*(p1^2 + q1^2)*q2 + sigm^2))*
(b3^3*(p1 - q1)*(p1^3*q1*(p2^2 - q2^2)^2 + 2*p1^4*p2*q2*(p2^2 + q2^2) +
2*p2*q1^2*(-1 + q1^2)*q2*(p2^2 + q2^2) + p1*q1*(p2 - q2)^2*
(1 + p2^2*(-2 + q1^2) + 2*p2*q1^2*q2 + (-2 + q1^2)*q2^2) +
2*p1^2*(p2^4*q1^2 + 6*p2^2*q1^2*q2^2 - p2*(1 + 2*q1^2)*q2^3 +
q1^2*q2^4 - p2^3*(q2 + 2*q1^2*q2))) +
2*b2*b3^2*(2*p1^4*p2*q2*(p2^2 + q2^2) + 2*p2*q1^2*(-1 + q1^2)*q2*
(p2^2 + q2^2) - 2*p1^2*p2*q2*(p2^2*(1 + 2*q1^2) - 8*p2*q1^2*q2 +
(1 + 2*q1^2)*q2^2) - p1^3*q1*(p2^4 + 2*p2*q2 + q2^2*(-1 + q2^2) +
p2^2*(-1 + 10*q2^2)) + p1*(-(p2^4*q1^3) + 4*p2^3*q1*q2 +
p2^2*q1^3*(1 - 10*q2^2) - q1^3*q2^2*(-1 + q2^2) +
p2*(-2*q1^3*q2 + 4*q1*q2^3))) - 4*b2*p1*p2*q1*q2*
(2*b2^2*p2*q2 + sigm^2) + b1*p1*q1*(p2 - q2)*
(-2*b2*b3*(p1^2 + q1^2)*(-1 + p2^2 + 2*p2*q2 + q2^2) +
b3^2*(p1 - q1)*(1 + p2^2*(-2 + p1^2 + 2*p1*q1 + q1^2) -
2*p2*(p1 + q1)^2*q2 + (-2 + p1^2 + 2*p1*q1 + q1^2)*q2^2) +
(p1 - q1)*sigm^2) + b3*(p1 - q1)*
(2*b2^2*p2*q2*(p2^2*(-1 + p1^2 + 2*p1*q1 + q1^2) - 12*p1*p2*q1*q2 +
(-1 + p1^2 + 2*p1*q1 + q1^2)*q2^2) +
(p2^2*(-1 + p1^2 + 3*p1*q1 + q1^2) - 6*p1*p2*q1*q2 +
(-1 + p1^2 + 3*p1*q1 + q1^2)*q2^2)*sigm^2)))/(p1*q1) +
(2*b3*(2*b3*p1*q1*(b1 + b3*(p2 - q2))*(p2 - q2) + 2*b2^2*p2*q2 +
4*b2*b3*p2*(p1 - q1)*q2 + 2*b3^2*p2*(p1^2 + q1^2)*q2 + sigm^2)*
(-2*b2^2*p1*p2*q1*(p1^2 + q1^2)*(b1 + b3*(p2 - q2))^2*(p2 - q2)*q2 +
2*b2*b3*p1*(p1 - q1)*q1*(b1 + b3*(p2 - q2))^2*(p2 - q2)*
(-1 + p2^2*q1^2 + q1^2*q2^2 + p1^2*(p2^2 + q2^2)) +
b3^2*p1*q1*(p1^2 + q1^2)*(b1 + b3*(p2 - q2))^2*(p2 - q2)*
(-1 + p2^2*q1^2 + q1^2*q2^2 + p1^2*(p2^2 + q2^2)) +
b3*(p1^2 + q1^2)*(b1 + b3*(p2 - q2))*(p2^2 - (p1^2 + q1^2)*(p2 - q2)^2 +
q2^2)*(2*b3*p1*q1*(b1 + b3*(p2 - q2))*(p2 - q2) + 2*b2^2*p2*q2 +
4*b2*b3*p2*(p1 - q1)*q2 + 2*b3^2*p2*(p1^2 + q1^2)*q2 + sigm^2) +
2*b2*p2*(p1^2 + q1^2)*(b1 + b3*(p2 - q2))*q2*
(2*b2*p1*q1*(b1 + b3*(p2 - q2))*(p2 - q2) -
(p1 - q1)*(2*b2^2*p2*q2 + 4*b2*b3*p2*(p1 - q1)*q2 +
2*b3^2*p2*(p1^2 + q1^2)*q2 + sigm^2)) +
(p1 - q1)*(b1 + b3*(p2 - q2))*(p2^2 - (p1^2 + q1^2)*(p2 - q2)^2 + q2^2)*
(4*b2*b3*p1*q1*(b1 + b3*(p2 - q2))*(p2 - q2) + (b2 + b3*(-p1 + q1))*
(2*b2^2*p2*q2 + 4*b2*b3*p2*(p1 - q1)*q2 + 2*b3^2*p2*(p1^2 + q1^2)*
q2 + sigm^2)) + 2*p1*(p1 - q1)*q1*(b1 + b3*(p2 - q2))*(p2 - q2)*
(-2*b2*b3*p1*q1*(b1 + b3*(p2 - q2))*(p2 - q2)^2 -
(b1*(p1 - q1) + b2*(p2 - q2))*(2*b2^2*p2*q2 + 4*b2*b3*p2*(p1 - q1)*
q2 + 2*b3^2*p2*(p1^2 + q1^2)*q2 + sigm^2)) +

```
p1*q1*(p1^2 + q1^2)*(p2 - q2)*(-2*b3^2*p1*q1*(b1 + b3*(p2 - q2))^2*
(p2 - q2)^2 + (b1^2 - b3^2*(p2 - q2)^2)*(2*b2^2*p2*q2 +
4*b2*b3*p2*(p1 - q1)*q2 + 2*b3^2*p2*(p1^2 + q1^2)*q2 + sigm^2)))))/
(p1*q1*(b1 + b3*(p2 - q2))) -
4*b2*b3*(-2*b2^2*p1*p2*(p1 - q1)*q1*(b1 + b3*(p2 - q2))^2*q2*
(p2^2 + q2^2) + b3^2*p1*(p1 - q1)*q1*(b1 + b3*(p2 - q2))^2*(p2^2 + q2^2)*
(-1 + p2^2*q1^2 + q1^2*q2^2 + p1^2*(p2^2 + q2^2)) +
2*b2*b3*p1*q1*(b1 + b3*(p2 - q2))^2*(p2^2 + q2^2)*
(p1^2*(-1 + p2^2 + q2^2) + q1^2*(-1 + p2^2 + q2^2) -
2*p1*q1*(p2^2 + q2^2)) + b3*(p1 - q1)*(b1 + b3*(p2 - q2))*(p2 - q2)*
(1 - (p1^2 + q1^2)*(p2^2 + q2^2))*(2*b3*p1*q1*(b1 + b3*(p2 - q2))*
(p2 - q2) + 2*b2^2*p2*q2 + 4*b2*b3*p2*(p1 - q1)*q2 +
2*b3^2*p2*(p1^2 + q1^2)*q2 + sigm^2) + 2*b2*p2*(p1 - q1)*
(b1 + b3*(p2 - q2))*(p2 - q2)*q2*(2*b2*p1*q1*(b1 + b3*(p2 - q2))*
(p2 - q2) - (p1 - q1)*(2*b2^2*p2*q2 + 4*b2*b3*p2*(p1 - q1)*q2 +
2*b3^2*p2*(p1^2 + q1^2)*q2 + sigm^2)) + (b1 + b3*(p2 - q2))*(p2 - q2)*
(p1^2 + q1^2 - (p1 - q1)^2*(p2^2 + q2^2))*
(4*b2*b3*p1*q1*(b1 + b3*(p2 - q2))*(p2 - q2) + (b2 + b3*(-p1 + q1))*
(2*b2^2*p2*q2 + 4*b2*b3*p2*(p1 - q1)*q2 + 2*b3^2*p2*(p1^2 + q1^2)*q2 +
sigm^2)) + 2*p1*q1*(b1 + b3*(p2 - q2))*(p2^2 + q2^2)*
(-2*b2*b3*p1*q1*(b1 + b3*(p2 - q2))*(p2 - q2)^2 -
(b1*(p1 - q1) + b2*(p2 - q2))*(2*b2^2*p2*q2 + 4*b2*b3*p2*(p1 - q1)*q2 +
2*b3^2*p2*(p1^2 + q1^2)*q2 + sigm^2)) + p1*(p1 - q1)*q1*(p2^2 + q2^2)*
(-2*b3^2*p1*q1*(b1 + b3*(p2 - q2))^2*(p2 - q2)^2 +
(b1^2 - b3^2*(p2 - q2)^2)*(2*b2^2*p2*q2 + 4*b2*b3*p2*(p1 - q1)*q2 +
2*b3^2*p2*(p1^2 + q1^2)*q2 + sigm^2))) -
2*b3^2*(-2*b2^2*p1*p2*q1*(p1^2 + q1^2)*(b1 + b3*(p2 - q2))^2*q2*
(p2^2 + q2^2) + 2*b2*b3*p1*(p1 - q1)*q1*(b1 + b3*(p2 - q2))^2*
(p2^2 + q2^2)*(-1 + p2^2*q1^2 + q1^2*q2^2 + p1^2*(p2^2 + q2^2)) +
b3^2*p1*q1*(p1^2 + q1^2)*(b1 + b3*(p2 - q2))^2*(p2^2 + q2^2)*
(-1 + p2^2*q1^2 + q1^2*q2^2 + p1^2*(p2^2 + q2^2)) +
b3*(p1^2 + q1^2)*(b1 + b3*(p2 - q2))*(p2 - q2)*
(1 - (p1^2 + q1^2)*(p2^2 + q2^2))*(2*b3*p1*q1*(b1 + b3*(p2 - q2))*
(p2 - q2) + 2*b2^2*p2*q2 + 4*b2*b3*p2*(p1 - q1)*q2 +
2*b3^2*p2*(p1^2 + q1^2)*q2 + sigm^2) + 2*b2*p2*(p1^2 + q1^2)*
(b1 + b3*(p2 - q2))*(p2 - q2)*q2*(2*b2*p1*q1*(b1 + b3*(p2 - q2))*
(p2 - q2) - (p1 - q1)*(2*b2^2*p2*q2 + 4*b2*b3*p2*(p1 - q1)*q2 +
2*b3^2*p2*(p1^2 + q1^2)*q2 + sigm^2)) + (p1 - q1)*(b1 + b3*(p2 - q2))*
(p2 - q2)*(1 - (p1^2 + q1^2)*(p2^2 + q2^2))*
(4*b2*b3*p1*q1*(b1 + b3*(p2 - q2))*(p2 - q2) + (b2 + b3*(-p1 + q1))*
(2*b2^2*p2*q2 + 4*b2*b3*p2*(p1 - q1)*q2 + 2*b3^2*p2*(p1^2 + q1^2)*q2 +
sigm^2)) + 2*p1*(p1 - q1)*q1*(b1 + b3*(p2 - q2))*(p2^2 + q2^2)*
(-2*b2*b3*p1*q1*(b1 + b3*(p2 - q2))*(p2 - q2)^2 -
(b1*(p1 - q1) + b2*(p2 - q2))*(2*b2^2*p2*q2 + 4*b2*b3*p2*(p1 - q1)*q2 +
2*b3^2*p2*(p1^2 + q1^2)*q2 + sigm^2)) + p1*q1*(p1^2 + q1^2)*
(p2^2 + q2^2)*(-2*b3^2*p1*q1*(b1 + b3*(p2 - q2))^2*(p2 - q2)^2 +
(b1^2 - b3^2*(p2 - q2)^2)*(2*b2^2*p2*q2 + 4*b2*b3*p2*(p1 - q1)*q2 +
2*b3^2*p2*(p1^2 + q1^2)*q2 + sigm^2))))/
(8*(2*b2^2*p2*q2 + 4*b2*b3*p2*(p1 - q1)*q2 + 2*b3^2*p2*(p1^2 + q1^2)*q2 +
sigm^2)^3)
```

$Cov\left(T_1, T_{1|3}\right) = \tau_{1|3,1} \xrightarrow{P} \left[\nabla h_{1|3}\left(\boldsymbol{\theta}\right)\right]' \Sigma \left[\nabla h_1\left(\boldsymbol{\theta}\right)\right] =$

```
((b1 + b3*(p2 - q2))*(16*b2*p1^2*p2*(b2 + b3*(p1 - q1))*q1^2*
(b1 + b3*(p2 - q2))^2*q2*sigm^2 + 4*p1^2*q1^2*(b1 + b3*(p2 - q2))^2*
sigm^4 + 4*p1*q1*sigm^2*(2*p2*((b2 + b3*p1)^2 - 2*b2*b3*q1 + b3^2*q1^2)*
q2 + sigm^2)*(2*(b3^2*p1*p2^2*q1 + b1*b3*p1*q1*(p2 - q2) +
```

```
p2*(b2 + b3*(p1 - q1))^2*q2 + b3^2*p1*q1*q2^2) + sigm^2) -
4*b2*p1*p2*q1*q2*(((b2 + b3*p1)^2 - 2*b2*b3*q1 + b3^2*q1^2)*
(b1*(p2 - q2) + b3*(p2 + q2)^2) + 2*b3*sigm^2)*
(-2*b2*b3*p1*p2^2*q1 + 2*p2*(b2 + b3*(p1 - q1))*
(b2*(p1 - q1) + b3*(p1^2 + q1^2))*q2 - 2*b2*b3*p1*q1*q2^2 +
2*b1*b2*p1*q1*(-p2 + q2) + (p1 - q1)*sigm^2) -
4*b2^2*p1^2*p2*q1^2*(b1 + b3*(p2 - q2))*q2*
(((b2 + b3*p1)^2 - 2*b2*b3*q1 + b3^2*q1^2)*(b3*(p2 - q2)*(p2^2 + q2^2) +
b1*(p2^2 - 4*p2*q2 + q2^2)) + 2*b3*(p2 - q2)*sigm^2) +
8*b3*p1^2*q1^2*(b1 + b3*(p2 - q2))*sigm^2*
(2*b1*p2*(b2*(p1 - q1) + b3*(p1^2 + q1^2))*q2 -
(p2 - q2)*(2*b2*p2*(b2 + b3*(p1 - q1))*q2 + sigm^2)) -
p1*q1*(b1 + b3*(p2 - q2))*(2*p2*(b2 + b3*(p1 - q1))*
(b3*(p1 - q1)*(p1^2 + q1^2) + b2*(p1^2 - 4*p1*q1 + q1^2))*q2 +
(p1^2 - 4*p1*q1 + q1^2)*sigm^2)*
(b1*(2*b2^2*p2*q2 + 4*b2*b3*p2*(p1 - q1)*q2 +
2*b3^2*(-(p1*p2^2*q1) + p2*(p1 + q1)^2*q2 - p1*q1*q2^2) + sigm^2) -
b3*(p2 - q2)*(2*(b3^2*p1*p2^2*q1 + p2*(b2 + b3*(p1 - q1))^2*q2 +
b3^2*p1*q1*q2^2) + sigm^2)) +
2*(4*b2*b3*p1*q1*(b1 + b3*(p2 - q2))*(p2 - q2) +
(b2 + b3*(-p1 + q1))*(2*p2*((b2 + b3*p1)^2 - 2*b2*b3*q1 + b3^2*q1^2)*
q2 + sigm^2))*(2*b2*b3^2*(p1*p2^2*(-1 + p2^2)*q1*(p1^2 + q1^2) -
2*p2*(p1^4*p2^2 - p1^3*q1 + p2^2*q1^2*(-1 + q1^2) -
p1^2*p2^2*(1 + 2*q1^2) + p1*(2*p2^2*q1 - q1^3))*q2 +
p1*q1*(p1^2*(-1 + 10*p2^2) - 16*p1*p2^2*q1 + (-1 + 10*p2^2)*q1^2)*
q2^2 - 2*p2*(p1 - q1)^2*(-1 + p1 + q1)*(1 + p1 + q1)*q2^3 +
p1*q1*(p1^2 + q1^2)*q2^4) - b3^3*(p1 - q1)*
(p1*p2^2*q1*(1 + p2^2*(-2 + (p1 + q1)^2)) +
2*p2*(p1^4*p2^2 + p1*(-1 + 2*p2^2)*q1 + p2^2*q1^2*(-1 + q1^2) -
p1^2*p2^2*(1 + 2*q1^2))*q2 -
p1*q1*(-1 + 2*p2^2*(2 + p1^2 - 6*p1*q1 + q1^2))*q2^2 +
2*p2*(p1 - q1)^2*(-1 + p1 + q1)*(1 + p1 + q1)*q2^3 +
p1*q1*(-2 + (p1 + q1)^2)*q2^4) + 4*b2*p1*p2*q1*q2*
(2*b2^2*p2*q2 + sigm^2) - b1*p1*q1*(p2 - q2)*
(-2*b2*b3*(p1^2 + q1^2)*(-1 + p2 + q2)*(1 + p2 + q2) +
b3^2*(p1 - q1)*(1 + p2^2*(-2 + (p1 + q1)^2) - 2*p2*(p1 + q1)^2*q2 +
(-2 + (p1 + q1)^2)*q2^2) + (p1 - q1)*sigm^2) -
b3*(p1 - q1)*(2*b2^2*p2*q2*(p2^2*(-1 + p1 + q1)*(1 + p1 + q1) -
12*p1*p2*q1*q2 + (-1 + p1 + q1)*(1 + p1 + q1)*q2^2) +
(p2^2*(-1 + p1^2 + 3*p1*q1 + q1^2) - 6*p1*p2*q1*q2 +
(-1 + p1^2 + 3*p1*q1 + q1^2)*q2^2)*sigm^2)) +
2*b3*(2*(b3^2*p1*p2^2*q1 + b1*b3*p1*q1*(p2 - q2) +
p2*(b2 + b3*(p1 - q1))^2*q2 + b3^2*p1*q1*q2^2) + sigm^2)*
(-2*b2^3*p2*(p1 - q1)*q2*(p2^2*(-1 + p1 + q1)*(1 + p1 + q1) -
4*p1*p2*q1*q2 + (-1 + p1 + q1)*(1 + p1 + q1)*q2^2) -
4*b2^2*b3*p2*q2*(p2^2*(p1^4 + p1^3*q1 - q1^2 + q1^4 -
p1^2*(1 + 2*q1^2) + p1*(q1 + q1^3)) -
2*p1*p2*q1*(3*p1^2 - 4*p1*q1 + 3*q1^2)*q2 +
(p1^4 + p1^3*q1 - q1^2 + q1^4 - p1^2*(1 + 2*q1^2) + p1*(q1 + q1^3))*
q2^2) - b1*p1*q1*(p2 - q2)*(-8*b2^2*p1*p2*q1*q2 +
2*b2*b3*(p1 - q1)*(1 + p2^2*(-2 + (p1 + q1)^2) -
2*p2*(p1^2 + 6*p1*q1 + q1^2)*q2 + (-2 + (p1 + q1)^2)*q2^2) +
b3^2*(p1^2 + q1^2)*(1 + p2^2*(-2 + (p1 + q1)^2) -
2*p2*(p1^2 + 6*p1*q1 + q1^2)*q2 + (-2 + (p1 + q1)^2)*q2^2) +
(p1^2 - 4*p1*q1 + q1^2)*sigm^2) - b2*(p1 - q1)*
(2*b3^2*(p1*p2^2*q1*(1 + p2^2*(-2 + (p1 + q1)^2)) +
```

p2*(p1^2*(-1 + p1^2)*p2^2 + 2*p1*(-1 + p1^2*p2^2)*q1 -
(1 + 6*p1^2)*p2^2*q1^2 + 2*p1*p2^2*q1^3 + p2^2*q1^4)*q2 -
p1*(-1 + 2*p2^2*(2 + 3*(p1 - q1)^2))*q1*q2^2 +
p2*(p1^4 + 2*p1^3*q1 - q1^2 + 2*p1*q1^3 + q1^4 - p1^2*(1 + 6*q1^2))*
q2^3 + p1*q1*(-2 + (p1 + q1)^2)*q2^4) +
(p2^2*(-1 + p1 + q1)*(1 + p1 + q1) - 4*p1*p2*q1*q2 +
(-1 + p1 + q1)*(1 + p1 + q1)*q2^2)*sigm^2) -
b3*p1*q1*(b3^2*(p1^2 + q1^2)*(p2^4*(-2 + (p1 + q1)^2) -
8*p1*p2^3*q1*q2 + q2^2 + (-2 + (p1 + q1)^2)*q2^4 +
p2^2*(1 - 2*(2 + p1^2 - 6*p1*q1 + q1^2)*q2^2) -
2*p2*(q2 + 4*p1*q1*q2^3)) + (p2^2*(-2 + 3*p1^2 + 3*q1^2) -
6*p2*(p1^2 + q1^2)*q2 + (-2 + 3*p1^2 + 3*q1^2)*q2^2)*sigm^2)) +
2*p1*q1*(b1 + b3*(p2 - q2))*(2*p2*(b2 + b3*(p1 - q1))*
(b2*(p1 - q1) + b3*(p1 + q1)^2)*q2 + (p1 - q1)*sigm^2)*
(b2*(p2 - q2)*(2*(b3^2*p1*p2^2*q1 + p2*(b2 + b3*(p1 - q1))^2*q2 +
b3^2*p1*q1*q2^2) + sigm^2) + b1*(2*b2^2*p2*(p1 - q1)*q2 +
2*b2*b3*(p1*p2^2*q1 + 2*p2*(p1^2 - 3*p1*q1 + q1^2)*q2 + p1*q1*q2^2) +
(p1 - q1)*(2*b3^2*p2*(p1^2 + q1^2)*q2 + sigm^2))) -
4*b2*b3*p1*q1*(b1 + b3*(p2 - q2))*
(b1*p1*q1*(-2*b2*b3*(p1^2 + q1^2)*(p2^4 - 2*p2^3*q2 - q2^2 + q2^4 -
2*p2*q2*(-2 + q2^2) + p2^2*(-1 + 2*q2^2)) +
b3^2*(p1 - q1)*(-(p2^4*(p1 + q1)^2) + 2*p2^3*(p1 + q1)^2*q2 + q2^2 -
(p1 + q1)^2*q2^4 + p2^2*(1 - 2*(p1 + q1)^2*q2^2) +
2*p2*q2*(-2 + (p1 + q1)^2*q2^2)) - (p1 - q1)*(8*b2^2*p2^2*q2^2 +
(p2^2 + q2^2)*sigm^2)) - (p2 - q2)*
(b3^3*(p1 - q1)*(p1*p2^2*q1*(-1 + p2*(p1 + q1))*(1 + p2*(p1 + q1)) +
2*p2*(p1^4 + p1^3*p2^2*q1 - q1^2 + q1^4 + p1*q1*(2 + p2^2*q1^2) +
p1^2*(-1 - 2*(-1 + p2^2)*q1^2))*q2 +
p1*q1*(-1 + 2*p2^2*(p1 + q1)^2)*q2^2 + 2*p1*p2*(p1 - q1)^2*q1*q2^3 +
p1*q1*(p1 + q1)^2*q2^4) + 2*b2*b3^2*(p1*p2^2*(-1 + p2^2)*q1*
(p1^2 + q1^2) + p2*(p1^4*(1 + p2^2) + 2*p1^3*p2^2*q1 - 2*q1^2 +
(1 + p2^2)*q1^4 + 2*p1*q1*(2 + p2^2*q1^2) +
p1^2*(-2 + (2 - 6*p2^2)*q1^2))*q2 + (p1^2 + q1^2)*
(2*p1^2*p2^2 + 2*p2^2*q1^2 - p1*(q1 + 2*p2^2*q1))*q2^2 +
p2*(p1 - q1)^2*(p1^2 + 4*p1*q1 + q1^2)*q2^3 + p1*q1*(p1^2 + q1^2)*
q2^4) + b2*(-4*p1*p2*q1*q2 + p1^2*(-1 + p2 + q2)*(1 + p2 + q2) +
q1^2*(-1 + p2 + q2)*(1 + p2 + q2))*(2*b2^2*p2*q2 + sigm^2) +
b3*(p1 - q1)*(2*b2^2*p2*q2*(-1 + 2*p1*q1*(p2 - q2)^2 +
p1^2*(-1 + 2*(p2 + q2)^2) + q1^2*(-1 + 2*(p2 + q2)^2)) +
(-1 + p1^2 + q1^2 + 3*p1*q1*(p2^2 + q2^2))*sigm^2))) +
2*b3^2*p1*q1*(b1 + b3*(p2 - q2))*
(b1*p1*q1*(8*b2^2*p2*q2*(-(p2*q1) + p1*q2)*(p1*p2 - q1*q2) +
2*b2*b3*(p1 - q1)*(p2^4*(p1 + q1)^2 + 4*p2*q2 -
2*p2^3*(p1^2 + 6*p1*q1 + q1^2)*q2 - q2^2 -
2*p2*(p1^2 + 6*p1*q1 + q1^2)*q2^3 + (p1 + q1)^2*q2^4 +
p2^2*(-1 + 2*(p1 + q1)^2*q2^2)) + b3^2*(p1^2 + q1^2)*
(p2^4*(p1 + q1)^2 + 4*p2*q2 - 2*p2^3*(p1^2 + 6*p1*q1 + q1^2)*q2 -
q2^2 - 2*p2*(p1^2 + 6*p1*q1 + q1^2)*q2^3 + (p1 + q1)^2*q2^4 +
p2^2*(-1 + 2*(p1 + q1)^2*q2^2)) + (p1^2 - 4*p1*q1 + q1^2)*
(p2^2 + q2^2)*sigm^2) + (p2 - q2)*
(2*b2^3*p2*(p1 - q1)*q2*(-1 + p1^2*(p2 + q2)^2 + q1^2*(p2 + q2)^2 +
2*p1*q1*(p2^2 + q2^2)) + 4*b2^2*b3*p2*q2*
(-(p1^2*(1 + 2*q1^2*(p2 - q2)^2)) + p1*(q1 + q1^3*(p2 - q2)^2) +
p1^3*q1*(p2 - q2)^2 + p1^4*(p2 + q2)^2 + q1^2*(-1 + q1*(p2 + q2))*
(1 + q1*(p2 + q2))) + b2*(p1 - q1)*
(2*b3^2*(p1^4*p2*q2*(p2 + q2)^2 + p1^3*q1*(p2^2 + q2^2)*

(p2^2 + 4*p2*q2 + q2^2) + p2*q1^2*q2*(-1 + q1*(p2 + q2))*
(1 + q1*(p2 + q2)) + p1*q1*(p2^2 + q2^2)*
(-1 + q1^2*(p2^2 + 4*p2*q2 + q2^2)) +
p1^2*(2*p2^4*q1^2 - 2*p2^3*q1^2*q2 + 8*p2^2*q1^2*q2^2 +
2*q1^2*q2^4 - p2*(q2 + 2*q1^2*q2^3))) +
(-1 + p2^2*(p1 + q1)^2 + 2*p2*(p1^2 + q1^2)*q2 + (p1 + q1)^2*q2^2)*
sigm^2) + b3*p1*q1*(-2*sigm^2 + (p1^2 + q1^2)*(p2^2 + q2^2)*
(b3^2*(-1 + p2^2*(p1 + q1)^2 + 2*p2*(p1 - q1)^2*q2 +
(p1 + q1)^2*q2^2) + 3*sigm^2))))))/
(4*sqrt(B)*sqrt((p1*q1*(b1 + b3*(p2 - q2))^2)/
(2*p2*((b2 + b3*p1)^2 - 2*b2*b3*q1 + b3^2*q1^2)*q2 + sigm^2))*
(2*p2*((b2 + b3*p1)^2 - 2*b2*b3*q1 + b3^2*q1^2)*q2 + sigm^2)^3*
sqrt(p1*q1*(2*p2*((b2 + b3*p1)^2 - 2*b2*b3*q1 + b3^2*q1^2)*q2 + sigm^2)))

$Cov\left(T_{2|3}, T_{1|3}\right) = \tau_{1|3,2|3} \xrightarrow{P} \left[\nabla h_{1|3}\left(\boldsymbol{\theta}\right)\right]' \Sigma \left[\nabla h_{2|3}\left(\boldsymbol{\theta}\right)\right] =$
(2*p1*p2*(b2 + b3*(p1 - q1))^2*q1*(b1 + b3*(p2 - q2))^2*q2*sigm^4 -
4*b1*p1*p2*(b2 + b3*(p1 - q1))^2*q1*(b1 + b3*(p2 - q2))*q2*sigm^2*
(2*b2^2*p2*q2 + 4*b2*b3*p2*(p1 - q1)*q2 + 2*b3^2*p2*(p1^2 + q1^2)*q2 +
sigm^2) - 4*p1*p2*(b2 + b3*(p1 - q1))^2*q1*(b1 + b3*(p2 - q2))^2*q2*
sigm^2*(2*b1^2*p1*q1 + 4*b1*b3*p1*q1*(p2 - q2) + 2*b2*b3*p2*(p1 - q1)*q2 +
2*b3^2*(p1*q1*(p2 - q2)^2 + p1^2*p2*q2 + p2*q1^2*q2) + sigm^2) +
b1^2*p1*p2*(b2 + b3*(p1 - q1))^2*q1*(b1 + b3*(p2 - q2))^2*q2*
(4*b2*b3*p2*(p1 - q1)^3*q2 + 2*b3^2*p2*(p1 - q1)^2*(p1^2 + q1^2)*q2 +
2*b2^2*p2*(p1^2 - 4*p1*q1 + q1^2)*q2 + (p1^2 - 4*p1*q1 + q1^2)*sigm^2) +
4*b3*p1*p2*(b2 + b3*(p1 - q1))^2*q1*(b1 + b3*(p2 - q2))*q2*sigm^2*
(2*b1*p2*(b2*(p1 - q1) + b3*(p1^2 + q1^2))*q2 -
(p2 - q2)*(2*b2^2*p2*q2 + 2*b2*b3*p2*(p1 - q1)*q2 + sigm^2)) +
b1*p1*(b2 + b3*(p1 - q1))*q1*(b1 + b3*(p2 - q2))^2*
(2*b2^2*p2*(p1 - q1)*q2 + 4*b2*b3*p2*(p1^2 + q1^2)*q2 +
(p1 - q1)*(2*b3^2*p2*(p1 + q1)^2*q2 + sigm^2))*
(2*b1^2*p1*q1*(p2 - q2) - 2*b1*(b2*p2*(p1 - q1)*q2 +
b3*(p1^2*p2*q2 + p2*q1^2*q2 - 2*p1*q1*(p2^2 - p2*q2 + q2^2))) +
(p2 - q2)*(2*b3^2*p1*q1*(p2^2 + q2^2) + sigm^2)) +
2*p1*p2*(b2 + b3*(p1 - q1))*q1*(b1 + b3*(p2 - q2))*q2*
(-2*b1*b3*p2*(b2 + b3*(p1 - q1))*(p1 - q1)^2*q2 -
(b1*(p1 - q1) + b2*(p2 - q2))*(2*b1^2*p1*q1 + 4*b1*b3*p1*q1*(p2 - q2) +
2*b3^2*p1*q1*(p2^2 + q2^2) + sigm^2))*
(b1*(b2^2 + 2*b2*b3*(p1 - q1) + b3^2*(p1^2 + q1^2))*(p2 - q2) +
b3*(b2^2*(p2 + q2)^2 + 2*b2*b3*(p1 - q1)*(p2 + q2)^2 +
b3^2*(p1^2 + q1^2)*(p2 + q2)^2 + 2*sigm^2)) +
p1*p2*q1*(b1 + b3*(p2 - q2))*q2*(-2*b3^2*p2*(b2 + b3*(p1 - q1))^2*
(p1 - q1)^2*q2 + (b2^2 - b3^2*(p1 - q1)^2)*(2*b1^2*p1*q1 +
4*b1*b3*p1*q1*(p2 - q2) + 2*b3^2*p1*q1*(p2^2 + q2^2) + sigm^2))*
(b1*(b2^2 + 2*b2*b3*(p1 - q1) + b3^2*(p1^2 + q1^2))*
(p2^2 - 4*p2*q2 + q2^2) + b3*(p2 - q2)*(b2^2*(p2^2 + q2^2) +
2*b2*b3*(p1 - q1)*(p2^2 + q2^2) + b3^2*(p1^2 + q1^2)*(p2^2 + q2^2) +
2*sigm^2)) - (b2 + b3*(p1 - q1))*(b1 + b3*(p2 - q2))*
(4*b1*b3*p2*(b2 + b3*(p1 - q1))*(p1 - q1)*q2 +
(b1 + b3*(-p2 + q2))*(2*b1^2*p1*q1 + 4*b1*b3*p1*q1*(p2 - q2) +
2*b3^2*p1*q1*(p2^2 + q2^2) + sigm^2))*
(b3^3*(p1 - q1)*(p1^3*q1*(p2^2 - q2^2)^2 + 2*p1^4*p2*q2*(p2^2 + q2^2) +
2*p2*q1^2*(-1 + q1^2)*q2*(p2^2 + q2^2) + p1*q1*(p2 - q2)^2*
(1 + p2^2*(-2 + q1^2) + 2*p2*q1^2*q2 + (-2 + q1^2)*q2^2) +
2*p1^2*(p2^4*q1^2 + 6*p2^2*q1^2*q2^2 - p2*(1 + 2*q1^2)*q2^3 +
q1^2*q2^4 - p2^3*(q2 + 2*q1^2*q2))) +
2*b2*b3^2*(2*p1^4*p2*q2*(p2^2 + q2^2) + 2*p2*q1^2*(-1 + q1^2)*q2*

(p2^2 + q2^2) - 2*p1^2*p2*q2*(p2^2*(1 + 2*q1^2) - 8*p2*q1^2*q2 +
(1 + 2*q1^2)*q2^2) - p1^3*q1*(p2^4 + 2*p2*q2 + q2^2*(-1 + q2^2) +
p2^2*(-1 + 10*q2^2)) + p1*(-(p2^4*q1^3) + 4*p2^3*q1*q2 +
p2^2*q1^3*(1 - 10*q2^2) - q1^3*q2^2*(-1 + q2^2) +
p2*(-2*q1^3*q2 + 4*q1*q2^3))) - 4*b2*p1*p2*q1*q2*
(2*b2^2*p2*q2 + sigm^2) + b1*p1*q1*(p2 - q2)*
(-2*b2*b3*(p1^2 + q1^2)*(-1 + p2^2 + 2*p2*q2 + q2^2) +
b3^2*(p1 - q1)*(1 + p2^2*(-2 + p1^2 + 2*p1*q1 + q1^2) -
2*p2*(p1 + q1)^2*q2 + (-2 + p1^2 + 2*p1*q1 + q1^2)*q2^2) +
(p1 - q1)*sigm^2) + b3*(p1 - q1)*
(2*b2^2*p2*q2*(p2^2*(-1 + p1^2 + 2*p1*q1 + q1^2) - 12*p1*p2*q1*q2 +
(-1 + p1^2 + 2*p1*q1 + q1^2)*q2^2) +
(p2^2*(-1 + p1^2 + 3*p1*q1 + q1^2) - 6*p1*p2*q1*q2 +
(-1 + p1^2 + 3*p1*q1 + q1^2)*q2^2)*sigm^2)) -
2*b1*b3*p2*(b2 + b3*(p1 - q1))^2*q2*
(-2*b2^2*p1*p2*q1*(p1^2 + q1^2)*(b1 + b3*(p2 - q2))^2*(p2 - q2)*q2 +
2*b2*b3*p1*(p1 - q1)*q1*(b1 + b3*(p2 - q2))^2*(p2 - q2)*
(-1 + p2^2*q1^2 + q1^2*q2^2 + p1^2*(p2^2 + q2^2)) +
b3^2*p1*q1*(p1^2 + q1^2)*(b1 + b3*(p2 - q2))^2*(p2 - q2)*
(-1 + p2^2*q1^2 + q1^2*q2^2 + p1^2*(p2^2 + q2^2)) +
b3*(p1^2 + q1^2)*(b1 + b3*(p2 - q2))*(p2^2 - (p1^2 + q1^2)*(p2 - q2)^2 +
q2^2)*(2*b3*p1*q1*(b1 + b3*(p2 - q2))*(p2 - q2) + 2*b2^2*p2*q2 +
4*b2*b3*p2*(p1 - q1)*q2 + 2*b3^2*p2*(p1^2 + q1^2)*q2 + sigm^2) +
2*b2*p2*(p1^2 + q1^2)*(b1 + b3*(p2 - q2))*q2*
(2*b2*p1*q1*(b1 + b3*(p2 - q2))*(p2 - q2) -
(p1 - q1)*(2*b2^2*p2*q2 + 4*b2*b3*p2*(p1 - q1)*q2 +
2*b3^2*p2*(p1^2 + q1^2)*q2 + sigm^2)) + (p1 - q1)*(b1 + b3*(p2 - q2))*
(p2^2 - (p1^2 + q1^2)*(p2 - q2)^2 + q2^2)*
(4*b2*b3*p1*q1*(b1 + b3*(p2 - q2))*(p2 - q2) + (b2 + b3*(-p1 + q1))*
(2*b2^2*p2*q2 + 4*b2*b3*p2*(p1 - q1)*q2 + 2*b3^2*p2*(p1^2 + q1^2)*q2 +
sigm^2)) + 2*p1*(p1 - q1)*q1*(b1 + b3*(p2 - q2))*(p2 - q2)*
(-2*b2*b3*p1*q1*(b1 + b3*(p2 - q2))*(p2 - q2)^2 -
(b1*(p1 - q1) + b2*(p2 - q2))*(2*b2^2*p2*q2 + 4*b2*b3*p2*(p1 - q1)*q2 +
2*b3^2*p2*(p1^2 + q1^2)*q2 + sigm^2)) + p1*q1*(p1^2 + q1^2)*(p2 - q2)*
(-2*b3^2*p1*q1*(b1 + b3*(p2 - q2))^2*(p2 - q2)^2 +
(b1^2 - b3^2*(p2 - q2)^2)*(2*b2^2*p2*q2 + 4*b2*b3*p2*(p1 - q1)*q2 +
2*b3^2*p2*(p1^2 + q1^2)*q2 + sigm^2))) +
b3*(b2 + b3*(p1 - q1))*(2*b1^2*p1*q1 + 4*b1*b3*p1*q1*(p2 - q2) +
2*b3*p2*(b2 + b3*(p1 - q1))*(p1 - q1)*q2 + 2*b3^2*p1*q1*(p2^2 + q2^2) +
sigm^2)*(-2*b2^2*p1*p2*(p1 - q1)*q1*(b1 + b3*(p2 - q2))^2*q2*
(p2^2 + q2^2) + b3^2*p1*(p1 - q1)*q1*(b1 + b3*(p2 - q2))^2*(p2^2 + q2^2)*
(-1 + p2^2*q1^2 + q1^2*q2^2 + p1^2*(p2^2 + q2^2)) +
2*b2*b3*p1*q1*(b1 + b3*(p2 - q2))^2*(p2^2 + q2^2)*
(p1^2*(-1 + p2^2 + q2^2) + q1^2*(-1 + p2^2 + q2^2) -
2*p1*q1*(p2^2 + q2^2)) + b3*(p1 - q1)*(b1 + b3*(p2 - q2))*(p2 - q2)*
(1 - (p1^2 + q1^2)*(p2^2 + q2^2))*(2*b3*p1*q1*(b1 + b3*(p2 - q2))*
(p2 - q2) + 2*b2^2*p2*q2 + 4*b2*b3*p2*(p1 - q1)*q2 +
2*b3^2*p2*(p1^2 + q1^2)*q2 + sigm^2) + 2*b2*p2*(p1 - q1)*
(b1 + b3*(p2 - q2))*(p2 - q2)*q2*(2*b2*p1*q1*(b1 + b3*(p2 - q2))*
(p2 - q2) - (p1 - q1)*(2*b2^2*p2*q2 + 4*b2*b3*p2*(p1 - q1)*q2 +
2*b3^2*p2*(p1^2 + q1^2)*q2 + sigm^2)) + (b1 + b3*(p2 - q2))*(p2 - q2)*
(p1^2 + q1^2 - (p1 - q1)^2*(p2^2 + q2^2))*
(4*b2*b3*p1*q1*(b1 + b3*(p2 - q2))*(p2 - q2) + (b2 + b3*(-p1 + q1))*
(2*b2^2*p2*q2 + 4*b2*b3*p2*(p1 - q1)*q2 + 2*b3^2*p2*(p1^2 + q1^2)*q2 +
sigm^2)) + 2*p1*q1*(b1 + b3*(p2 - q2))*(p2^2 + q2^2)*
(-2*b2*b3*p1*q1*(b1 + b3*(p2 - q2))*(p2 - q2)^2 -

(b1*(p1 - q1) + b2*(p2 - q2))*(2*b2^2*p2*q2 + 4*b2*b3*p2*(p1 - q1)*q2 +
2*b3^2*p2*(p1^2 + q1^2)*q2 + sigm^2)) + p1*(p1 - q1)*q1*(p2^2 + q2^2)*
(-2*b3^2*p1*q1*(b1 + b3*(p2 - q2))^2*(p2 - q2)^2 +
(b1^2 - b3^2*(p2 - q2)^2)*(2*b2^2*p2*q2 + 4*b2*b3*p2*(p1 - q1)*q2 +
2*b3^2*p2*(p1^2 + q1^2)*q2 + sigm^2))) -
b3^2*p2*(b2 + b3*(p1 - q1))^2*q2*(-2*b2^2*p1*p2*q1*(p1^2 + q1^2)*
(b1 + b3*(p2 - q2))^2*q2*(p2^2 + q2^2) + 2*b2*b3*p1*(p1 - q1)*q1*
(b1 + b3*(p2 - q2))^2*(p2^2 + q2^2)*(-1 + p2^2*q1^2 + q1^2*q2^2 +
p1^2*(p2^2 + q2^2)) + b3^2*p1*q1*(p1^2 + q1^2)*(b1 + b3*(p2 - q2))^2*
(p2^2 + q2^2)*(-1 + p2^2*q1^2 + q1^2*q2^2 + p1^2*(p2^2 + q2^2)) +
b3*(p1^2 + q1^2)*(b1 + b3*(p2 - q2))*(p2 - q2)*
(1 - (p1^2 + q1^2)*(p2^2 + q2^2))*(2*b3*p1*q1*(b1 + b3*(p2 - q2))*
(p2 - q2) + 2*b2^2*p2*q2 + 4*b2*b3*p2*(p1 - q1)*q2 +
2*b3^2*p2*(p1^2 + q1^2)*q2 + sigm^2) + 2*b2*p2*(p1^2 + q1^2)*
(b1 + b3*(p2 - q2))*(p2 - q2)*q2*(2*b2*p1*q1*(b1 + b3*(p2 - q2))*
(p2 - q2) - (p1 - q1)*(2*b2^2*p2*q2 + 4*b2*b3*p2*(p1 - q1)*q2 +
2*b3^2*p2*(p1^2 + q1^2)*q2 + sigm^2)) + (p1 - q1)*(b1 + b3*(p2 - q2))*
(p2 - q2)*(1 - (p1^2 + q1^2)*(p2^2 + q2^2))*
(4*b2*b3*p1*q1*(b1 + b3*(p2 - q2))*(p2 - q2) + (b2 + b3*(-p1 + q1))*
(2*b2^2*p2*q2 + 4*b2*b3*p2*(p1 - q1)*q2 + 2*b3^2*p2*(p1^2 + q1^2)*q2 +
sigm^2)) + 2*p1*(p1 - q1)*q1*(b1 + b3*(p2 - q2))*(p2^2 + q2^2)*
(-2*b2*b3*p1*q1*(b1 + b3*(p2 - q2))*(p2 - q2)^2 -
(b1*(p1 - q1) + b2*(p2 - q2))*(2*b2^2*p2*q2 + 4*b2*b3*p2*(p1 - q1)*q2 +
2*b3^2*p2*(p1^2 + q1^2)*q2 + sigm^2)) + p1*q1*(p1^2 + q1^2)*
(p2^2 + q2^2)*(-2*b3^2*p1*q1*(b1 + b3*(p2 - q2))^2*(p2 - q2)^2 +
(b1^2 - b3^2*(p2 - q2)^2)*(2*b2^2*p2*q2 + 4*b2*b3*p2*(p1 - q1)*q2 +
2*b3^2*p2*(p1^2 + q1^2)*q2 + sigm^2))))/
(4*sqrt((p1*q1*(b1 + b3*(p2 - q2))^2)/(2*b2^2*p2*q2 +
4*b2*b3*p2*(p1 - q1)*q2 + 2*b3^2*p2*(p1^2 + q1^2)*q2 + sigm^2))*
(2*b2^2*p2*q2 + 4*b2*b3*p2*(p1 - q1)*q2 + 2*b3^2*p2*(p1^2 + q1^2)*q2 +
sigm^2)^2*sqrt((p2*(b2 + b3*(p1 - q1))^2*q2)/
(2*b1^2*p1*q1 + 4*b1*b3*p1*q1*(p2 - q2) + 2*b3^2*p1*q1*(p2^2 + q2^2) +
sigm^2))*(2*b1^2*p1*q1 + 4*b1*b3*p1*q1*(p2 - q2) +
2*b3^2*p1*q1*(p2^2 + q2^2) + sigm^2)^2)

$Cov\left(T_1, T_{2|3}\right) = \tau_{2|3,1} \xrightarrow{P} \left[\nabla h_{2|3}\left(\boldsymbol{\theta}\right)\right]' \Sigma \left[\nabla h_1\left(\boldsymbol{\theta}\right)\right] =$
covT1T2l3 =
(p1*q1*(2*p1*p2*(b2 + b3*(p1 - q1))^2*q1*(b1 + b3*(p2 - q2))*q2*sigm^4 -
4*b1*p1*p2*(b2 + b3*(p1 - q1))^2*q1*q2*sigm^2*
(2*b2^2*p2*q2 + 4*b2*b3*p2*(p1 - q1)*q2 + 2*b3^2*p2*(p1^2 + q1^2)*q2 +
sigm^2) - 4*p1*p2*(b2 + b3*(p1 - q1))^2*q1*(b1 + b3*(p2 - q2))*q2*sigm^2*
(2*b1^2*p1*q1 + 4*b1*b3*p1*q1*(p2 - q2) + 2*b2*b3*p2*(p1 - q1)*q2 +
2*b3^2*(p1*q1*(p2 - q2)^2 + p1^2*p2*q2 + p2*q1^2*q2) + sigm^2) +
b1^2*p1*p2*(b2 + b3*(p1 - q1))^2*q1*(b1 + b3*(p2 - q2))*q2*
(4*b2*b3*p2*(p1 - q1)^3*q2 + 2*b3^2*p2*(p1 - q1)^2*(p1^2 + q1^2)*q2 +
2*b2^2*p2*(p1^2 - 4*p1*q1 + q1^2)*q2 + (p1^2 - 4*p1*q1 + q1^2)*sigm^2) +
4*b3*p1*p2*(b2 + b3*(p1 - q1))^2*q1*q2*sigm^2*
(2*b1*p2*(b2*(p1 - q1) + b3*(p1^2 + q1^2))*q2 -
(p2 - q2)*(2*b2^2*p2*q2 + 2*b2*b3*p2*(p1 - q1)*q2 + sigm^2)) -
b1*p1*(b2 + b3*(p1 - q1))*q1*(b1 + b3*(p2 - q2))*
(2*b2^2*p2*(p1 - q1)*q2 + 4*b2*b3*p2*(p1^2 + q1^2)*q2 +
(p1 - q1)*(2*b3^2*p2*(p1 + q1)^2*q2 + sigm^2))*
(2*b1*p2*(b2 + b3*(p1 - q1))*(p1 - q1)*q2 -
(p2 - q2)*(2*b1^2*p1*q1 + 4*b1*b3*p1*q1*(p2 - q2) +
2*b3^2*p1*q1*(p2^2 + q2^2) + sigm^2)) + 2*p1*p2*(b2 + b3*(p1 - q1))*q1*
q2*(-2*b1*b3*p2*(b2 + b3*(p1 - q1))*(p1 - q1)^2*q2 -

```
(b1*(p1 - q1) + b2*(p2 - q2))*(2*b1^2*p1*q1 + 4*b1*b3*p1*q1*(p2 - q2) +
2*b3^2*p1*q1*(p2^2 + q2^2) + sigm^2))*
(b1*(b2^2 + 2*b2*b3*(p1 - q1) + b3^2*(p1^2 + q1^2))*(p2 - q2) +
b3*(b2^2*(p2 + q2)^2 + 2*b2*b3*(p1 - q1)*(p2 + q2)^2 +
b3^2*(p1^2 + q1^2)*(p2 + q2)^2 + 2*sigm^2)) +
p1*p2*q1*q2*(-2*b3^2*p2*(b2 + b3*(p1 - q1))^2*(p1 - q1)^2*q2 +
(b2^2 - b3^2*(p1 - q1)^2)*(2*b1^2*p1*q1 + 4*b1*b3*p1*q1*(p2 - q2) +
2*b3^2*p1*q1*(p2^2 + q2^2) + sigm^2))*
(b1*(b2^2 + 2*b2*b3*(p1 - q1) + b3^2*(p1^2 + q1^2))*
(p2^2 - 4*p2*q2 + q2^2) + b3*(p2 - q2)*(b2^2*(p2^2 + q2^2) +
2*b2*b3*(p1 - q1)*(p2^2 + q2^2) + b3^2*(p1^2 + q1^2)*(p2^2 + q2^2) +
2*sigm^2)) + (b2 + b3*(p1 - q1))*
(4*b1*b3*p2*(b2 + b3*(p1 - q1))*(p1 - q1)*q2 + (b1 + b3*(-p2 + q2))*
(2*b1^2*p1*q1 + 4*b1*b3*p1*q1*(p2 - q2) + 2*b3^2*p1*q1*(p2^2 + q2^2) +
sigm^2))*(-(b3^3*(p1 - q1)*(p1^3*q1*(p2^2 - q2^2)^2 +
2*p1^4*p2*q2*(p2^2 + q2^2) + 2*p2*q1^2*(-1 + q1^2)*q2*(p2^2 + q2^2) +
p1*q1*(p2 - q2)^2*(1 + p2^2*(-2 + q1^2) + 2*p2*q1^2*q2 +
(-2 + q1^2)*q2^2) + 2*p1^2*(p2^4*q1^2 + 6*p2^2*q1^2*q2^2 -
p2*(1 + 2*q1^2)*q2^3 + q1^2*q2^4 - p2^3*(q2 + 2*q1^2*q2)))) +
2*b2*b3^2*(-2*p1^4*p2*q2*(p2^2 + q2^2) - 2*p2*q1^2*(-1 + q1^2)*q2*
(p2^2 + q2^2) + 2*p1^2*p2*q2*(p2^2*(1 + 2*q1^2) - 8*p2*q1^2*q2 +
(1 + 2*q1^2)*q2^2) + p1^3*q1*(p2^4 + 2*p2*q2 + q2^2*(-1 + q2^2) +
p2^2*(-1 + 10*q2^2)) + p1*q1*(p2^4*q1^2 - 4*p2^3*q2 +
2*p2*q2*(q1^2 - 2*q2^2) + q1^2*q2^2*(-1 + q2^2) +
p2^2*q1^2*(-1 + 10*q2^2))) + 4*b2*p1*p2*q1*q2*
(2*b2^2*p2*q2 + sigm^2) - b1*p1*q1*(p2 - q2)*
(-2*b2*b3*(p1^2 + q1^2)*(-1 + p2^2 + 2*p2*q2 + q2^2) +
b3^2*(p1 - q1)*(1 + p2^2*(-2 + p1^2 + 2*p1*q1 + q1^2) -
2*p2*(p1 + q1)^2*q2 + (-2 + p1^2 + 2*p1*q1 + q1^2)*q2^2) +
(p1 - q1)*sigm^2) - b3*(p1 - q1)*
(2*b2^2*p2*q2*(p2^2*(-1 + p1^2 + 2*p1*q1 + q1^2) - 12*p1*p2*q1*q2 +
(-1 + p1^2 + 2*p1*q1 + q1^2)*q2^2) +
(p2^2*(-1 + p1^2 + 3*p1*q1 + q1^2) - 6*p1*p2*q1*q2 +
(-1 + p1^2 + 3*p1*q1 + q1^2)*q2^2)*sigm^2)) -
2*b1*b3*p2*(b2 + b3*(p1 - q1))^2*q2*
(-2*b2^3*p2*(p1 - q1)*q2*(p2^2*(-1 + p1^2 + 2*p1*q1 + q1^2) -
4*p1*p2*q1*q2 + (-1 + p1^2 + 2*p1*q1 + q1^2)*q2^2) -
4*b2^2*b3*p2*q2*(p1^4*(p2^2 + q2^2) + q1^2*(-1 + q1^2)*(p2^2 + q2^2) +
p1^3*q1*(p2^2 - 6*p2*q2 + q2^2) + p1*q1*(p2^2*(1 + q1^2) -
6*p2*q1^2*q2 + (1 + q1^2)*q2^2) - p1^2*(p2^2*(1 + 2*q1^2) -
8*p2*q1^2*q2 + (1 + 2*q1^2)*q2^2)) - b1*p1*q1*(p2 - q2)*
(-8*b2^2*p1*p2*q1*q2 + 2*b2*b3*(p1 - q1)*
(1 + p2^2*(-2 + p1^2 + 2*p1*q1 + q1^2) - 2*p2*(p1^2 + 6*p1*q1 + q1^2)*
q2 + (-2 + p1^2 + 2*p1*q1 + q1^2)*q2^2) + b3^2*(p1^2 + q1^2)*
(1 + p2^2*(-2 + p1^2 + 2*p1*q1 + q1^2) - 2*p2*(p1^2 + 6*p1*q1 + q1^2)*
q2 + (-2 + p1^2 + 2*p1*q1 + q1^2)*q2^2) + (p1^2 - 4*p1*q1 + q1^2)*
sigm^2) - b2*(p1 - q1)*(2*b3^2*(p1^4*p2*q2*(p2^2 + q2^2) +
p2*q1^2*(-1 + q1^2)*q2*(p2^2 + q2^2) + p1^3*q1*(p2 - q2)^2*
(p2^2 + 4*p2*q2 + q2^2) + p1^2*(2*p2^4*q1^2 + 12*p2^2*q1^2*q2^2 -
p2*(1 + 6*q1^2)*q2^3 + 2*q1^2*q2^4 - p2^3*(q2 + 6*q1^2*q2)) +
p1*q1*(p2^4*(-2 + q1^2) + 2*p2^3*q1^2*q2 + q2^2 + (-2 + q1^2)*q2^4 +
2*p2*q2*(-1 + q1^2*q2^2) + p2^2*(1 - 2*(2 + 3*q1^2)*q2^2))) +
(p2^2*(-1 + p1^2 + 2*p1*q1 + q1^2) - 4*p1*p2*q1*q2 +
(-1 + p1^2 + 2*p1*q1 + q1^2)*q2^2)*sigm^2) -
b3*p1*q1*(b3^2*(2*p1^3*q1*(p2 - q2)^4 + 2*p1*q1^3*(p2 - q2)^4 +
p1^4*(p2^2 - q2^2)^2 + p1^2*(2*p2^4*(-1 + q1^2) - 2*p2*q2 + q2^2 +
```

```
2*(-1 + q1^2)*q2^4 + p2^2*(1 - 4*(1 + q1^2)*q2^2)) +
q1^2*(p2^4*(-2 + q1^2) - 2*p2*q2 + q2^2 + (-2 + q1^2)*q2^4 +
p2^2*(1 - 2*(2 + q1^2)*q2^2))) + (p2^2*(-2 + 3*p1^2 + 3*q1^2) -
6*p2*(p1^2 + q1^2)*q2 + (-2 + 3*p1^2 + 3*q1^2)*q2^2)*sigm^2)) +
b3^2*p2*(b2 + b3*(p1 - q1))^2*q2*
(b1*p1*q1*(8*b2^2*p2*q2*(p1^2*p2*q2 + p2*q1^2*q2 - p1*q1*(p2^2 + q2^2)) +
b3^2*(p1^4*(p2 - q2)^2*(p2^2 + q2^2) + 2*p1^3*q1*(p2^4 - 6*p2^3*q2 +
2*p2^2*q2^2 - 6*p2*q2^3 + q2^4) + 2*p1*q1^3*(p2^4 - 6*p2^3*q2 +
2*p2^2*q2^2 - 6*p2*q2^3 + q2^4) + q1^2*(-p2^2 + p2^4*q1^2 +
4*p2*q2 - 2*p2^3*q1^2*q2 - q2^2 + 2*p2^2*q1^2*q2^2 -
2*p2*q1^2*q2^3 + q1^2*q2^4) + p1^2*(-p2^2 + 2*p2^4*q1^2 +
4*p2*q2 - 4*p2^3*q1^2*q2 - q2^2 + 4*p2^2*q1^2*q2^2 -
4*p2*q1^2*q2^3 + 2*q1^2*q2^4)) +
2*b2*b3*(p1^3*(p2 - q2)^2*(p2^2 + q2^2) +
p1^2*q1*(p2^4 - 10*p2^3*q2 + 2*p2^2*q2^2 - 10*p2*q2^3 + q2^4) +
q1*(-(p2^4*q1^2) + 2*p2^3*q1^2*q2 + q2^2 - q1^2*q2^4 +
p2^2*(1 - 2*q1^2*q2^2) + 2*p2*q2*(-2 + q1^2*q2^2)) -
p1*(p2^4*q1^2 - 10*p2^3*q1^2*q2 + q2^2 + q1^2*q2^4 +
p2^2*(1 + 2*q1^2*q2^2) - 2*p2*q2*(2 + 5*q1^2*q2^2))) +
(p1^2 - 4*p1*q1 + q1^2)*(p2^2 + q2^2)*sigm^2) +
(p2 - q2)*(2*b2^3*p2*(p1 - q1)*q2*(-1 + p2^2*q1^2 + 2*p2*q1^2*q2 +
q1^2*q2^2 + p1^2*(p2 + q2)^2 + 2*p1*q1*(p2^2 + q2^2)) +
4*b2^2*b3*p2*q2*(p1*(q1 + q1^3*(p2 - q2)^2) + p1^3*q1*(p2 - q2)^2 +
p1^4*(p2 + q2)^2 + q1^2*(-1 + p2^2*q1^2 + 2*p2*q1^2*q2 +
q1^2*q2^2) - p1^2*(1 + 2*p2^2*q1^2 - 4*p2*q1^2*q2 +
2*q1^2*q2^2)) + b3*p1*q1*(b3^2*(p1^2 + q1^2)*(p2^2 + q2^2)*
(-1 + p2^2*q1^2 + 2*p1*q1*(p2 - q2)^2 + 2*p2*q1^2*q2 + q1^2*q2^2 +
p1^2*(p2 + q2)^2) + (-2 + 3*p2^2*q1^2 + 3*q1^2*q2^2 +
3*p1^2*(p2^2 + q2^2))*sigm^2) + b2*(p1 - q1)*
(2*b3^2*(p1^4*p2*q2*(p2 + q2)^2 + p2*q1^2*q2*(-1 + p2^2*q1^2 +
2*p2*q1^2*q2 + q1^2*q2^2) + p1*q1*(p2^2 + q2^2)*
(-1 + p2^2*q1^2 + 4*p2*q1^2*q2 + q1^2*q2^2) +
p1^3*q1*(p2^4 + 4*p2^3*q2 + 2*p2^2*q2^2 + 4*p2*q2^3 + q2^4) +
p1^2*(2*p2^4*q1^2 - 2*p2^3*q1^2*q2 + 8*p2^2*q1^2*q2^2 +
2*q1^2*q2^4 - p2*(q2 + 2*q1^2*q2^3))) +
(-1 + p2^2*q1^2 + 2*p2*q1^2*q2 + q1^2*q2^2 + p1^2*(p2 + q2)^2 +
2*p1*q1*(p2^2 + q2^2))*sigm^2))) + b3*(b2 + b3*(p1 - q1))*
(2*b1^2*p1*q1 + 4*b1*b3*p1*q1*(p2 - q2) + 2*b3*p2*(b2 + b3*(p1 - q1))*
(p1 - q1)*q2 + 2*b3^2*p1*q1*(p2^2 + q2^2) + sigm^2)*
(b1*p1*q1*(-2*b2*b3*(p1^2 + q1^2)*(p2^4 - 2*p2^3*q2 -
2*p2*q2*(-2 + q2^2) + q2^2*(-1 + q2^2) + p2^2*(-1 + 2*q2^2)) +
b3^2*(-(p1^3*(p2 - q2)^2*(p2^2 + q2^2)) - p1^2*q1*(p2 - q2)^2*
(p2^2 + q2^2) + p1*(p2^4*q1^2 - 2*p2^3*q1^2*q2 + q2^2 + q1^2*q2^4 -
2*p2*q2*(2 + q1^2*q2^2) + p2^2*(1 + 2*q1^2*q2^2)) +
q1*(p2^4*q1^2 - 2*p2^3*q1^2*q2 + q2^2*(-1 + q1^2*q2^2) +
p2^2*(-1 + 2*q1^2*q2^2) + p2*(4*q2 - 2*q1^2*q2^3))) -
(p1 - q1)*(8*b2^2*p2^2*q2^2 + (p2^2 + q2^2)*sigm^2)) -
(p2 - q2)*(2*b2*b3^2*(p1^4*p2*q2*(1 + p2^2 + 2*p2*q2 + q2^2) +
p2*q1^2*q2*(-2 + q1^2*(1 + p2^2 + 2*p2*q2 + q2^2)) +
p1^3*q1*(p2^4 + 2*p2^3*q2 + 2*p2*q2^3 + q2^2*(-1 + q2^2) -
p2^2*(1 + 2*q2^2)) - 2*p1^2*p2*q2*
(1 + q1^2*(-1 + 3*p2^2 - 2*p2*q2 + 3*q2^2)) +
p1*(p2^4*q1^3 + 2*p2^3*q1^3*q2 + q1^3*q2^2*(-1 + q2^2) -
p2^2*q1^3*(1 + 2*q2^2) + 2*p2*q1*q2*(2 + q1^2*q2^2))) +
b3^3*(p1 - q1)*(2*p1^4*p2*q2 + 2*p2*q1^2*(-1 + q1^2)*q2 +
p1^3*q1*(p2 + q2)^2*(p2^2 + q2^2) +
```

```
p1*q1*(p2^4*q1^2 + 2*p2^3*q1^2*q2 + q2^2*(-1 + q1^2*q2^2) +
2*p2*q2*(2 + q1^2*q2^2) + p2^2*(-1 + 2*q1^2*q2^2)) +
2*p1^2*(p2^4*q1^2 - 2*p2^3*q1^2*q2 + 2*p2^2*q1^2*q2^2 + q1^2*q2^4 -
p2*(q2 - 2*q1^2*q2 + 2*q1^2*q2^3))) +
b2*(-4*p1*p2*q1*q2 + p1^2*(-1 + p2^2 + 2*p2*q2 + q2^2) +
q1^2*(-1 + p2^2 + 2*p2*q2 + q2^2))*(2*b2^2*p2*q2 + sigm^2) +
b3*(p1 - q1)*(2*b2^2*p2*q2*(-1 + 2*p1*q1*(p2 - q2)^2 +
p1^2*(-1 + 2*p2^2 + 4*p2*q2 + 2*q2^2) +
q1^2*(-1 + 2*p2^2 + 4*p2*q2 + 2*q2^2)) +
(-1 + p1^2 + q1^2 + 3*p1*q1*(p2^2 + q2^2))*sigm^2)))))/
(2*sqrt(B)*(p1*q1*(2*b2^2*p2*q2 + 4*b2*b3*p2*(p1 - q1)*q2 +
2*b3^2*p2*(p1^2 + q1^2)*q2 + sigm^2))^(3/2)*
sqrt((p2*(b2 + b3*(p1 - q1))^2*q2)/(2*b1^2*p1*q1 +
4*b1*b3*p1*q1*(p2 - q2) + 2*b3^2*p1*q1*(p2^2 + q2^2) + sigm^2))*
(2*b1^2*p1*q1 + 4*b1*b3*p1*q1*(p2 - q2) + 2*b3^2*p1*q1*(p2^2 + q2^2) +
sigm^2)^2)
```
